# The plasma proteome is linked with left ventricular and left atrial function parameters in patients with chronic heart failure

**DOI:** 10.1093/ehjci/jeae098

**Published:** 2024-04-10

**Authors:** S Abou Kamar, K Andrzejczyk, T B Petersen, J F Chin, Y S Aga, M de Bakker, K M Akkerhuis, M Geleijnse, J J Brugts, O Sorop, R A de Boer, D Rizopoulos, F W Asselbergs, E Boersma, H den Ruijter, B M van Dalen, I Kardys

**Affiliations:** Department of Cardiology, Erasmus MC, Thorax Center, Cardiovascular Institute, University Medical Center Rotterdam, Room Na-316, PO Box 2040 3000 CA Rotterdam, The Netherlands; The Netherlands Heart Institute, Moreelsepark 1, 3511 EP Utrecht, The Netherlands; Department of Cardiology, Franciscus Gasthuis & Vlietland, Kleiweg 500, 3045 PM Rotterdam, The Netherlands; Department of Cardiology, Erasmus MC, Thorax Center, Cardiovascular Institute, University Medical Center Rotterdam, Room Na-316, PO Box 2040 3000 CA Rotterdam, The Netherlands; Department of Cardiology, Erasmus MC, Thorax Center, Cardiovascular Institute, University Medical Center Rotterdam, Room Na-316, PO Box 2040 3000 CA Rotterdam, The Netherlands; Department of Biostatistics, University Medical Center Rotterdam, Erasmus University, Dr. Molewaterplein 40, 3000 CA Rotterdam, The Netherlands; Department of Cardiology, Erasmus MC, Thorax Center, Cardiovascular Institute, University Medical Center Rotterdam, Room Na-316, PO Box 2040 3000 CA Rotterdam, The Netherlands; Department of Cardiology, Franciscus Gasthuis & Vlietland, Kleiweg 500, 3045 PM Rotterdam, The Netherlands; Department of Cardiology, Erasmus MC, Thorax Center, Cardiovascular Institute, University Medical Center Rotterdam, Room Na-316, PO Box 2040 3000 CA Rotterdam, The Netherlands; Department of Cardiology, Franciscus Gasthuis & Vlietland, Kleiweg 500, 3045 PM Rotterdam, The Netherlands; Department of Cardiology, Erasmus MC, Thorax Center, Cardiovascular Institute, University Medical Center Rotterdam, Room Na-316, PO Box 2040 3000 CA Rotterdam, The Netherlands; Department of Cardiology, Erasmus MC, Thorax Center, Cardiovascular Institute, University Medical Center Rotterdam, Room Na-316, PO Box 2040 3000 CA Rotterdam, The Netherlands; Department of Cardiology, Erasmus MC, Thorax Center, Cardiovascular Institute, University Medical Center Rotterdam, Room Na-316, PO Box 2040 3000 CA Rotterdam, The Netherlands; Department of Cardiology, Erasmus MC, Thorax Center, Cardiovascular Institute, University Medical Center Rotterdam, Room Na-316, PO Box 2040 3000 CA Rotterdam, The Netherlands; Department of Cardiology, Erasmus MC, Thorax Center, Cardiovascular Institute, University Medical Center Rotterdam, Room Na-316, PO Box 2040 3000 CA Rotterdam, The Netherlands; Department of Cardiology, Erasmus MC, Thorax Center, Cardiovascular Institute, University Medical Center Rotterdam, Room Na-316, PO Box 2040 3000 CA Rotterdam, The Netherlands; Department of Cardiology, Erasmus MC, Thorax Center, Cardiovascular Institute, University Medical Center Rotterdam, Room Na-316, PO Box 2040 3000 CA Rotterdam, The Netherlands; Department of Biostatistics, University Medical Center Rotterdam, Erasmus University, Dr. Molewaterplein 40, 3000 CA Rotterdam, The Netherlands; Department of Cardiology, Amsterdam Medical Center, Meibergdreef 9, 1105 AZ Amsterdam, The Netherlands; Department of Cardiology, Erasmus MC, Thorax Center, Cardiovascular Institute, University Medical Center Rotterdam, Room Na-316, PO Box 2040 3000 CA Rotterdam, The Netherlands; Laboratory of Experimental Cardiology, University Medical Center Utrecht, Heidelberglaan 100, 3584 CX Utrecht, The Netherlands; Department of Cardiology, Erasmus MC, Thorax Center, Cardiovascular Institute, University Medical Center Rotterdam, Room Na-316, PO Box 2040 3000 CA Rotterdam, The Netherlands; Department of Cardiology, Franciscus Gasthuis & Vlietland, Kleiweg 500, 3045 PM Rotterdam, The Netherlands; Department of Cardiology, Erasmus MC, Thorax Center, Cardiovascular Institute, University Medical Center Rotterdam, Room Na-316, PO Box 2040 3000 CA Rotterdam, The Netherlands

**Keywords:** proteomics, heart failure, echocardiography, strain parameters, pathophysiological processes

## Abstract

**Aims:**

Examining the systemic biological processes in the heterogeneous syndrome of heart failure with reduced ejection fraction (HFrEF), as reflected by circulating proteins, in relation to echocardiographic characteristics, may provide insights into heart failure pathophysiology. We investigated the link of 4210 repeatedly measured circulating proteins with repeatedly measured echocardiographic parameters as well as with elevated left atrial pressure (LAP), in patients with HFrEF, to provide insights into underlying mechanisms.

**Methods and results:**

In 173 patients with HFrEF, we performed 6-monthly echocardiography and trimonthly blood sampling during a median follow-up of 2.7 (inter-quartile range: 2.5–2.8) years. We investigated circulating proteins in relation to echocardiographic parameters of left ventricular [left ventricular ejection fraction (LVEF), global longitudinal strain (GLS)] and left atrial function [left atrial reservoir strain (LASr)] and elevated LAP (*E*/*e*ʹ ratio >15) and used gene enrichment analyses to identify underlying pathophysiological processes. We found 723, 249, 792, and 427 repeatedly measured proteins, with significant associations with LVEF, GLS, LASr, and *E*/*e*ʹ ratio, respectively. Proteins associated with LASr reflected pathophysiological mechanisms mostly related to the extracellular matrix. Proteins associated with GLS reflected cardiovascular biological processes and diseases, whereas those associated with LVEF reflected processes involved in the sympathetic nervous system. Moreover, 49 proteins were associated with elevated LAP; after correction for LVEF, three proteins remained: cystatin-D, fibulin-5, and HSP40.

**Conclusion:**

Circulating proteins show varying associations with different echocardiographic parameters in patients with HFrEF. These findings suggest that pathways involved in atrial and ventricular dysfunction, as reflected by the plasma proteome, are distinct.

## Graphical Abstract

**Graphical Abstract:**
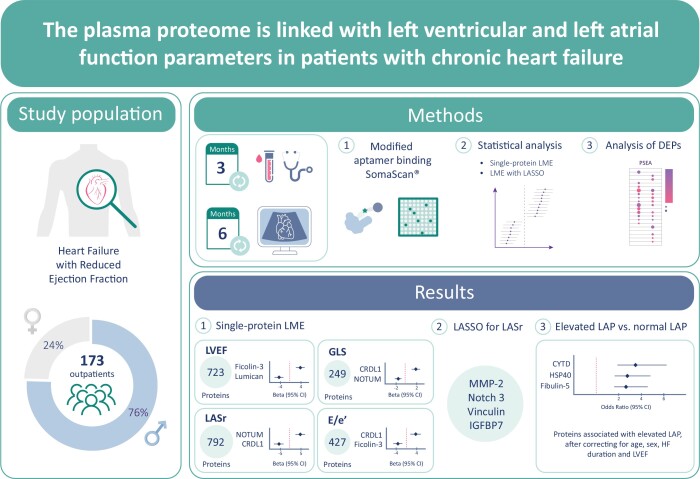

## Introduction

Despite the improvements in treatment over the last decades, the prognosis of heart failure (HF) remains poor.^1,2^ While several risk factors for death and HF hospitalization have been established, adequate risk stratification of patients with heart failure with reduced ejection fraction (HFrEF) remains challenging. Moreover, numerous aspects of the biological processes underlying this heterogeneous condition remain to be elucidated.

In routine clinical practice, risk stratification in HFrEF is mainly based on echocardiographic parameters, especially left ventricular ejection fraction (LVEF). More recently, strain parameters such as global longitudinal strain (GLS) and left atrial reservoir strain (LASr) have also gained attention as prognostic markers in HFrEF.^3,4^ Furthermore, elevated left atrial pressure (LAP) commonly occurs in patients with HFrEF and can be a sign of disease progression or a trigger of worsening HF.^5^ The ratio of the peak early left ventricular (LV) filling velocity and early diastolic mitral annular velocity (*E*/*e*ʹ ratio) is the most frequently used echocardiographic parameter to estimate LAP.^4,5^

Previously, we have confirmed the prognostic value of serial measurements of LVEF as well as GLS, LASr, and *E*/*e*ʹ in patients with HFrEF, and we have described the temporal evolutions of these parameters during several years of follow-up. We demonstrated that, in patients who experienced incident adverse cardiovascular events, all parameters were decreased but remained stable over time as the adverse event approached, when compared with patients who remained event-free during follow-up.^6–8^

However, the pathophysiological mechanisms underlying the associations of these echocardiographic parameters with disease evolution in HFrEF have not yet been fully elucidated. Examining systemic biological mechanisms at work in HF in relation to echocardiographic abnormalities may enable further unravelling of the aetiology of the condition and provide insights relevant for treatment.^9^ Modern laboratory technologies, including robust affinity-based methods, currently provide us with the opportunity to measure large numbers of circulating proteins that may be related to specific organ systems but may also reflect more general biological processes.^10^ As such, measuring thousands of proteins simultaneously in a small biological sample provides a comprehensive overview of the patient’s health state.^11^ Also, assessment of such circulating proteins allows us to investigate subclinical processes and mechanisms underlying disease progression in HF patients that may accompany echocardiographic changes.

This study aimed to investigate biological mechanisms underlying functional cardiac changes as determined by echocardiography in patients with HFrEF and specifically to investigate the associations between 4210 repeatedly measured circulating proteins and repeatedly measured echocardiographic parameters of LV and left atrial (LA) function as well as LAP.

## Methods

### Study design

For this study, data were used from the Bio-SHiFT study (Serial Biomarker Measurements and New Echocardiographic Techniques in Chronic Heart Failure Patients Result in Tailored Prediction of Prognosis). Details on the design of the Bio-SHiFT study have been published previously.^12^ In short, Bio-SHiFT is a prospective, observational cohort of stable patients with chronic HF, conducted in the Erasmus MC, Rotterdam, and Northwest Clinics, Alkmaar, The Netherlands. The main inclusion criteria were diagnosis of HF according to the then prevailing guidelines of the European Society of Cardiology and age ≥18 years. Patients were recruited during their regular outpatient visits while in a clinically stable condition (i.e. they had not been hospitalized for HF in the 3 months before inclusion). All patients were evaluated by research physicians, who collected information on HF-related symptoms and the New York Heart Association (NYHA) class and performed a physical examination. Information on HF aetiology, LVEF, cardiovascular risk factors, medical history, and treatment was retrieved primarily from hospital records. Patients were followed for a maximum of 30 months by trimonthly study visits. The clinical study endpoint was the composite of cardiovascular mortality, HF hospitalization, LV assist device implantation, and heart transplantation. A total of 398 patients were included in the entire Bio-SHiFT cohort. Out of them, 175 patients were included in an echocardiography substudy at the Erasmus MC, of whom two patients had insufficient image quality for reliable assessment of echocardiographic parameters, leaving a total of 173 patients for the substudy. All the patients from the Erasmus MC were eligible to enter the echocardiographic substudy. The study was approved by the medical ethics committees (Erasmus MC), conducted in accordance with the Declaration of Helsinki, and registered in ClinicalTrials.gov (NCT01851538). All patients signed informed consent for the study.

### Sample collection and processing

Blood samples were collected at baseline and at each study follow-up visit. Within 2 h after collection, blood samples were processed, and ethylenediaminetetraacetic acid plasma was stored at −80°C.^12^ Accordingly, at the time of the outpatient visits, results of the proteomic analysis were not available to treating physicians. Laboratory personnel were blinded for clinical data and patient outcomes.

All baseline blood samples were selected. Additionally, we selected the last two samples drawn before the occurrence of the clinical study endpoint or the last two samples taken before censoring for patients who remained endpoint-free. By selecting the last two samples before the study endpoint, we aimed to capture any changes in protein levels preceding the adverse event, while improving efficiency (see Supplementary data online, *Figure S1*).

### Proteomic measurements

The aptamer-based proteomic SOMAscan platform (Somalogic, USA) was used to measure the circulating proteins.^13^ SOMAscan utilizes single-stranded DNA-based protein affinity reagents called SOMAmers (Slow Off-rate Modified Aptamers). SOMAmers minimize the non-specific binding interactions, by only binding proteins with high specificity and affinity, and slow dissociation rates. The readout of the SOMAscan is in normalized relative fluorescent units (RFUs), which are intensities proportional to the amount of target protein in the original sample. Previous studies reported high assay reproducibility and low technical variability of SOMAscan^14^ (see Supplementary data online, *Material S1*).

Somalogic’s previously described standard processes for normalization, calibration, and quality control were followed.^14^ Resultantly, out of the total 5284 modified aptamers, aptamers against 4210 proteins were included in the analyses (see Supplementary data online, *Material S1*).

### Echocardiography measurements and evaluation

Two-dimensional grey-scale harmonic images were obtained in the left lateral decubitus position. Standard apical four-, three-, and two-chamber views were recorded. A commercially available ultrasound system was used (iE33, Philips, Best, The Netherlands), equipped with a broadband (1–5 MHz) S5-1 transducer (frequency transmitted 1.7 MHz and received 3.4 MHz). Images were stored in the echo core lab of Erasmus MC. Using specialized software (2D Cardiac Performance Analysis, version 4.5, TomTec Imaging Systems, Unterschleissheim, Germany), and the systolic parameters were assessed. The diastolic parameters were evaluated using Philips Excellera, version R4.1 (Philips Medical Systems, The Netherlands), or TomTec Imaging Systems, according to the ASE/EACVI guidelines.^12^*E*/*e*′ > 15 was used to dichotomize patients in those with or without increased LAP.^5^ If patients had atrial fibrillation (AF) on the echo, the index beat method was used. This is a validated method to measure echocardiographic parameters during AF.^15^

Strain analysis based on speckle tracking echocardiography was also performed using TomTec Imaging Systems. The images were analysed retrospectively by a single operator, who was blinded to other echocardiographic parameters and the patients’ characteristics, after completion of follow-up. The exact methods of the strain analysis have been described in detail previously.^6–8^ Further details on the echocardiographic evaluation are provided in Supplementary data online, *Material S2*.

### Statistical analyses

A full description of the statistical analyses is given in Supplementary data online, *Material S3*.

In short, all protein levels were log-transformed, and subsequently, the *Z*-score was calculated. The timing of echocardiography and blood sampling did not necessarily coincide (see Supplementary data online, *Figure S1*). Therefore, linear mixed-effect (LME) models were first fitted to describe the temporal evolvement of the measured circulating proteins, and the obtained LME models were then used to estimate the protein level at the moment of the echocardiography. Then, we fitted the models of interest (repeatedly measured echoparameter as a dependent variable and protein as an independent variable). We adjusted for sex, age, and duration of HF and, in the analyses for LASr and *E*/*e*ʹ ratio, also for LVEF. We also corrected for multiple testing using the Benjamini–Hochberg method (false discovery rate [FDR] < 0.05).

To determine which proteins remain statistically significantly associated with the echoparameters in a multivariable setting, we used LME models with least absolute shrinkage and selection operator (LASSO). For the selection of the optimal lambda term, a 10-fold cross-validation of the model was performed. Since the folds for the 10-fold cross-validation are selected randomly, we repeated the LASSO regression and the 10-fold cross-validation ten times. The proteins that were present in ≥8 of the folds were considered relevant. We adjusted for sex, age, and duration of HF and, in the analyses for LASr and *E*/*e*ʹ ratio, also for LVEF.

All the circulating proteins that were significantly associated with echoparameters in the single-protein analyses were further analysed using protein enrichment analysis in ToppGene, with all 4210 proteins being used as the background set. ToppGene is a tool for interpreting gene expression data and focuses on gene sets, i.e. groups of genes that share common biological and molecular functions, and pathways.^16^

Differences in protein levels between patients with elevated LAP and normal LAP were analysed using logistic regression, adjusted for age, sex, and duration of HF. In a second step, we adjusted for LVEF to account for the role of systolic dysfunction in the elevated LAP group. We also corrected for multiple testing (FDR < 0.05). We calculated odds ratios (ORs) with 95% confidence intervals (CIs) per 1 SD difference in the log-transformed protein levels and presented the proteins that showed associations with LAP after adjustment for multiple testing in forest plots.

All analyses were performed with R statistical software using packages glmmLasso^17^ and nlme.^18^ All tests were two-tailed, and *P* values < 0.05 were considered statistically significant.

## Results

### Baseline characteristics and follow-up

Of the 173 patients used for the analysis, 76% were male, mean (±SD) age was 58 ± 11 years, and 81% were in NYHA class I/II. Patients with elevated LAP were significantly older, had a higher NT-proBNP, used more loop diuretics, and had more comorbidities (*Table 1*). LVEF, GLS, and LASr were significantly lower (mean ± SD: 31.3 ± 10.0 vs. 24.9 ± 9.8%, −10.3 ± 3.5 vs. −7.3 ± 2.9%, and 25.8 ± 9.9 vs. 14.4 ± 8.82, respectively) in patients with elevated LAP (*Table 2*).

**Table 1:** Baseline characteristics of the total cohort (*n* = 173) and of the patients where LAP could be estimated (*n* = 152)

	Total	Normal LAP (*E*/*e*′ ≤ 15)^a^	Elevated LAP (*E*/*e*′ > 15)^a^	*P*-value
*n*	173	88	64	
Demographics				
Male, %	132(76)	69(78)	49(77)	0.9
Age, years	58.0 ± 11.2	56 ± 11	60 ± 11	0.04
Clinical characteristics				
Duration of HF, years	6.8 (6.3–7.3)	6.0 (5.8–6.4)	6.9 (6.4–7.5)	
Body mass index, kg/m²	27.5 ± 4.7	27.5 ± 5.1	27.8 ± 4.0	0.8
Heart rate, bpm	67 ± 13	66 ± 15	68 ± 9	0.4
Systolic blood pressure, mmHg	108 ± 18	111 ± 19	105 ± 16	0.06
Diastolic blood pressure, mmHg	67 ± 10	69 ± 10	67 ± 9	0.2
NYHA class, %				0.6
I	45(26.3)	24(28)	14(22)	
II	94(55)	49(56)	35(56)	
III	32(19)	14(16)	14(22)	
NT-proBNP, pmol/L	118 (31–223)	67 (21–174)	235 (134–404)	<0.001
Aetiology of HF				
Ischaemic heart disease, %	71(41)	26(30)	33(52)	0.01
Cardiomyopathy, %	73(42)	45(51)	22(34)	0.06
Of which familial	25(34)	16(36)	7(32)	0.07
Type of cardiomyopathy				
Hypertrophic cardiomyopathy, %	6(8)	3(7)	2(9)	0.1
Dilated cardiomyopathy, %	60(82)	35(78)	20(91)	0.06
Non-compaction cardiomyopathy, %	4(6)	4(9)	0(0)	0.06
Unclassified cardiomyopathy, %	3(4)	3(7)	0(0)	0.06
Secondary to valvular heart disease, %	4(2)	1(1)	3(5)	0.4
Hypertension, %	2(1)	2(2)	0(0)	0.6
Unknown, %	9(5)	7(8)	0(0)	0.06
Medical history				
Myocardial infarction, %	69(40)	24(27)	33(52)	0.004
Chronic renal failure, %	69(40)	22(25)	38(59)	<0.001
PCI, %	62(36)	24(27)	28(44)	0.05
Atrial fibrillation, %	53(31)	18(21)	28(44)	0.004
Diabetes mellitus, %	40(23)	14(16)	20(31)	0.06
COPD, %	24(14)	8(9)	12(19)	0.1
CABG, %	16(9)	7(8)	7(11)	0.7
Medication use				
Beta blockers, %	165(95)	86(98)	59(92)	0.2
Loop diuretics, %	161(93)	77(88)	64(100)	0.009
Aldosteron antagonists, %	128(74)	59(67)	52(81)	0.08
ACE inhibitors, %	120(69)	68(77)	39(61)	0.05
Angiotensin II receptor blockers, %	48(28)	19(22)	22(34)	0.1

Normally distributed data are presented as mean ± SD, whereas non-normally distributed data are presented as median (25th–75th percentiles).

HF, heart failure; NYHA, New York Heart Association; PCI, percutaneous coronary intervention; CABG, coronary artery bypass graft; COPD, chronic obstructive pulmonary disease; ACE, angiotensin-converting enzyme.

^a^LAP could be estimated in 152 patients.

**Table 2:** Echocardiographic characteristics of first available echo of the patients where LAP could be estimated

	Overall	Normal LAP (*E*/*e*′ ≤ 15)^a^	Elevated LAP (*E*/*e*′ > 15)^a^	*P*-value
*n*	173	88	64	
Systolic parameters				
GLS, %	−9.0 ± 3.7	−10.3 ± 3.5	−7.3 ± 2.9	<0.001
LVEF, %	29.1 ± 10.4	31.3 ± 10.0	24.9 ± 9.8	<0.001
Systolic LV diameter, mm	56.6 (48.9–64.0)	53.0 (45.8–62.0)	59.0 (53.2–67.0)	<0.001
Systolic LA diameter, mm	42.7 ± 8.4	39.6 ± 7.6	46.8 ± 7.6	<0.001
Diastolic parameters				
Diastolic LV diameter, mm	63.0 (57.0–70.0)	62.0 (56.0–70.8)	66.5 (63.0–75.3)	0.001
E/A ratio	1.4 ± 1.0	1.1 ± 0.6	2.03 ± 1.30	<0.001
*E*/*e*′ ratio	15.6 (9.5–19.7)	9.6 (4.6–14.9)	23.5 (15.8–26.9)	<0.001
LASr, %	20.9 ± 11.3	25.8 ± 9.9	14.4 ± 8.82	<0.001
TR velocity, m/s	2.5 (2.1–2.9)	2.4 (2.0–2.6)	2.6 (2.3–3.1)	0.005

Normally distributed data are presented as mean ± SD, whereas non-normally distributed data are presented as median (25th–75th percentiles).

GLS, left ventricular global longitudinal strain; LVEF, left ventricular ejection fraction; LV, left ventricle; LA, left atrium; *E*/*A* ratio, the ratio of the peak early LV filling velocity over the late filling velocity; *E*/*e*ʹ ratio, the ratio of the *E* to early diastolic mitral annular tissue velocity; LASr, left atrial reservoir strain; TR, tricuspid regurgitation.

^a^LAP could be estimated in 152 patients.

During a median follow-up time of 2.7 [inter-quartile range (IQR): 2.5–2.8] years, 505 echocardiograms were performed with a median of 3 (IQR: 2–4) echoes per patient. Overall, the echocardiographic parameters (LVEF, GLS, LASr, and *E*/*e*ʹ) were stable over time during this follow-up period, with no clinically relevant increase or decrease in this timeframe (see Supplementary data online, *Figure S2*).

### Associations of repeatedly measured circulating proteins with repeatedly measured echoparameters

**Figure 1:**
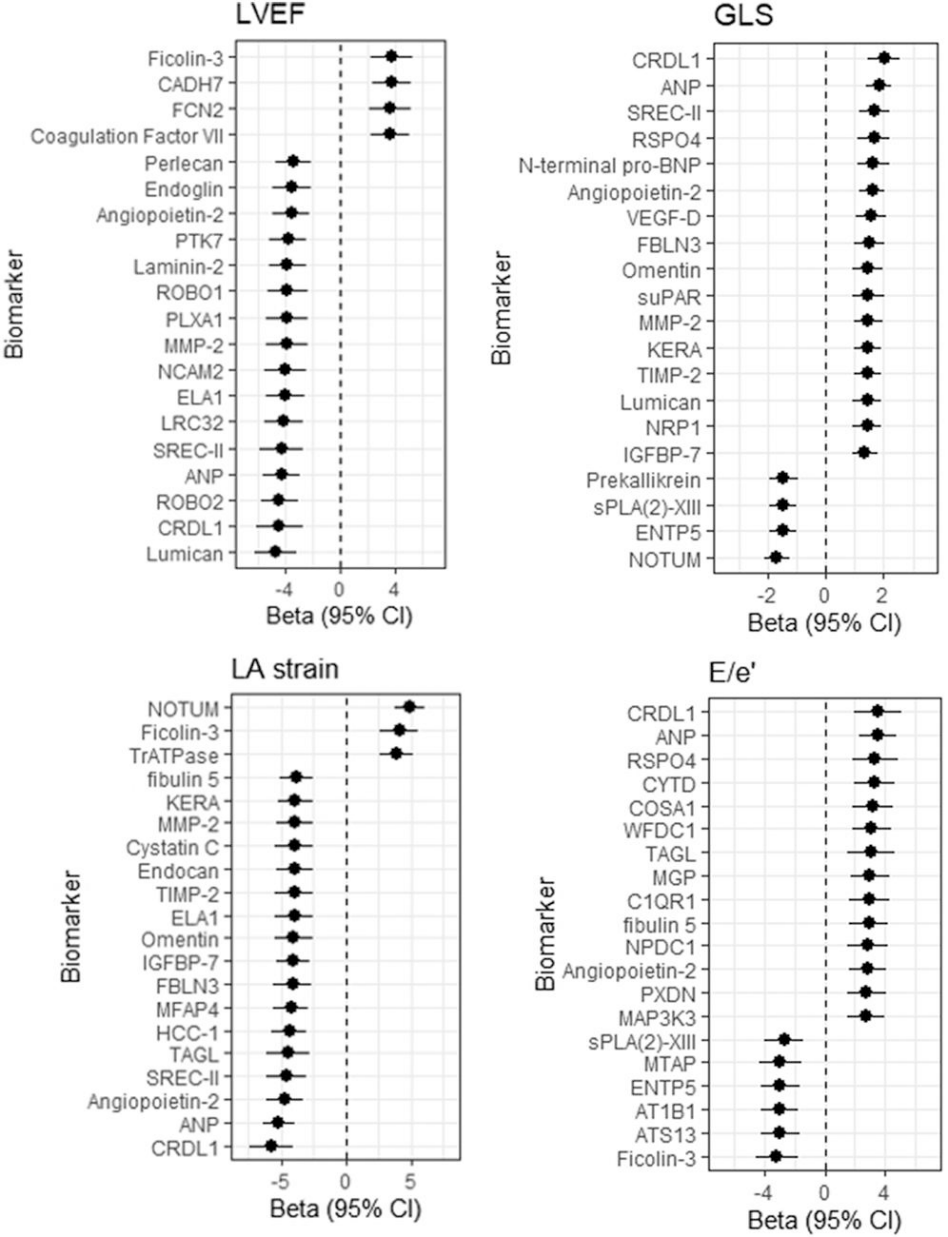
Univariable associations (adjusted for age, sex, and duration of HF) between the repeatedly measured circulating proteins in relation to repeatedly measured echoparameters, resulting from the univariable LME models, with the echoparameter as the dependent variable and the protein as the independent variable (top 20). For LASr and *E*/*e*ʹ, we also adjusted for LVEF.

The single-protein LME models (adjusted for age, sex, duration of HF, and for LVEF in the cases of LASr and *E*/*e*ʹ; and corrected for multiple testing) showed significant associations between repeatedly measured levels of circulating proteins and repeatedly measured echocardiographic parameters of LV function (LVEF, GLS) and LA function (LASr) and LAP (*E*/*e*ʹ ratio). Specifically, for LVEF and GLS, we found that 723 and 249 proteins, respectively, showed significant associations, after correcting for multiple testing (see Supplementary data online, *Table S1*). For LASr and *E*/*e*ʹ ratio, these were 792 and 427 proteins, respectively. The top 20 most strongly associated proteins for each of these echoparameters, and their effect estimates, are depicted in *Figure 1*. For LVEF, proteins previously associated with cardiovascular disease that were among the strongest associations included atrial natriuretic factor (ANP) and angiopoietin-2. NT-proBNP was among the 20 most strongly associated proteins for GLS. Associations with known cardiovascular-involved proteins were also found for LASr and *E*/*e*ʹ, including ANP and angiopoetin-2.

ANP, angiopoetin-2, MMP-2, SREC-II, CLD1, and Lumican were among the proteins that showed the strongest associations with both GLS and LVEF (LV function). For LASr and *E*/*e*ʹ (after adjustment for LVEF), NOTUM, ficolin-3, angiopoetin-2, ANP, and CRLD1 were overlapping in the top 20 proteins.

### Multivariable analysis of repeatedly measured circulating proteins in relation to repeatedly measured echoparameters

After running the multivariable LME models with LASSO, for LASr, we found that 16 proteins were significantly associated with LASr in a multivariable setting (*Table 3*). All these 16 proteins were significantly associated in the single-protein analyses with LASr as well, with HCC-1, IGFBP7, MFAP4, and MMP-2 among the 20 proteins with the strongest associations (*Table 3*). MMP-2, MFAP4, WFDC2, growth/differentiation factor 15 (GDF-15), and FGF-23 were present both among the proteins associated with elevated LAP and in the multivariable associations with LASr. After adjustment for LVEF, four proteins remained significantly and independently associated with LASr, namely MMP-2, Notch-3, vinculin, and IGFBP7.

**Table 3:** Results of multivariable analysis of circulating proteins in relation to LA strain

	Model 1	Model 2
Protein	Beta [change in % LASr per 1 SD change in log(protein level)]	
MMP-2	2.80	2.54
AT1B1	1.88	
HCC-1	1.56	
Notch-3	1.30	1.38
MFAP4	1.17	
Macrophage scavenger receptor	0.77	
Coagulation factor VII	0.68	
FGF-23	0.49	
Vinculin	0.10	0.69
HSPG2	−0.36	
IGF-I sR	−0.48	
HE4	−1.02	
Thymidine phosphorylase	−1.09	
IGFBP7	−2.04	−1.57
GDF-15	−2.85	
Growth hormone receptor	−4.48	

The presented betas depict the change in LASr when the protein level (explanatory variable) changes by 1 SD on the log scale. In model 1, we only adjusted for age and sex, and in model 2, we also added LVEF.

LASr, left atrial reservoir strain; GDF-15, growth/differentiation factor 15.

In the multivariable analyses for GLS, LVEF, and *E*/*e*ʹ, for each individual LASSO mixed model, there were also sets of proteins that showed significant associations with the outcome; however, when performing the 10-fold cross-validation, none of these proteins remained present in ≥8 of the folds. Therefore, results of these analyses are not reported.

### Gene enrichment analysis

**Figure 2:**
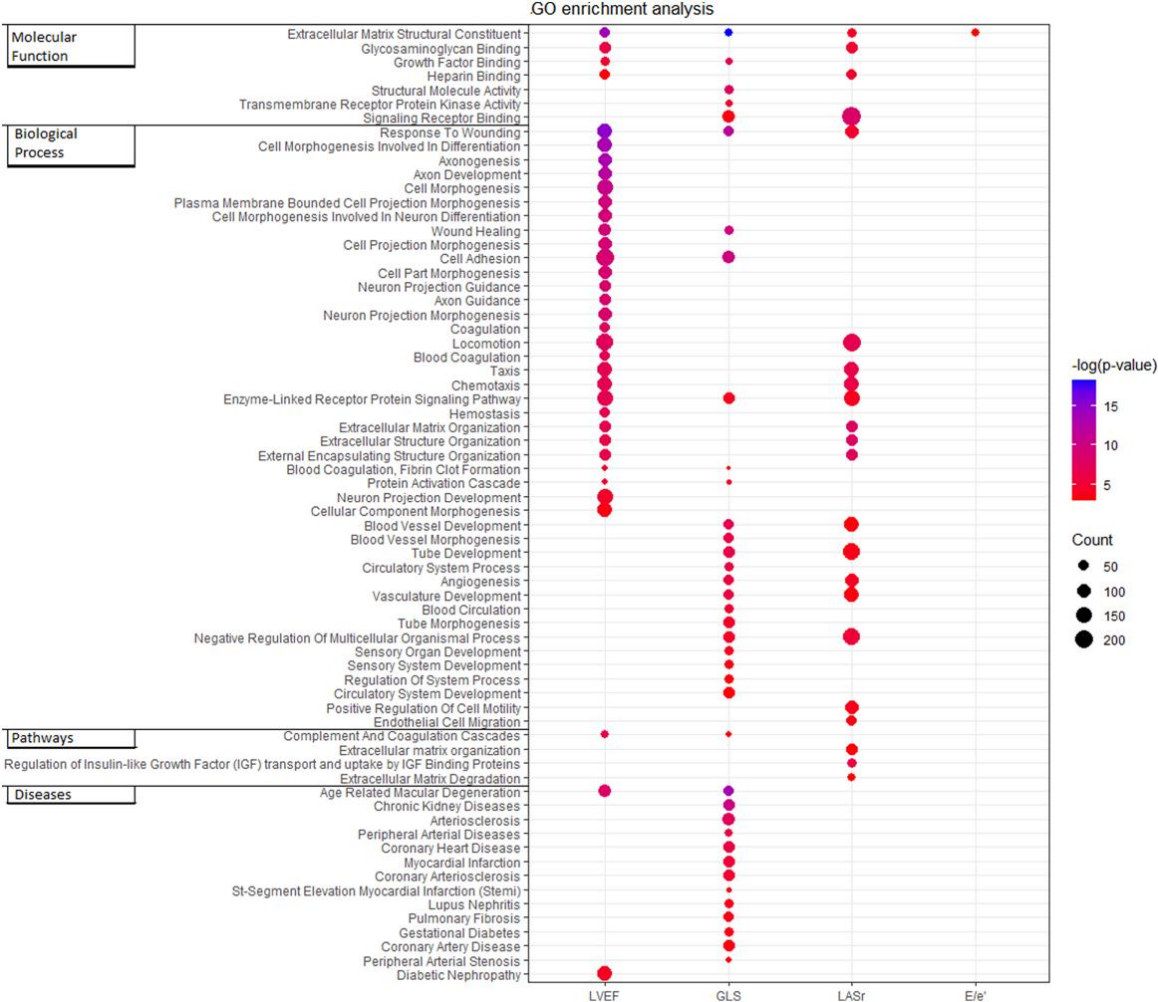
Gene enrichment analyses. All the circulating proteins that were significantly associated with echoparameters in the univariable analyses were further analysed using protein enrichment analysis (ToppGene). The colour of the dot represents the magnitude of the *P*-value. The size (‘Count’) represents how many of the included biomarkers are present in the mechanism or process.

A comprehensive overview of all biological processes related to each of the studied echoparameters, based on the associations found with the circulating proteins, is provided in *Figure 2*. Proteins associated with LVEF were proteins related to the sympathetic nervous system (SNS), such as axonogenesis, axon development, and cell morphogenesis involved in neurone differentiation. Proteins associated with GLS, on the other hand, were related to cardiovascular biological processes and diseases, such as vascular development and morphogenesis, myocardial infarction, heart disease, and atherosclerosis. The biological processes associated with LASr after adjustment for LVEF were mostly related to the extracellular matrix (ECM). Proteins associated with *E*/*e*ʹ also reflected the ECM, but after additionally adjusting for LVEF (to account for potential differences in systolic function over the *E*/*e*ʹ spectrum), statistically significant results were no longer present for the proteins that remained associated with *E*/*e*ʹ.

### Proteins associated with elevated LAP vs. normal LAP in patients with HFrEF

**Figure 3:**
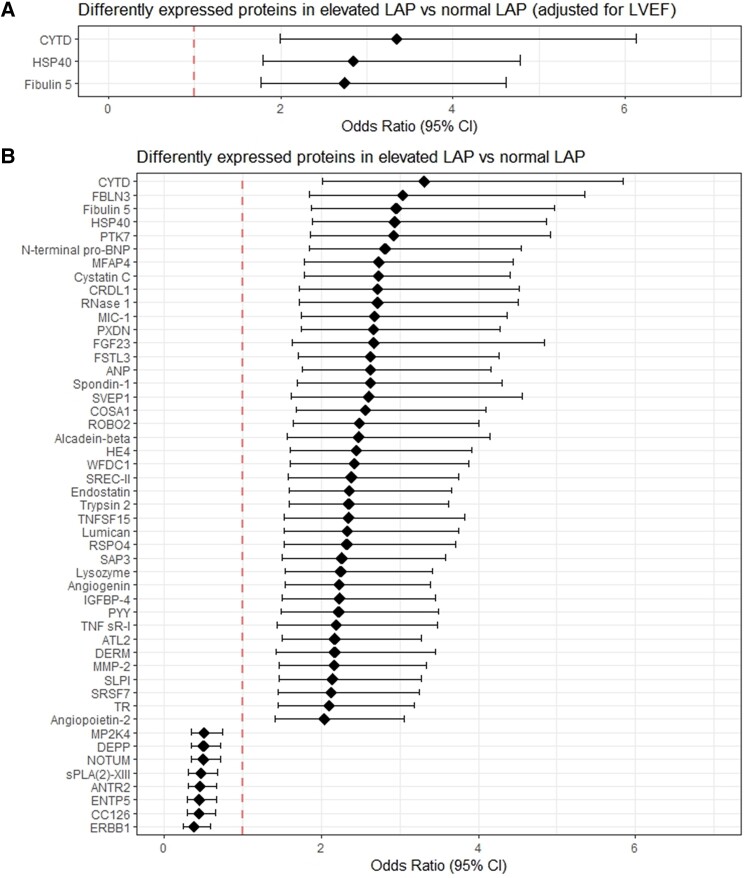
Proteins associated with elevated LAP vs. normal LAP. This figure represents the three proteins that were significantly associated with elevated LAP, after adjusting for LVEF (*A*) and the 49 proteins with a significant association with LAP without adjustment for LVEF (*B*).

We found 49 proteins associated with elevated LAP, after correction for age, sex, duration of and HF and after adjustment for multiple testing (*Figure 3* and Supplementary data online, *Table S2*). All 49 proteins were also statistically significantly associated with *E*/*e*ʹ ratio in single-protein analyses. The strongest associations were found for cystatin-D, epidermal growth factor (EGF)-containing fibulin-like ECM protein 1 (fibulin-3), and fibulin-5 [OR (95% CI): 3.31 (2.01–5.85), 3.04 (1.84–5.36), and 2.96 (1.87–4.97) per 1 SD difference in log-transformed protein levels, respectively]. NOTUM and EGF receptor were among the proteins inversely associated with elevated LAP [OR (95% CI): 0.50 (0.33–0.72) and 0.38 (0.23–0.58), respectively] (*Figure 3* and Supplementary data online, *Figure S3*). After additional adjustment for LVEF, three proteins remained significantly associated with elevated LAP, namely cystatin-D, fibulin-5, and HSP40 (*Figure 3*).

## Discussion

Our study in patients with HFrEF showed that (i) there are significant associations between repeatedly measured levels of a broad range of circulating proteins and repeatedly measured echocardiographic parameters of LV and LA function; (ii) circulating proteins associated with GLS reflect cardiovascular biological processes and diseases, whereas those associated with LVEF were related to biological processes that may reflect processes of the SNS, and those associated with LASr to processes involving the ECM; (iii) 49 circulating proteins were associated with elevated LAP and mostly related to the ECM, inflammation, myocardial stretch, and apoptosis; and (iv) three proteins remained linked to elevated LAP after adjustment for LVEF, namely cystatin-D, fibulin-5, and HSP40.

To our knowledge, we are the first to examine the association between proteomics and echocardiographic parameters in patients with HFrEF. Previous studies have focused on the associations between circulating proteins and cardiovascular events.^19^ Nayor *et al*.^20^ examined circulating proteins in relation to echocardiographic traits in a population-based setting. In addition, Gui *et al*.^11^ built an eight-biomarker model for prognostication in HFrEF, which was the first study examining ∼5000 proteins in HFrEF. Overall, studies investigating a broad range of circulating proteins in HFrEF are limited in number, and none have examined the association between circulating proteins and echocardiographic parameters in patients with HFrEF.

### Biological processes overrepresented in patients with more decreased LA function

Circulating proteins associated with LASr mostly reflected processes related to the ECM. In HFrEF, changes in the composition and organization of the ECM can contribute to the progressive deterioration of cardiac function.^21^ Studies have shown that in HFrEF, there is an increase in the production of ECM proteins, such as collagen and fibronectin, which can contribute to fibrosis and stiffening of the heart.^22^ These biological alterations to the heart may also be reflected by increased levels of several ECM proteins that are released into the bloodstream.^23,24^

### Biological processes overrepresented in patients with more decreased LV fucntion

Circulating proteins reflecting several cardiovascular biological processes and diseases were overrepresented in patients with lower GLS, whereas in patients with lower LVEF, biological mechanisms that may reflect SNS activity were overrepresented. The related cardiovascular biological processes (e.g. vascular development and morphogenesis) and diseases may reflect the initial myocardial injury and associated early pathophysiological changes in HF,^3,25^ some of which may also be reflected by increased circulating protein levels.^23,24^ It has previously been demonstrated that subtle changes in myocardial contractility can be revealed by GLS assessment.^3^ Furthermore, since GLS may already be impaired while LVEF is still normal,^25^ in our population of HF patients with reduced LVEF it may be expected that certain processes were overrepresented in patients with lower GLS.

Significant myocardial damage, as reflected by decreased LVEF (unlike the subtle changes reflected by GLS), may eventually lead to activation of certain systems, such as the SNS.^26,27^ After the activation of the SNS, a neurohormonal response is initiated. This response initially supports the heart but eventually accelerates the progression of HF, due to ventricular remodelling^26,27^ and increase of preload and afterload.^26^

### Circulating proteins associated with elevated LAP in patients with HFrEF

We found several circulating proteins known to be related to HF among the 49 proteins associated with elevated LAP, including NT-proBNP, ANP, cystatin-C, GDF-15, and EGF receptor (full list provided in *Figure 3*). Apart from NT-proBNP and ANP, most of these proteins do not originate from the heart. The proteins reflect neurohormonal activation, kidney dysfunction, ECM remodelling, myocardial stretch, and inflammation, all key pathways involved in the development and deterioration of HF.^28^ The strongest associations with elevated LAP were found for cystatin-D, EGF-containing fibulin-like ECM protein 1 (fibulin-3), and fibulin-5. Cystatin-D belongs to the family of cysteine proteinase inhibitors.^29^ Cysteine proteases have been implicated in the pathological remodelling of the heart that occurs in response to stress or injury, contributing to the development of fibrosis, hypertrophy, and apoptosis in the heart.^30^ Our study is the first reporting on the role of cystatin-D in HF. Fibulin-3 and fibulin-5 belong to the fibulin family of ECM proteins and are associated with changes in the mechanical composition of both large vessels and the heart, but their exact role still warrants further investigation.^31^ Cystatin-D and fibulin-5 remained significantly related to LAP after adjustment for LVEF, in addition to HSP40. HSP40 belongs to the heart-shock proteins that facilitate the removal of misfolded and degraded proteins (which inhibit apoptosis).^32^ These results are promising for identification of patients with elevated LAP by using blood biomarkers, which may carry potential for increasing efficiency of care.

### Limitations

Several limitations of our study warrant consideration. First, the patients in this echo substudy were relatively young and there were a high proportion of patients in NYHA classes I and II. Older patients with worse condition may have been less likely to participate in the substudy. However, prognosis of patients in advanced stages of HF is already known to be poor, whereas less is known about prognosis in the earlier stages. This supports the need for more insight in the pathophysiological mechanisms at less advanced stages. Second, the SOMAmer reagent targets the native folded protein. Therefore, unfolded and denatured proteins are not detected. Additionally, the SOMAscan assay provides RFU, not absolute concentrations. These values can be used to compare patients and longitudinal changes within each patient. However, in clinical practice, absolute concentrations are recommended for decision-making.

## Conclusion

Our study showed that circulating proteins associated with LASr reflected pathophysiological mechanisms mostly related to the ECM. GLS was associated with cardiovascular biological processes and diseases, whereas LVEF was associated with biological processes that may reflect processes of the SNS. These findings support the notion that GLS may be able to detect earlier physiological and morphological changes that occur in the myocardium, compared with LVEF. Furthermore, 49 circulating proteins were associated with elevated LAP in patients with HFrEF. These proteins were mostly related to the ECM, inflammation, myocardial stretch, and apoptosis. After adjustment for LVEF, three proteins remained significantly related to LAP, namely cystatin-D, fibulin-5, and HSP40. Further studies are needed to confirm these findings and to elaborate on the biological pathways implicated here.

## Supplementary data

Supplementary data are available at *European Heart Journal - Cardiovascular Imaging* online.

## Supplementary Material

jeae098_Supplementary_Data

## Data Availability

The data underlying this article will be shared on reasonable request to the corresponding author.

## References

[1] Roger VL . Epidemiology of heart failure: a contemporary perspective. Circ Res 2021;128:1421–34. 33983838 10.1161/CIRCRESAHA.121.318172

[2] Authors/Task Force Members; McDonagh TA, Metra M, Adamo M, Gardner RS, Baumbach A et al 2021 ESC guidelines for the diagnosis and treatment of acute and chronic heart failure: developed by the Task Force for the diagnosis and treatment of acute and chronic heart failure of the European Society of Cardiology (ESC). With the special contribution of the Heart Failure Association (HFA) of the ESC. Eur J Heart Fail 2022;24:4–131. 35083827 10.1002/ejhf.2333

[3] Patel J , Rikhi R, Hussain M, Ayoub C, Klein A, Collier P et al Global longitudinal strain is a better metric than left ventricular ejection fraction: lessons learned from cancer therapeutic-related cardiac dysfunction. Curr Opin Cardiol 2020;35:170–7. 31850935 10.1097/HCO.0000000000000716

[4] Nagueh SF , Smiseth OA, Appleton CP, Byrd BF III, Dokainish H, Edvardsen T et al Recommendations for the evaluation of left ventricular diastolic function by echocardiography: an update from the American Society of Echocardiography and the European Association of Cardiovascular Imaging. Eur Heart J Cardiovasc Imaging 2016;17:1321–60. 27422899 10.1093/ehjci/jew082

[5] Jin X , Nauta JF, Hung CL, Ouwerkerk W, Teng TK, Voors AA et al Left atrial structure and function in heart failure with reduced (HFrEF) versus preserved ejection fraction (HFpEF): systematic review and meta-analysis. Heart Fail Rev 2022;27:1933–55. 35079942 10.1007/s10741-021-10204-8 PMC9388424

[6] Abou Kamar S , Aga YS, de Bakker M, van den Berg VJ, Strachinaru M, Bowen D et al Prognostic value of temporal patterns of global longitudinal strain in patients with chronic heart failure. Front Cardiovasc Med 2023;9:1087596. 36712255 10.3389/fcvm.2022.1087596 PMC9878393

[7] Abou Kamar S , Aga YS, de Bakker M, van den Berg VJ, Strachinaru M, Bowen D et al Prognostic value of temporal patterns of left atrial reservoir strain in patients with heart failure with reduced ejection fraction. Clin Res Cardiol 2023:1–11. 10.1007/s00392-023-02244-x PMC11371887 37311973

[8] van den Berg VJ , Strachinaru M, Akkerhuis KM, Baart S, Brankovic M, Constantinescu AA et al Repeated echocardiograms do not provide incremental prognostic value to single echocardiographic assessment in minimally symptomatic patients with chronic heart failure: results of the Bio-SHiFT study. J Am Soc Echocardiogr 2019;32:1000–9. 31230778 10.1016/j.echo.2019.04.419

[9] Johnson FL . Pathophysiology and etiology of heart failure. Cardiol Clin 2014;32:9–19. vii. 24286575 10.1016/j.ccl.2013.09.015

[10] Smith JG , Gerszten RE. Emerging affinity-based proteomic technologies for large-scale plasma profiling in cardiovascular disease. Circulation 2017;135:1651–64. 28438806 10.1161/CIRCULATIONAHA.116.025446 PMC5555416

[11] Gui H , She R, Luzum J, Li J, Bryson TD, Pinto Y et al Plasma proteomic profile predicts survival in heart failure with reduced ejection fraction. Circ Genom Precis Med 2021;14:e003140. 33999650 10.1161/CIRCGEN.120.003140 PMC8221080

[12] van Boven N , Battes LC, Akkerhuis KM, Rizopoulos D, Caliskan K, Anroedh SS et al Toward personalized risk assessment in patients with chronic heart failure: detailed temporal patterns of NT-proBNP, troponin T, and CRP in the Bio-SHiFT study. Am Heart J 2018;196:36–48. 29421013 10.1016/j.ahj.2017.10.008

[13] Gold L , Ayers D, Bertino J, Bock C, Bock A, Brody EN et al Aptamer-based multiplexed proteomic technology for biomarker discovery. PLoS One 2010;5:e15004. 21165148 10.1371/journal.pone.0015004 PMC3000457

[14] Williams SA , Kivimaki M, Langenberg C, Hingorani AD, Casas JP, Bouchard C et al Plasma protein patterns as comprehensive indicators of health. Nat Med 2019;25:1851–7. 31792462 10.1038/s41591-019-0665-2 PMC6922049

[15] Lee CS , Lin TH, Hsu PC, Chu CY, Lee WH, Su HM et al Measuring left ventricular peak longitudinal systolic strain from a single beat in atrial fibrillation: validation of the index beat method. J Am Soc Echocardiogr 2012;25:945–52. 22763084 10.1016/j.echo.2012.06.006

[16] Subramanian A , Tamayo P, Mootha VK, Mukherjee S, Ebert BL, Gillette MA et al Gene set enrichment analysis: a knowledge-based approach for interpreting genome-wide expression profiles. Proc Natl Acad Sci U S A 2005;102:15545–50. 16199517 10.1073/pnas.0506580102 PMC1239896

[17] Schelldorfer J , Meier L, Bühlmann P. GLMMLasso: an algorithm for high-dimensional generalized linear mixed models using ℓ_1_-penalization. J Comput Graph Stat 2014;23:460–77.

[18] Pinheiro J , Bates D, R Core Team. nlme: linear and nonlinear mixed effects models (ed.), R Package version 3.1-164 2023. https://cran.r-project.org/package=nlme.

[19] Girerd N , Cleland J, Anker SD, Byra W, Lam CSP, Lapolice D et al Inflammation and remodeling pathways and risk of cardiovascular events in patients with ischemic heart failure and reduced ejection fraction. Sci Rep 2022;12:8574. 35595781 10.1038/s41598-022-12385-0 PMC9123183

[20] Nayor M , Short MI, Rasheed H, Lin H, Jonasson C, Yang Q et al Aptamer-based proteomic platform identifies novel protein predictors of incident heart failure and echocardiographic traits. Circ Heart Fail 2020;13:e006749. 32408813 10.1161/CIRCHEARTFAILURE.119.006749 PMC7236427

[21] Perestrelo AR , Silva AC, Oliver-De La Cruz J, Martino F, Horvath V, Caluori G et al Multiscale analysis of extracellular matrix remodeling in the failing heart. Circ Res 2021;128:24–38. 33106094 10.1161/CIRCRESAHA.120.317685

[22] Frangogiannis NG . The extracellular matrix in ischemic and nonischemic heart failure. Circ Res 2019;125:117–46. 31219741 10.1161/CIRCRESAHA.119.311148 PMC6588179

[23] Valiente-Alandi I , Schafer AE, Blaxall BC. Extracellular matrix-mediated cellular communication in the heart. J Mol Cell Cardiol 2016;91:228–37. 26778458 10.1016/j.yjmcc.2016.01.011 PMC4767504

[24] Chalikias GK , Tziakas DN. Biomarkers of the extracellular matrix and of collagen fragments. Clin Chim Acta 2015;443:39–47. 25007952 10.1016/j.cca.2014.06.028

[25] Potter E , Marwick TH. Assessment of left ventricular function by echocardiography: the case for routinely adding global longitudinal strain to ejection fraction. JACC Cardiovasc Imaging 2018;11:260–74. 29413646 10.1016/j.jcmg.2017.11.017

[26] Triposkiadis F , Karayannis G, Giamouzis G, Skoularigis J, Louridas G, Butler J. The sympathetic nervous system in heart failure physiology, pathophysiology, and clinical implications. J Am Coll Cardiol 2009;54:1747–62. 19874988 10.1016/j.jacc.2009.05.015

[27] Jackson G , Gibbs CR, Davies MK, Lip GY. ABC of heart failure. Pathophysiology. BMJ 2000;320:167–70. 10634740 10.1136/bmj.320.7228.167 PMC1128747

[28] Allach Y , Brugts JJ. The role of serial cardiac biomarkers in prognostication and risk prediction of chronic heart failure: additional scientific insights with hemodynamic feedback. Expert Rev Cardiovasc Ther 2023;21:97–109. 36744389 10.1080/14779072.2023.2177635

[29] Alvarez-Fernandez M , Liang YH, Abrahamson M, Su XD. Crystal structure of human cystatin D, a cysteine peptidase inhibitor with restricted inhibition profile. J Biol Chem 2005;280:18221–8. 15728581 10.1074/jbc.M411914200

[30] Cheng XW , Shi GP, Kuzuya M, Sasaki T, Okumura K, Murohara T. Role for cysteine protease cathepsins in heart disease: focus on biology and mechanisms with clinical implication. Circulation 2012;125:1551–62. 22451605 10.1161/CIRCULATIONAHA.111.066712

[31] Cangemi C , Hansen ML, Argraves WS, Rasmussen LM. Fibulins and their role in cardiovascular biology and disease. Adv Clin Chem 2014;67:245–65. 25735864 10.1016/bs.acc.2014.09.008

[32] Ranek MJ , Stachowski MJ, Kirk JA, Willis MS. The role of heat shock proteins and co-chaperones in heart failure. Philos Trans R Soc Lond B Biol Sci 2018;373:20160530. 29203715 10.1098/rstb.2016.0530 PMC5717530

