# Supplementary material for: The plasma proteome is linked with left ventricular and left atrial function parameters in patients with chronic heart failure
Source: Eur Heart J Cardiovasc Imaging. 2024 Apr 10;25(9):1206–15. doi: 10.1093/ehjci/jeae098 (PMC11346355; doi:10.1093/ehjci/jeae098)
Supplement: jeae098_Supplementary_Data [file jeae098_supplementary_data.docx]

# Supplementary material 1: Proteomic measurements

Somalogic’s previously described standard processes for normalization, calibration, and quality control (QC) were followed.^1^ The normalization and calibration factors are deemed acceptable as follows: hybridization control, intraplate median signal normalization and plate scale factors are expected to be between 0.4 and 2.5; the distribution of QC sample ratios is expected to have 85% of individual SOMAmer reagents in the total array between 0.8 and 1.2. SOMAmers outside these ranges were not considered for the current study. Moreover, SOMAmers with non-human and/or not-validated targets were excluded from further analyses. When multiple SOMAmer versions were present, those with the highest binding affinity were used. Thus, 4210 out of the total 5284 modified aptamers were included in the current analyses. Individual sample quality was judged by comparing normalized median signal relative to the external reference standard, with the acceptable normalization scaling range being 0.4 - 2.5. Data from 1066 samples passed quality-control criteria.

References

^1^Candia, J., Daya, G.N., Tanaka, T. et al. Assessment of variability in the plasma 7k SomaScan proteomics assay. Sci Rep 12, 17147 (2022). <https://doi.org/10.1038/s41598-022-22116-0>

# Supplementary material 2: Echocardiography measurements and evaluation

Two-dimensional gray-scale harmonic images were obtained in the left lateral decubitus position. Standard apical four-, three-, and two-chamber views were recorded. A commercially available ultrasound system was used (iE33, Philips, Best, The Netherlands), equipped with a broadband (1-5 MHz) S5-1 transducer (frequency transmitted 1.7 MHz, received 3.4 MHz). Images were stored in the echo core lab of Erasmus MC. Using specialized software (2D Cardiac Performance Analysis version 4.5; TomTec Imaging Systems, Unterschleissheim, Germany), LVEF, end-diastolic and end-systolic LV diameter, and end-systolic left atrial diameter were measured. The vena cava inferior diameter, the tricuspid regurgitation velocity, and the function of the valves were also assessed. The diastolic parameters were evaluated using Philips Excellera version R4.1 (Philips Medical Systems, The Netherlands) or TomTec Imaging Systems. To assess diastolic function, the peak early filling velocity (E)/late filling velocity (A) ratio and the E/e’ ratio were calculated. For the e’, we used the mean of the lateral and medial e’ when available; however, if only one of the two was available, this value was used.^1^ E/e’ > 15 was used to dichotomize patients in those with or without increased LAP.

Strain analysis based on speckle tracking echocardiography was also performed using TomTec Imaging Systems. The images were analysed retrospectively by a single operator, who was blinded to other echocardiographic parameters and the patients’ characteristics, after completion of follow-up.

The GLS assessment of the left ventricle was performed in 18 LV segments on the standard apical four-, three-, and two-chamber views, where the endocardial border was traced manually at end systole.^2^ GLS results were interpreted as absolute values. In other words, a change of GLS from for example −18% to −15% will be reported as a decrease of GLS. For the measurement of LA strain, the apical 4-chamber view was used preferably for the analysis. LA endocardial borders were automatically traced using end-diastole as a reference. When tracking was suboptimal, fine-tuning was performed manually. LA strain was assessed according to the three phases of the LA cycle: LA reservoir strain (LASr) which starts at the end of ventricular diastole (mitral valve closure) and continues until mitral valve opening, LA conduit strain (LAScd) which occurs from the time of mitral valve opening through diastasis until the onset of LA contraction, and LA contractile strain (LASct) which occurs from the onset of LA contraction until the end of ventricular diastole (mitral valve closure).^3^ LASr was used for the analysis.

References

^1^ van Boven N, Battes LC, Akkerhuis KM, Rizopoulos D, Caliskan K, Anroedh SS, et al. Toward personalized risk assessment in patients with chronic heart failure: Detailed temporal patterns of NT-proBNP, troponin T, and CRP in the Bio-SHiFT study. Am Heart J. 2018;196:36-48.

^2^Abou Kamar S, Aga YS, de Bakker M, van den Berg VJ, Strachinaru M, Bowen D, et al. Prognostic value of temporal patterns of global longitudinal strain in patients with chronic heart failure. Frontiers in Cardiovascular Medicine. 2023;9.

^3^Smiseth OA, Baron T, Marino PN, Marwick TH, Flachskampf FA. Imaging of the left atrium: pathophysiology insights and clinical utility. Eur Heart J Cardiovasc Imaging. 2021;23(1):2-13.

# Supplementary material 3: Statistical analysis

Distributions of continuous variables were tested for normality using the Shapiro-Wilk test. Normally distributed continuous variables are presented as mean ± standard deviation (SD), and nonnormally distributed variables as median and 25th-75th percentile. Categorical variables are presented as numbers and percentages. Differences in baseline characteristics between patients in the different LAP groups were tested using ANOVA and the Kruskal-Wallis test, according to variable distributions, for continuous variables, and χ2-tests and Fisher’s exact tests, when appropriate, for categorical variables.

For further analyses, all protein levels were log-transformed and subsequently, the Z-score was calculated. The timing of echocardiography and blood sampling did not necessarily coincide (Supplementary figure 1). Therefore, linear mixed effect (LME) models were first fitted to describe the temporal evolvement of the measured circulating proteins, and the obtained LME models were then used to estimate the protein level at the moment of the echocardiography. Thereafter, based on the obtained model parameters, we generated ten databases with sampled fitted protein values to account for the uncertainty of the fitted values. Then, we fitted the models of interest (repeatedly measured echoparameter as dependent variable, protein as independent variable) on the 10 dataframes. Thereafter, we used the Rubin’s Rule (used for imputation) to pool the results. We adjusted for sex, age, and duration of HF and, in the analyses for LASr and E/e’ ratio, also for LVEF. We also corrected for multiple testing using the Benjamini-Hochberg method (FDR < 0.05).

To determine which proteins remain statistically significantly associated with the echoparameters in a multivariable setting, we used linear mixed models with least absolute shrinkage and selection operator (LASSO). LASSO accounts for overfitting and multicollinearity, by using a penalization term (lambda) during the regression. For the selection of the optimal lambda term, a 10-fold cross-validation of the model was performed. Since the folds for the 10-fold cross-validation are selected randomly, we repeated the LASSO regression and the 10-fold cross-validation ten times. The proteins that were present in >= 8 of the folds, were considered relevant. We adjusted for sex, age, and duration of HF and, in the analyses for LASr and E/e’ ratio, also for LVEF.

All the circulating proteins that were significantly associated with echoparameters in the single-protein analyses, were further analysed using gene protein enrichment analysis (GEA) in ToppGene, with all 4210 proteins being used as the background set.

ToppGene is a tool for interpreting gene expression data and focuses on gene sets, i.e. groups of genes that share common biological and molecular functions, and pathways. The sets of proteins showing significant associations in the above-described analyses were evaluated in the tool, with all 4210 proteins being used as the background set.

Differences in protein levels between patients with elevated LAP and normal LAP were analyzed using logistic regression, with the LAP groups as the dependent variable and the proteins as the independent variables. We adjusted the regression models for age, sex, and duration of HF. In a second step, we adjusted for LVEF to account for the role of systolic dysfunction in the elevated LAP group. We also corrected for multiple testing (using the Benjamini-Hochberg method (FDR < 0.05). We calculated odds ratios (ORs) with 95% confidence intervals (CIs) per 1 SD difference in the log-transformed protein levels and presented the proteins that showed associations with LAP after adjustment for multiple testing in forest plots.

All analyses were performed with R Statistical Software using packages glmmLasso and nlme. All tests were two-tailed, and P values < .05 were considered statistically significant.

# Supplementary figure 1: Study design


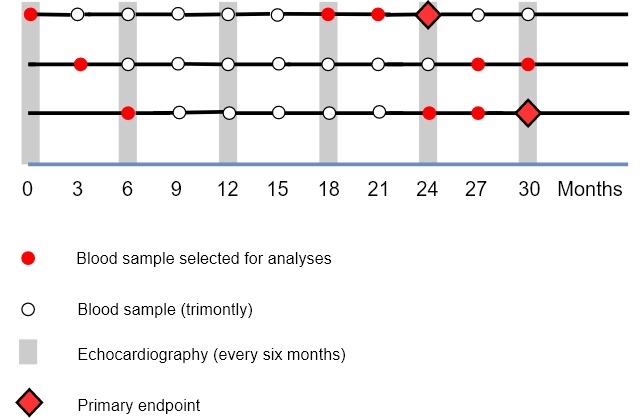


This figure provides 3 example patients to illustrate which echocardiograms were the first available echocardiograms and when the blood samples were drawn, and at which time-points follow-up echocardiograms and blood samples were scheduled. Echocardiograms were performed every six months (grey rectangles), whereas blood samples were drawn every three months (white circles). The blood samples at baseline and the two samples before the occurrence of the primary endpoint were used for the current analyses (red circles).

#
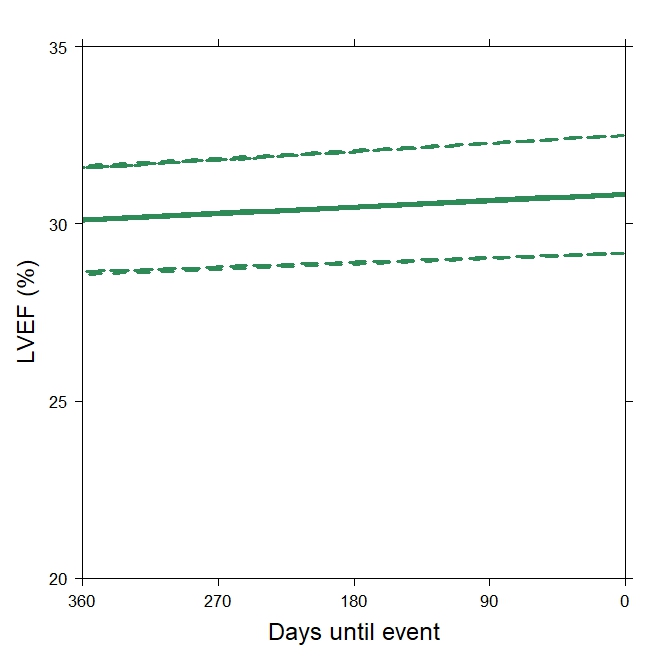
Supplementary figure 2: Temporal patterns echocardiographic parameters

Mean temporal patterns of LVEF, GLS, LASr and E/e’ ratio Dotted lines represent 95% confidence intervals. Each dot represents a single measurement.
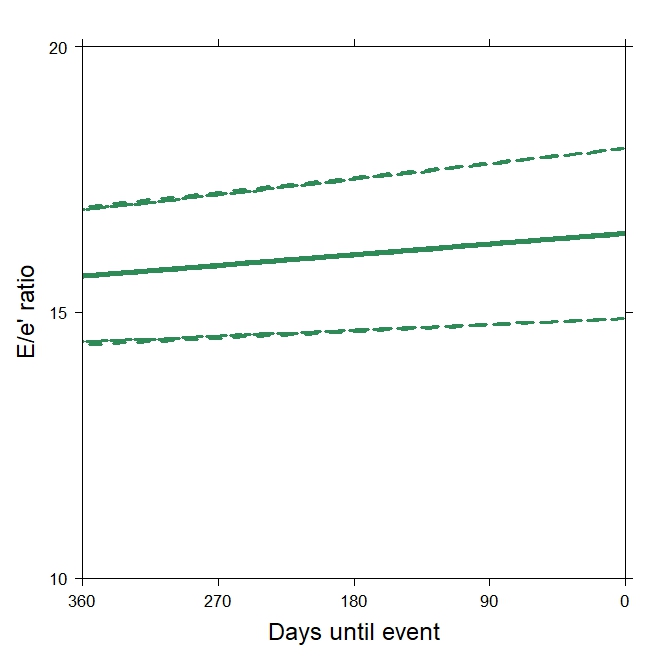

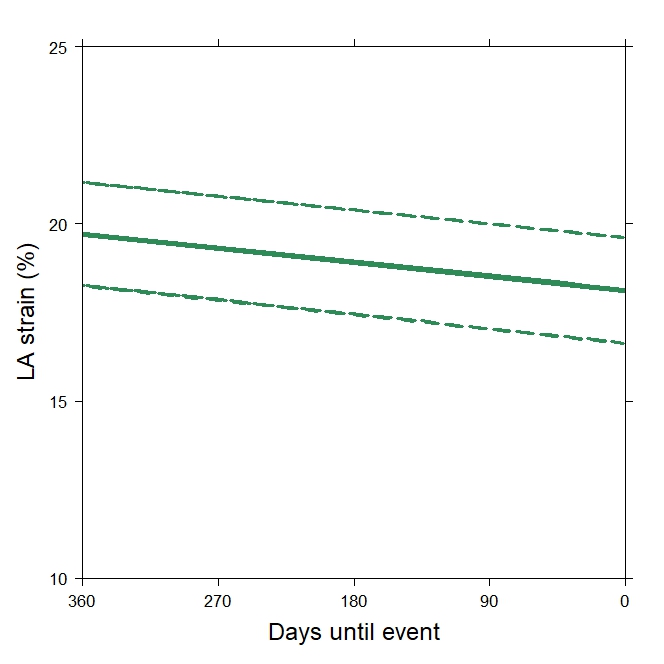

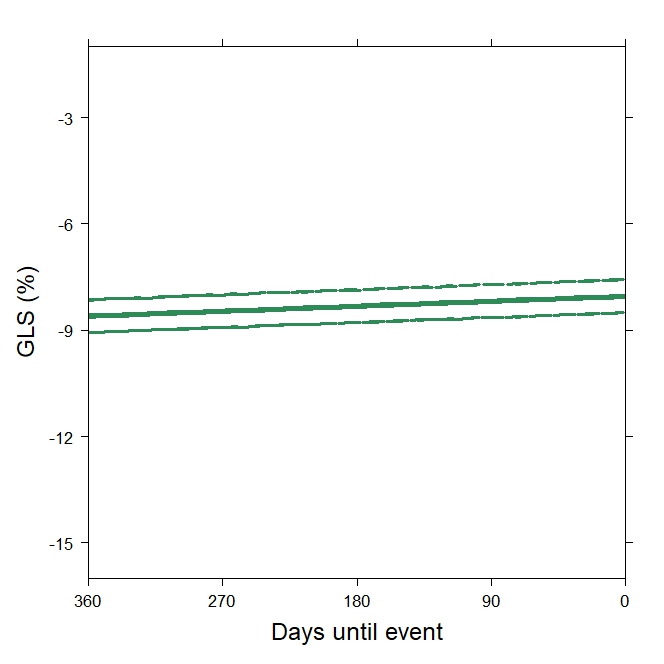


# Supplementary figure 3: Network analysis differently expressed proteins between patients with elevated LAP and normal LAP in HFrEF patients


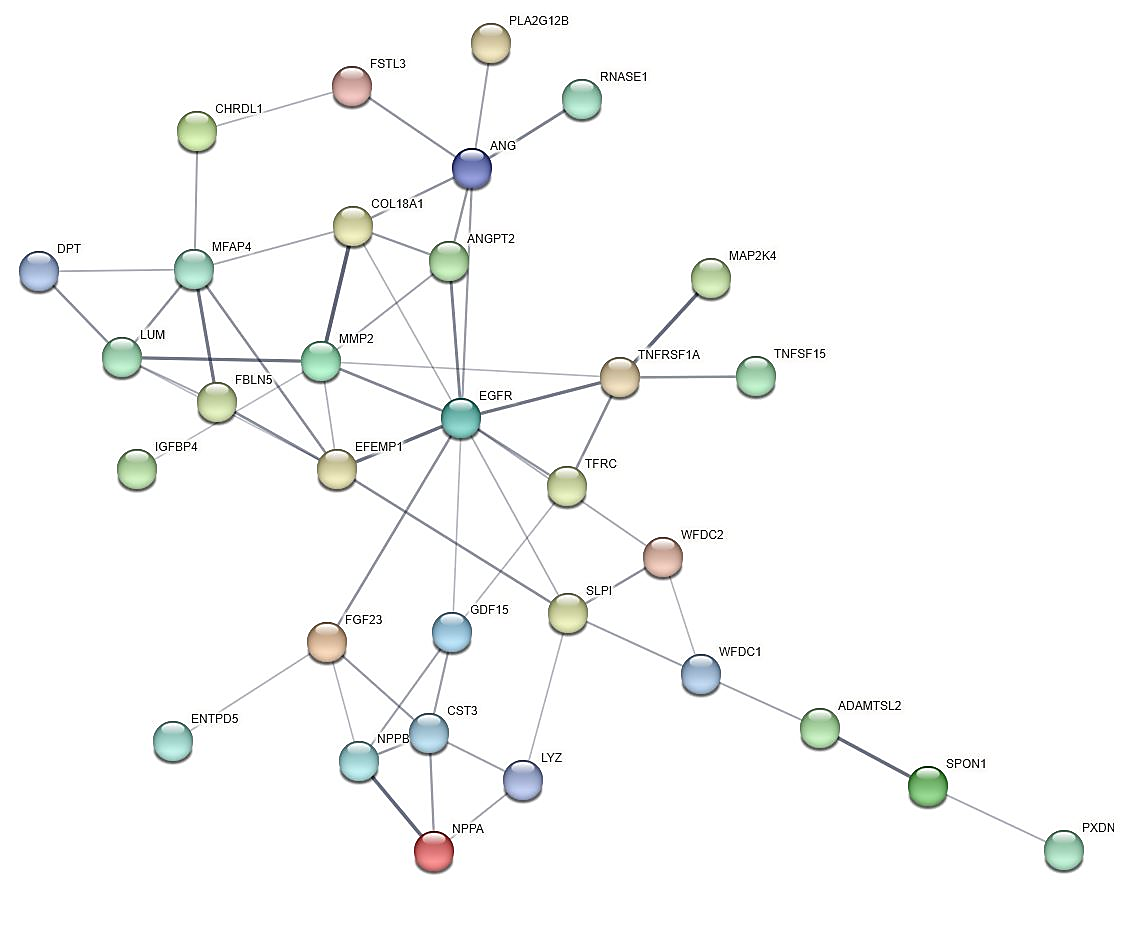


The thicker the line, the stronger the association between the biomarkers.

Supplementary table 1: Univariate associations with echocardiographic parameters

## Table 1.1 SysEF

| **Biomarker** | **Estimate** | **CI- Lower** | **CI- Upper** | **P-value** |
| --- | --- | --- | --- | --- |
| LUM.13114.50 | -4.73 | -6.13 | -3.33 | 0.0000 |
| ROBO2.5116.62 | -4.48 | -5.77 | -3.19 | 0.0000 |
| NPPA.5443.62 | -4.32 | -5.62 | -3.02 | 0.0000 |
| LRRC32.7551.33 | -4.16 | -5.52 | -2.79 | 0.0000 |
| CHRDL1.3362.61 | -4.49 | -6.10 | -2.89 | 0.0000 |
| CELA1.6107.3 | -4.05 | -5.40 | -2.71 | 0.0000 |
| SCARF2.8956.96 | -4.30 | -5.82 | -2.79 | 0.0000 |
| PTK7.9525.1 | -3.80 | -5.08 | -2.53 | 0.0000 |
| LAMA2.LAMB1.LAMC1.18347.15 | -3.87 | -5.19 | -2.56 | 0.0000 |
| NCAM2.6507.16 | -4.01 | -5.44 | -2.57 | 0.0000 |
| ROBO1.5740.17 | -3.88 | -5.26 | -2.50 | 0.0000 |
| MMP2.4160.49 | -3.94 | -5.36 | -2.52 | 0.0000 |
| PLXNA1.9005.16 | -3.91 | -5.35 | -2.48 | 0.0000 |
| CDH7.7959.34 | 3.76 | 2.41 | 5.10 | 0.0000 |
| ANGPT2.2602.2 | -3.60 | -4.86 | -2.34 | 0.0000 |
| FCN3.5462.62 | 3.78 | 2.34 | 5.22 | 0.0000 |
| F7.3184.25 | 3.63 | 2.30 | 4.97 | 0.0000 |
| FCN2.13717.15 | 3.68 | 2.26 | 5.10 | 0.0000 |
| ENG.4908.6 | -3.55 | -4.88 | -2.23 | 0.0000 |
| HSPG2.15626.223 | -3.42 | -4.68 | -2.17 | 0.0000 |
| POSTN.3457.57 | -3.66 | -5.07 | -2.25 | 0.0000 |
| ITLN1.18830.1 | -3.61 | -5.03 | -2.20 | 0.0000 |
| LTBP4.13133.73 | -3.59 | -4.98 | -2.19 | 0.0000 |
| TIMP2.2278.61 | -3.57 | -4.97 | -2.17 | 0.0000 |
| BCAM.2816.50 | -3.68 | -5.14 | -2.22 | 0.0000 |
| OMD.5358.3 | -3.40 | -4.70 | -2.10 | 0.0000 |
| NRP2.15387.44 | -3.38 | -4.71 | -2.05 | 0.0000 |
| ENTPD6.8932.1 | 3.57 | 2.11 | 5.03 | 0.0000 |
| FIGF.13098.93 | -3.53 | -4.99 | -2.08 | 0.0000 |
| FGFR1.5532.53 | -3.41 | -4.79 | -2.04 | 0.0000 |
| NPTN.7194.36 | -3.41 | -4.78 | -2.03 | 0.0000 |
| ITIH5.8233.2 | -3.57 | -5.04 | -2.09 | 0.0000 |
| FADD.16593.3 | 3.42 | 2.03 | 4.82 | 0.0000 |
| NLGN1.15620.4 | -3.42 | -4.86 | -1.99 | 0.0000 |
| APCS.2474.54 | 3.33 | 1.97 | 4.68 | 0.0000 |
| TMEM132D.13416.8 | -3.25 | -4.55 | -1.95 | 0.0000 |
| GCK.12960.9 | 3.39 | 1.97 | 4.81 | 0.0000 |
| NOTUM.8252.2 | 3.15 | 1.89 | 4.41 | 0.0000 |
| GAS1.5463.22 | -3.47 | -5.01 | -1.93 | 0.0000 |
| RSPO4.8464.31 | -3.58 | -5.21 | -1.95 | 0.0000 |
| CD93.14136.234 | -3.35 | -4.80 | -1.91 | 0.0000 |
| FMOD.6367.66 | -3.28 | -4.66 | -1.90 | 0.0000 |
| IGF1R.4232.19 | -3.26 | -4.63 | -1.89 | 0.0000 |
| SCARA5.10419.1 | -3.19 | -4.55 | -1.84 | 0.0000 |
| BMPER.15368.3 | -3.23 | -4.63 | -1.83 | 0.0000 |
| PRKG1.13067.5 | 3.37 | 1.85 | 4.88 | 0.0000 |
| NAALADL1.17505.125 | 3.42 | 1.84 | 5.01 | 0.0000 |
| OMG.16908.5 | -3.09 | -4.42 | -1.77 | 0.0000 |
| MRC2.3041.55 | -3.23 | -4.68 | -1.78 | 0.0000 |
| KERA.10758.2 | -3.06 | -4.37 | -1.75 | 0.0000 |
| ADIPOQ.3554.24 | -3.03 | -4.33 | -1.73 | 0.0000 |
| AMH.4923.79 | 3.11 | 1.70 | 4.51 | 0.0000 |
| CSF1R.13682.47 | -3.06 | -4.44 | -1.69 | 0.0000 |
| ADAM23.7049.2 | -3.13 | -4.60 | -1.65 | 0.0000 |
| ESM1.3805.16 | -3.02 | -4.41 | -1.64 | 0.0000 |
| SLC35G2.13501.10 | 3.08 | 1.62 | 4.53 | 0.0000 |
| MTAP.9910.9 | 3.04 | 1.61 | 4.47 | 0.0000 |
| C1QBP.4967.1 | 3.26 | 1.67 | 4.85 | 0.0000 |
| COL28A1.10702.1 | -3.17 | -4.71 | -1.62 | 0.0000 |
| COLEC12.5457.5 | -2.95 | -4.31 | -1.58 | 0.0000 |
| KLK10.6227.1 | -3.02 | -4.45 | -1.59 | 0.0000 |
| NAT1.12632.14 | 3.08 | 1.59 | 4.56 | 0.0000 |
| GPC6.5350.14 | 2.97 | 1.60 | 4.34 | 0.0000 |
| SFTPD.19590.46 | -3.03 | -4.46 | -1.61 | 0.0000 |
| C11orf87.11116.16 | 2.93 | 1.56 | 4.29 | 0.0000 |
| NOTCH3.5108.72 | -2.99 | -4.42 | -1.55 | 0.0000 |
| VWC2.15308.108 | -3.12 | -4.68 | -1.55 | 0.0000 |
| NMB.9321.400 | -3.09 | -4.65 | -1.54 | 0.0000 |
| PSMA5.18925.24 | 2.83 | 1.53 | 4.14 | 0.0000 |
| F13B.5658.64 | 2.79 | 1.52 | 4.05 | 0.0000 |
| ENTPD5.4437.56 | 2.95 | 1.53 | 4.37 | 0.0000 |
| CASQ1.11263.57 | 3.01 | 1.52 | 4.49 | 0.0000 |
| TFPI.3336.50 | 2.96 | 1.48 | 4.44 | 0.0000 |
| STAR.10085.25 | 3.02 | 1.49 | 4.55 | 0.0000 |
| LRP10.16610.13 | -2.94 | -4.44 | -1.45 | 0.0000 |
| CHST15.4469.78 | -2.83 | -4.24 | -1.42 | 0.0000 |
| FLRT2.13122.19 | -2.94 | -4.46 | -1.42 | 0.0000 |
| ATP1B2.7218.87 | -2.87 | -4.32 | -1.42 | 0.0000 |
| SPOCK2.5491.12 | -2.72 | -4.03 | -1.41 | 0.0000 |
| SURF1.8009.121 | 2.78 | 1.40 | 4.16 | 0.0000 |
| TEX29.10557.6 | 2.80 | 1.40 | 4.19 | 0.0000 |
| ZHX3.10036.201 | 2.69 | 1.39 | 4.00 | 0.0000 |
| NPFF.5617.41 | 2.87 | 1.40 | 4.35 | 0.0000 |
| IGFBP7.3320.49 | -2.67 | -3.96 | -1.38 | 0.0000 |
| TMEM132B.8890.9 | -2.77 | -4.16 | -1.39 | 0.0000 |
| HSPA13.17515.6 | -2.73 | -4.08 | -1.38 | 0.0000 |
| HERC5.12934.1 | 2.93 | 1.40 | 4.45 | 0.0000 |
| SUMF1.6941.11 | 2.69 | 1.38 | 4.01 | 0.0000 |
| SVEP1.11109.56 | -2.56 | -3.76 | -1.37 | 0.0000 |
| ENPP2.16892.23 | -2.73 | -4.08 | -1.37 | 0.0000 |
| FGF9.19584.33 | -2.68 | -3.99 | -1.36 | 0.0000 |
| ALDH5A1.17792.158 | 2.82 | 1.37 | 4.27 | 0.0000 |
| CDH3.2643.57 | 2.67 | 1.36 | 3.99 | 0.0000 |
| CAMK1.3592.4 | 2.86 | 1.36 | 4.36 | 0.0000 |
| HNF4A.10041.3 | 2.70 | 1.35 | 4.06 | 0.0000 |
| BMP4.15667.39 | -2.72 | -4.09 | -1.35 | 0.0000 |
| NRCAM.5109.24 | -2.75 | -4.16 | -1.33 | 0.0000 |
| NTF3.4145.58 | -2.74 | -4.14 | -1.33 | 0.0000 |
| MRPL21.8942.2 | 2.81 | 1.33 | 4.30 | 0.0000 |
| FGF23.3807.1 | -2.57 | -3.80 | -1.34 | 0.0000 |
| CLSTN2.18882.7 | -2.60 | -3.88 | -1.32 | 0.0000 |
| MLF1.17820.170 | 2.89 | 1.31 | 4.48 | 0.0000 |
| BOC.4328.2 | -2.70 | -4.09 | -1.30 | 0.0000 |
| GREM2.5598.3 | -2.79 | -4.26 | -1.32 | 0.0000 |
| PLAUR.2652.15 | -2.77 | -4.24 | -1.29 | 0.0000 |
| RBBP9.10064.12 | 2.62 | 1.28 | 3.95 | 0.0000 |
| ITIH1.7955.195 | 2.66 | 1.28 | 4.03 | 0.0000 |
| ISLR2.13124.20 | -2.67 | -4.06 | -1.28 | 0.0000 |
| ARL11.12433.8 | 2.88 | 1.29 | 4.46 | 0.0000 |
| SERPINA11.9002.36 | -2.65 | -4.04 | -1.26 | 0.0000 |
| JAG1.5092.51 | -2.75 | -4.24 | -1.26 | 0.0000 |
| KDR.3651.50 | 2.77 | 1.25 | 4.29 | 0.0000 |
| CNTN4.3298.52 | -2.58 | -3.91 | -1.25 | 0.0000 |
| ATF6B.11387.3 | 2.61 | 1.25 | 3.98 | 0.0000 |
| NPPB.7655.11 | -3.68 | -5.43 | -1.93 | 0.0000 |
| RBP7.14208.3 | -2.73 | -4.19 | -1.26 | 0.0000 |
| EFEMP1.8480.29 | -2.84 | -4.43 | -1.24 | 0.0000 |
| SELM.15336.7 | -2.92 | -4.64 | -1.20 | 0.0000 |
| NAP1L2.13529.39 | 2.69 | 1.20 | 4.17 | 0.0000 |
| SEMA6B.5121.3 | -2.60 | -4.00 | -1.20 | 0.0000 |
| SPON2.8099.42 | -2.81 | -4.44 | -1.19 | 0.0000 |
| WIF1.16070.7 | -2.73 | -4.27 | -1.18 | 0.0000 |
| CPB2.3518.54 | 2.59 | 1.18 | 4.01 | 0.0000 |
| DKK3.3607.71 | -2.65 | -4.14 | -1.17 | 0.0000 |
| CLIC2.17837.5 | -2.74 | -4.26 | -1.22 | 0.0000 |
| SEMA6A.7945.10 | -2.49 | -3.80 | -1.17 | 0.0000 |
| RNF8.14663.44 | 2.80 | 1.18 | 4.42 | 0.0000 |
| NMES1.6406.3 | 2.75 | 1.15 | 4.34 | 0.0000 |
| NRP1.5542.22 | -2.56 | -3.96 | -1.16 | 0.0000 |
| SERPIND1.3316.58 | 2.70 | 1.14 | 4.25 | 0.0000 |
| PTPRU.8337.65 | -2.64 | -4.11 | -1.16 | 0.0000 |
| FCGR3B.3311.27 | -2.53 | -3.92 | -1.15 | 0.0000 |
| TAGLN.15640.54 | -2.87 | -4.62 | -1.12 | 0.0000 |
| SH2D3C.12704.26 | 2.66 | 1.13 | 4.20 | 0.0000 |
| LRRK2.10990.21 | 2.59 | 1.13 | 4.05 | 0.0000 |
| NR1H2.9016.12 | 2.76 | 1.11 | 4.41 | 0.0000 |
| GRB14.13628.58 | 2.77 | 1.12 | 4.42 | 0.0000 |
| COPS2.14029.42 | 2.64 | 1.11 | 4.16 | 0.0000 |
| EGFLAM.12338.27 | -2.56 | -3.99 | -1.12 | 0.0000 |
| ZP4.7766.25 | 2.87 | 1.13 | 4.62 | 0.0000 |
| FREM2.7246.4 | 2.57 | 1.11 | 4.03 | 0.0000 |
| DTX1.11430.49 | 2.50 | 1.12 | 3.88 | 0.0000 |
| RBFOX2.11462.8 | -2.50 | -3.88 | -1.11 | 0.0000 |
| PACAP.16322.10 | -2.53 | -3.95 | -1.11 | 0.0000 |
| S100A16.17836.17 | -2.55 | -4.00 | -1.10 | 0.0000 |
| MFAP4.5636.10 | -2.54 | -3.95 | -1.12 | 0.0000 |
| CETN2.13078.3 | 2.59 | 1.09 | 4.08 | 0.0000 |
| UBE2G2.9199.6 | 2.47 | 1.10 | 3.84 | 0.0000 |
| F9.4876.32 | 2.60 | 1.08 | 4.12 | 0.0000 |
| PLA2R1.10916.44 | -2.58 | -4.08 | -1.07 | 0.0000 |
| FJX1.7921.65 | -2.44 | -3.79 | -1.09 | 0.0000 |
| MINPP1.5586.66 | -2.48 | -3.89 | -1.08 | 0.0000 |
| CHL1.8958.51 | -2.46 | -3.86 | -1.06 | 0.0000 |
| ARFGAP1.11556.19 | 2.61 | 1.05 | 4.17 | 0.0000 |
| NEO1.8900.28 | -2.44 | -3.82 | -1.06 | 0.0000 |
| PIANP.9599.6 | -2.55 | -4.06 | -1.03 | 0.0000 |
| CPLX2.15321.8 | -2.55 | -4.07 | -1.04 | 0.0000 |
| FBLN1.6470.19 | -2.50 | -3.98 | -1.03 | 0.0000 |
| FUT10.7156.2 | -2.54 | -4.07 | -1.02 | 0.0000 |
| WFDC2.11388.75 | -2.61 | -4.21 | -1.00 | 0.0000 |
| KLB.19557.3 | 2.42 | 1.03 | 3.82 | 0.0000 |
| BCL2A1.3413.50 | 2.60 | 1.01 | 4.20 | 0.0000 |
| COX6C.8903.1 | 2.31 | 1.04 | 3.59 | 0.0000 |
| VAV3.9830.109 | 2.52 | 1.02 | 4.03 | 0.0000 |
| MSR1.15533.97 | -2.68 | -4.36 | -1.00 | 0.0000 |
| ROR2.7861.9 | -2.56 | -4.12 | -0.99 | 0.0000 |
| PAM.5620.13 | -2.41 | -3.81 | -1.02 | 0.0000 |
| CXADR.11204.80 | 2.47 | 1.00 | 3.94 | 0.0000 |
| NAB1.13933.276 | 2.49 | 0.99 | 3.99 | 0.0000 |
| ACVRL1.16318.12 | -2.58 | -4.20 | -0.97 | 0.0000 |
| PCSK9.5231.79 | 2.35 | 1.01 | 3.70 | 0.0000 |
| CRLF1.14747.9 | -2.42 | -3.86 | -0.99 | 0.0000 |
| KLKB1.4152.58 | 2.45 | 0.98 | 3.92 | 0.0000 |
| MGAT2.6909.40 | 2.43 | 0.98 | 3.87 | 0.0000 |
| SSR1.8106.15 | 2.47 | 0.97 | 3.97 | 0.0000 |
| GHR.2948.58 | 2.49 | 0.98 | 4.00 | 0.0000 |
| IL19.3035.80 | -2.34 | -3.69 | -0.99 | 0.0000 |
| TLR4.LY96.3647.49 | -2.46 | -3.95 | -0.97 | 0.0000 |
| TREM1.9266.1 | -2.55 | -4.15 | -0.94 | 0.0000 |
| TREML2.5736.1 | 2.41 | 0.97 | 3.86 | 0.0000 |
| UNC5B.15394.79 | -2.54 | -4.15 | -0.93 | 0.0000 |
| STC1.4930.21 | -2.36 | -3.77 | -0.96 | 0.0000 |
| COL6A2.16753.46 | -2.34 | -3.71 | -0.96 | 0.0000 |
| TRDMT1.12635.9 | 2.36 | 0.94 | 3.79 | 0.0000 |
| KREMEN1.17331.138 | -2.34 | -3.74 | -0.94 | 0.0000 |
| SORCS1.15636.49 | -2.34 | -3.74 | -0.93 | 0.0000 |
| TRABD.11262.39 | 2.56 | 0.89 | 4.23 | 0.0000 |
| SPARCL1.4467.49 | -2.30 | -3.68 | -0.92 | 0.0000 |
| PPP1R2.19152.4 | 2.40 | 0.90 | 3.91 | 0.0000 |
| PTK2B.8918.64 | 2.41 | 0.89 | 3.93 | 0.0000 |
| SRSF7.12987.12 | -2.36 | -3.82 | -0.90 | 0.0000 |
| SCGB2A1.5001.6 | 2.40 | 0.88 | 3.92 | 0.0000 |
| NTRK3.2658.27 | -2.33 | -3.77 | -0.90 | 0.0000 |
| ALCAM.5451.1 | -2.30 | -3.69 | -0.90 | 0.0000 |
| DIXDC1.13441.30 | 2.32 | 0.90 | 3.74 | 0.0000 |
| CCDC126.6388.21 | 2.27 | 0.90 | 3.63 | 0.0000 |
| CST3.2609.59 | -2.42 | -3.98 | -0.85 | 0.0000 |
| PLTP.15475.4 | -2.32 | -3.75 | -0.88 | 0.0000 |
| PXDN.13463.1 | -2.34 | -3.80 | -0.87 | 0.0000 |
| ASCC1.10647.18 | 2.46 | 0.85 | 4.06 | 0.0000 |
| RUFY1.11425.31 | 2.33 | 0.88 | 3.78 | 0.0000 |
| IBSP.3415.61 | -2.20 | -3.51 | -0.89 | 0.0000 |
| TCEAL8.19109.32 | 2.30 | 0.87 | 3.73 | 0.0000 |
| HFE2.3332.57 | 2.23 | 0.88 | 3.58 | 0.0000 |
| AGER.4125.52 | -2.32 | -3.77 | -0.88 | 0.0000 |
| GDF15.4374.45 | -2.34 | -3.84 | -0.85 | 0.0000 |
| JTB.9038.12 | -2.31 | -3.77 | -0.85 | 0.0000 |
| PLA2G12B.9380.2 | 2.24 | 0.86 | 3.62 | 0.0000 |
| RHOG.12540.25 | 2.34 | 0.84 | 3.85 | 0.0000 |
| PDYN.19638.9 | 2.28 | 0.85 | 3.71 | 0.0000 |
| NDC80.12730.3 | 2.38 | 0.83 | 3.93 | 0.0000 |
| DEAF1.6369.82 | 2.47 | 0.82 | 4.11 | 0.0000 |
| F10.3077.66 | 2.31 | 0.83 | 3.79 | 0.0000 |
| FABP2.15385.116 | -2.28 | -3.73 | -0.83 | 0.0000 |
| OLFML3.8660.5 | -2.29 | -3.75 | -0.82 | 0.0000 |
| ATP1B1.13392.13 | 2.18 | 0.84 | 3.51 | 0.0000 |
| EFNA5.2615.60 | -2.37 | -3.95 | -0.78 | 0.0000 |
| CXCL13.3487.32 | -2.11 | -3.38 | -0.84 | 0.0000 |
| KL.15384.15 | -2.25 | -3.71 | -0.80 | 0.0000 |
| MYL7.19296.51 | 2.30 | 0.78 | 3.82 | 0.0000 |
| APLN.6622.90 | 2.30 | 0.79 | 3.81 | 0.0000 |
| RNF122.11160.56 | 2.33 | 0.77 | 3.88 | 0.0000 |
| ITIH2.9326.33 | 2.25 | 0.78 | 3.72 | 0.0000 |
| CDH5.2819.23 | -2.21 | -3.64 | -0.78 | 0.0000 |
| PKN1.12562.1 | 2.25 | 0.77 | 3.74 | 0.0000 |
| THBS3.8982.65 | -2.24 | -3.72 | -0.77 | 0.0000 |
| GDF2.4880.21 | -2.20 | -3.61 | -0.78 | 0.0000 |
| FGB.18890.227 | 2.29 | 0.74 | 3.84 | 0.0000 |
| RNASE6.5646.20 | -2.21 | -3.67 | -0.76 | 0.0000 |
| ADAMTS4.2809.25 | 2.35 | 0.73 | 3.96 | 0.0000 |
| SH3BP2.7769.29 | 2.17 | 0.77 | 3.57 | 0.0000 |
| CCDC80.3234.23 | -2.36 | -4.01 | -0.71 | 0.0000 |
| SLITRK5.4568.17 | -2.23 | -3.73 | -0.74 | 0.0000 |
| PRSS22.4534.10 | -2.15 | -3.53 | -0.77 | 0.0000 |
| GPNMB.8289.8 | -2.19 | -3.62 | -0.75 | 0.0000 |
| CYR61.6264.9 | -2.18 | -3.62 | -0.74 | 0.0000 |
| TPST2.8024.64 | 2.23 | 0.72 | 3.74 | 0.0000 |
| HS3ST5.10731.10 | 2.28 | 0.74 | 3.83 | 0.0000 |
| UMOD.9451.20 | -2.14 | -3.55 | -0.74 | 0.0000 |
| CHFR.11320.29 | 2.23 | 0.71 | 3.76 | 0.0000 |
| PENK.9076.25 | -2.24 | -3.77 | -0.70 | 0.0000 |
| ASB9.19601.15 | -2.38 | -4.13 | -0.63 | 0.0000 |
| WARS.9870.17 | -2.01 | -3.28 | -0.75 | 0.0000 |
| C1QTNF5.7810.20 | -2.09 | -3.46 | -0.73 | 0.0000 |
| CA9.3798.71 | -2.15 | -3.60 | -0.71 | 0.0000 |
| KIF3A.13961.18 | 2.21 | 0.69 | 3.73 | 0.0000 |
| PCDHGA10.6321.65 | -2.11 | -3.49 | -0.73 | 0.0000 |
| COL1A1.11140.56 | -2.15 | -3.60 | -0.69 | 0.0000 |
| GALNT16.8923.94 | -2.18 | -3.69 | -0.68 | 0.0000 |
| FGA.FGB.FGG.2796.62 | -2.16 | -3.64 | -0.68 | 0.0000 |
| SPARC.3043.49 | 2.24 | 0.65 | 3.83 | 0.0000 |
| CTSS.3181.50 | -2.23 | -3.82 | -0.64 | 0.0000 |
| TRA2B.12373.73 | -2.20 | -3.75 | -0.66 | 0.0000 |
| EFNA2.14124.6 | -2.23 | -3.83 | -0.64 | 0.0000 |
| IGFBP1.2771.35 | -2.10 | -3.51 | -0.68 | 0.0000 |
| WNT3A.13236.25 | 2.30 | 0.68 | 3.93 | 0.0000 |
| CHST11.7779.86 | -2.13 | -3.59 | -0.67 | 0.0000 |
| CES1.15487.164 | 2.17 | 0.66 | 3.68 | 0.0000 |
| CCL5.5480.49 | 2.22 | 0.63 | 3.80 | 0.0000 |
| STOML1.17344.23 | 2.09 | 0.68 | 3.51 | 0.0000 |
| IGSF3.9715.15 | -2.08 | -3.49 | -0.68 | 0.0000 |
| NPTXR.15511.37 | -2.10 | -3.54 | -0.66 | 0.0000 |
| HLA.DRB3.6962.5 | 2.14 | 0.65 | 3.63 | 0.0000 |
| IL26.16760.2 | 2.05 | 0.68 | 3.41 | 0.0000 |
| HSPA1B.18901.26 | -2.15 | -3.65 | -0.65 | 0.0000 |
| CTRB2.5648.28 | 2.08 | 0.66 | 3.50 | 0.0000 |
| EFS.12572.236 | 2.15 | 0.64 | 3.66 | 0.0000 |
| TPMT.11218.84 | 2.15 | 0.64 | 3.66 | 0.0000 |
| RGMA.5483.1 | 2.09 | 0.65 | 3.53 | 0.0000 |
| ARHGAP36.6289.78 | 2.07 | 0.66 | 3.48 | 0.0000 |
| MMP7.2789.26 | -2.17 | -3.72 | -0.62 | 0.0000 |
| CLCA2.8950.4 | 2.19 | 0.61 | 3.78 | 0.0000 |
| PRKCQ.3817.18 | 2.07 | 0.65 | 3.49 | 0.0000 |
| CLEC2D.7054.87 | 2.19 | 0.60 | 3.79 | 0.0000 |
| PPIB.4718.5 | -2.00 | -3.32 | -0.67 | 0.0000 |
| PLXND1.19561.216 | -2.18 | -3.75 | -0.60 | 0.0000 |
| FAM3B.9177.6 | -2.18 | -3.77 | -0.60 | 0.0000 |
| CD248.15491.20 | -2.16 | -3.72 | -0.61 | 0.0000 |
| OPTC.15430.165 | 2.14 | 0.61 | 3.66 | 0.0000 |
| PCSK2.6117.4 | -2.06 | -3.47 | -0.65 | 0.0000 |
| IL6ST.2620.4 | -2.08 | -3.54 | -0.63 | 0.0000 |
| TPPP2.12800.5 | -2.18 | -3.78 | -0.59 | 0.0000 |
| ECM1.3366.51 | -2.10 | -3.59 | -0.62 | 0.0000 |
| NBL1.2944.66 | -2.23 | -3.88 | -0.57 | 0.0000 |
| PCDHGC3.7859.21 | 2.15 | 0.60 | 3.70 | 0.0000 |
| CD72.7009.8 | -2.06 | -3.49 | -0.63 | 0.0000 |
| IL11RA.18216.22 | -2.15 | -3.71 | -0.60 | 0.0000 |
| ADAMTSL2.6379.62 | -1.93 | -3.19 | -0.67 | 0.0000 |
| PROS1.2700.56 | 2.10 | 0.61 | 3.60 | 0.0000 |
| PLEK.7875.86 | 2.09 | 0.61 | 3.56 | 0.0000 |
| PRCP.5722.78 | 2.00 | 0.64 | 3.36 | 0.0000 |
| FSTL1.13112.179 | -2.05 | -3.48 | -0.62 | 0.0000 |
| SERPINF1.9211.19 | 2.04 | 0.62 | 3.45 | 0.0000 |
| CREBBP.13614.6 | 2.07 | 0.61 | 3.54 | 0.0000 |
| TDO2.9880.33 | 2.07 | 0.61 | 3.53 | 0.0000 |
| IL1RL1.4234.8 | -1.95 | -3.25 | -0.65 | 0.0000 |
| HSPA8.5903.91 | -1.91 | -3.18 | -0.64 | 0.0000 |
| RTN4R.5105.2 | 2.02 | 0.60 | 3.44 | 0.0000 |
| MAP3K3.12990.39 | -2.04 | -3.49 | -0.60 | 0.0000 |
| COL11A2.11278.4 | -1.99 | -3.38 | -0.60 | 0.0000 |
| REG4.11102.22 | -2.23 | -3.96 | -0.50 | 0.0000 |
| C8G.14708.59 | 1.98 | 0.60 | 3.37 | 0.0000 |
| CD59.11514.196 | -2.10 | -3.67 | -0.53 | 0.0000 |
| TRIL.6527.1 | -1.99 | -3.40 | -0.57 | 0.0000 |
| FLRT3.13123.3 | -2.04 | -3.55 | -0.54 | 0.0000 |
| FURIN.6276.16 | -1.97 | -3.38 | -0.57 | 0.0000 |
| IL1B.3037.62 | -1.94 | -3.31 | -0.58 | 0.0000 |
| IMPDH2.5250.53 | 2.05 | 0.52 | 3.58 | 0.0000 |
| NPTX1.9256.78 | -1.97 | -3.39 | -0.55 | 0.0000 |
| CXCL10.4141.79 | -2.01 | -3.48 | -0.54 | 0.0000 |
| OSCAR.7116.31 | 2.08 | 0.49 | 3.68 | 0.0000 |
| HERC4.7860.9 | 2.05 | 0.50 | 3.60 | 0.0000 |
| TNFSF15.2968.61 | -2.11 | -3.74 | -0.47 | 0.0000 |
| PANK3.12658.72 | 2.16 | 0.46 | 3.85 | 0.0000 |
| TNFRSF11B.8304.50 | -2.09 | -3.70 | -0.48 | 0.0000 |
| CCL14.2900.53 | -1.94 | -3.35 | -0.54 | 0.0000 |
| SPIN1.18210.12 | -1.92 | -3.29 | -0.54 | 0.0000 |
| AIMP1.2714.78 | 1.89 | 0.55 | 3.23 | 0.0000 |
| IGFLR1.7244.16 | -2.09 | -3.72 | -0.46 | 0.0000 |
| DOK1.16831.7 | 1.91 | 0.54 | 3.28 | 0.0000 |
| PCDH9.10558.26 | -1.98 | -3.46 | -0.50 | 0.0000 |
| PSAP.6207.10 | -1.93 | -3.34 | -0.52 | 0.0000 |
| ARL6.18415.16 | 2.06 | 0.48 | 3.64 | 0.0000 |
| PRDX6.5018.68 | 2.05 | 0.46 | 3.64 | 0.0000 |
| APOA1BP.16621.77 | 2.00 | 0.48 | 3.52 | 0.0000 |
| ARL15.18411.83 | 1.88 | 0.53 | 3.23 | 0.0000 |
| SCGB1D2.6508.68 | 2.09 | 0.45 | 3.73 | 0.0000 |
| SRC.15433.4 | 2.03 | 0.46 | 3.61 | 0.0000 |
| DCTPP1.4314.12 | -1.99 | -3.50 | -0.48 | 0.0000 |
| PSME2.17694.32 | 1.95 | 0.49 | 3.41 | 0.0000 |
| CBR1.12381.26 | -1.99 | -3.52 | -0.47 | 0.0000 |
| CPZ.6493.9 | -1.87 | -3.22 | -0.52 | 0.0000 |
| KAAG1.19492.5 | 1.87 | 0.51 | 3.23 | 0.0000 |
| DNAJB12.8006.12 | -2.04 | -3.66 | -0.42 | 0.0000 |
| KRT5.11177.16 | 1.82 | 0.53 | 3.10 | 0.0000 |
| QPCTL.8866.53 | -1.94 | -3.43 | -0.46 | 0.0000 |
| EMC4.13516.46 | 1.93 | 0.47 | 3.40 | 0.0000 |
| GLTPD2.7948.129 | 1.92 | 0.47 | 3.37 | 0.0000 |
| SEPSECS.17357.33 | 1.87 | 0.50 | 3.25 | 0.0000 |
| NAPG.17773.26 | 1.96 | 0.45 | 3.47 | 0.0000 |
| HSP90AB1.5467.15 | 1.98 | 0.43 | 3.54 | 0.0000 |
| DCLK1.17156.72 | -1.93 | -3.41 | -0.45 | 0.0000 |
| CXCL6.3495.15 | 1.86 | 0.49 | 3.24 | 0.0000 |
| HRAS.18900.37 | 1.96 | 0.43 | 3.50 | 0.0000 |
| F13A1.F13B.16927.9 | 1.81 | 0.50 | 3.12 | 0.0000 |
| SULT1B1.12671.35 | 1.89 | 0.47 | 3.31 | 0.0000 |
| CHST9.11646.4 | -1.83 | -3.17 | -0.49 | 0.0000 |
| BGN.3284.75 | -1.95 | -3.48 | -0.43 | 0.0000 |
| SUMF2.6069.71 | -1.91 | -3.38 | -0.45 | 0.0000 |
| INHBB.13676.46 | -1.88 | -3.30 | -0.46 | 0.0000 |
| LY86.3623.84 | 2.08 | 0.37 | 3.78 | 0.0000 |
| C14orf93.6439.59 | 2.01 | 0.40 | 3.62 | 0.0000 |
| SPSB1.13942.140 | 1.95 | 0.41 | 3.49 | 0.0000 |
| BCHE.15514.26 | 1.85 | 0.46 | 3.23 | 0.0000 |
| RAD1.12670.15 | 1.87 | 0.45 | 3.28 | 0.0000 |
| TNNT2.5315.22 | -1.82 | -3.16 | -0.48 | 0.0000 |
| CD33.3166.92 | -1.91 | -3.41 | -0.41 | 0.0000 |
| KLK13.11152.46 | -1.91 | -3.40 | -0.42 | 0.0000 |
| ELF5.13457.33 | 2.02 | 0.35 | 3.68 | 0.0000 |
| IL3RA.13744.37 | -1.94 | -3.48 | -0.40 | 0.0000 |
| PPA2.18307.71 | 1.93 | 0.40 | 3.46 | 0.0000 |
| SKP1.3902.21 | 2.02 | 0.34 | 3.69 | 0.0000 |
| WISP2.6392.7 | -2.13 | -4.00 | -0.26 | 0.0000 |
| PTPN4.14254.27 | 1.87 | 0.42 | 3.32 | 0.0000 |
| BMP6.8459.10 | -1.81 | -3.18 | -0.45 | 0.0000 |
| TNXB.5698.60 | -1.88 | -3.37 | -0.39 | 0.0000 |
| FLT4.16035.8 | -1.84 | -3.28 | -0.41 | 0.0000 |
| IGFBP5.19581.15 | -1.78 | -3.12 | -0.44 | 0.0000 |
| BDNF.14047.78 | 1.88 | 0.38 | 3.39 | 0.0000 |
| NUDT9.9482.110 | 1.91 | 0.36 | 3.45 | 0.0000 |
| HAAO.5861.78 | 1.87 | 0.38 | 3.37 | 0.0000 |
| SNTA1.19274.80 | 1.87 | 0.38 | 3.36 | 0.0000 |
| CDCP1.16818.200 | -1.96 | -3.59 | -0.32 | 0.0001 |
| RGS19.12713.365 | 1.75 | 0.43 | 3.07 | 0.0001 |
| FAM171A2.13479.8 | 1.85 | 0.38 | 3.32 | 0.0001 |
| IMPDH1.5229.90 | 1.86 | 0.37 | 3.36 | 0.0001 |
| TEK.3773.15 | -1.89 | -3.42 | -0.35 | 0.0001 |
| PRDM4.12779.30 | 1.88 | 0.36 | 3.40 | 0.0001 |
| ADH6.18206.18 | 1.90 | 0.34 | 3.47 | 0.0001 |
| CXCL12.3516.60 | -1.85 | -3.33 | -0.37 | 0.0001 |
| PSME1.5918.5 | 1.87 | 0.36 | 3.38 | 0.0001 |
| LRRC15.6557.50 | -1.79 | -3.18 | -0.40 | 0.0001 |
| LILRA5.7787.25 | 1.85 | 0.37 | 3.33 | 0.0001 |
| CLIC5.12475.48 | -1.92 | -3.51 | -0.32 | 0.0001 |
| SYT4.17355.56 | 1.87 | 0.35 | 3.39 | 0.0001 |
| SELL.4831.4 | -1.83 | -3.28 | -0.37 | 0.0001 |
| CRIP2.9053.16 | -1.88 | -3.41 | -0.34 | 0.0001 |
| IL1R1.2991.9 | -1.81 | -3.24 | -0.38 | 0.0001 |
| ARPP19.4963.19 | 1.85 | 0.34 | 3.36 | 0.0001 |
| ADAMTSL1.16890.37 | -2.00 | -3.77 | -0.23 | 0.0001 |
| MLL2.13623.4 | 1.86 | 0.33 | 3.40 | 0.0001 |
| MOBKL3.19332.1 | 1.90 | 0.31 | 3.50 | 0.0001 |
| CAND1.13937.75 | 1.88 | 0.32 | 3.43 | 0.0001 |
| SPP1.13113.7 | -1.73 | -3.07 | -0.40 | 0.0001 |
| TNFAIP8.12563.2 | 1.83 | 0.34 | 3.33 | 0.0001 |
| PAPSS1.14007.22 | 1.81 | 0.35 | 3.27 | 0.0001 |
| TMEM132A.7871.16 | -1.75 | -3.11 | -0.38 | 0.0001 |
| SPOCK1.5490.53 | 1.86 | 0.31 | 3.40 | 0.0001 |
| F8.13499.30 | -1.87 | -3.43 | -0.30 | 0.0001 |
| EDAR.2977.7 | 1.77 | 0.37 | 3.17 | 0.0001 |
| AGT.3484.60 | 1.72 | 0.39 | 3.05 | 0.0001 |
| SLC6A14.13053.6 | 1.79 | 0.34 | 3.25 | 0.0001 |
| DSC2.13126.52 | -1.79 | -3.24 | -0.34 | 0.0001 |
| ACTN1.9843.5 | 1.90 | 0.28 | 3.51 | 0.0001 |
| TPRKB.12417.46 | 1.74 | 0.37 | 3.10 | 0.0001 |
| APOL1.9506.10 | 1.73 | 0.38 | 3.08 | 0.0002 |
| KIR3DL1.18907.97 | 1.84 | 0.31 | 3.38 | 0.0002 |
| ADGRF1.11243.90 | 1.84 | 0.30 | 3.38 | 0.0002 |
| NDUFB11.7747.47 | 1.79 | 0.34 | 3.24 | 0.0002 |
| MATN3.19361.78 | -1.75 | -3.15 | -0.36 | 0.0002 |
| SIGLEC8.7864.3 | 1.72 | 0.37 | 3.06 | 0.0002 |
| YWHAB.14156.33 | 1.84 | 0.30 | 3.37 | 0.0002 |
| AFM.4763.31 | 1.76 | 0.35 | 3.17 | 0.0002 |
| TIMP1.2211.9 | -1.83 | -3.36 | -0.30 | 0.0002 |
| PDE4A.18918.86 | 1.73 | 0.36 | 3.10 | 0.0002 |
| AKR1A1.4192.10 | 1.84 | 0.29 | 3.38 | 0.0002 |
| IGHG4.13231.90 | 1.69 | 0.38 | 2.99 | 0.0002 |
| GEM.12817.1 | 1.78 | 0.32 | 3.23 | 0.0002 |
| NAMPT.5011.11 | -1.54 | -2.65 | -0.44 | 0.0002 |
| EGFR.2677.1 | 1.82 | 0.29 | 3.35 | 0.0002 |
| SPINK7.10974.20 | -1.82 | -3.36 | -0.28 | 0.0002 |
| NRXN1.8971.9 | 1.72 | 0.34 | 3.11 | 0.0002 |
| ACP5.3232.28 | 1.72 | 0.34 | 3.09 | 0.0002 |
| ASPN.6451.64 | -1.78 | -3.26 | -0.30 | 0.0003 |
| ZNF329.12803.9 | 1.81 | 0.27 | 3.34 | 0.0003 |
| S100A6.13090.17 | 1.88 | 0.22 | 3.54 | 0.0003 |
| IGLON5.6478.2 | -1.72 | -3.13 | -0.32 | 0.0003 |
| NQO1.9837.60 | -1.81 | -3.34 | -0.27 | 0.0003 |
| CENPW.8864.59 | 1.69 | 0.34 | 3.05 | 0.0003 |
| CHAD.13460.4 | -1.69 | -3.05 | -0.33 | 0.0003 |
| INA.11436.6 | -1.79 | -3.31 | -0.27 | 0.0003 |
| DLK1.6373.54 | 1.77 | 0.28 | 3.26 | 0.0003 |
| RPS6KB1.15608.5 | 1.78 | 0.27 | 3.29 | 0.0003 |
| PRSS35.9983.97 | 1.85 | 0.22 | 3.48 | 0.0004 |
| LEG1.7154.92 | 1.78 | 0.27 | 3.30 | 0.0004 |
| NTN4.3327.27 | -1.67 | -3.00 | -0.34 | 0.0004 |
| PPA1.5021.13 | 1.77 | 0.27 | 3.27 | 0.0004 |
| MENT.5744.12 | 1.76 | 0.28 | 3.23 | 0.0004 |
| APOA5.15363.32 | 1.72 | 0.30 | 3.14 | 0.0004 |
| RNGTT.12847.27 | 1.82 | 0.24 | 3.39 | 0.0004 |
| COL18A1.2201.17 | -1.79 | -3.32 | -0.26 | 0.0004 |
| ERP29.13728.19 | 1.88 | 0.21 | 3.54 | 0.0004 |
| NAGK.3894.15 | 1.80 | 0.25 | 3.34 | 0.0004 |
| COLGALT1.5638.23 | 1.80 | 0.25 | 3.35 | 0.0004 |
| HSP90AA1.2625.53 | 1.79 | 0.25 | 3.33 | 0.0004 |
| CALCOCO2.12534.10 | -1.72 | -3.15 | -0.30 | 0.0004 |
| CHMP2B.17350.13 | 1.70 | 0.30 | 3.10 | 0.0004 |
| FKBP6.12529.32 | 1.78 | 0.24 | 3.32 | 0.0005 |
| VIT.6234.74 | -1.84 | -3.48 | -0.20 | 0.0005 |
| SULT1A3.13944.3 | 1.75 | 0.26 | 3.24 | 0.0005 |
| NPTX2.6521.35 | 1.77 | 0.24 | 3.29 | 0.0006 |
| CNPY4.15465.79 | 1.76 | 0.24 | 3.28 | 0.0006 |
| QDPR.11257.1 | 1.79 | 0.23 | 3.34 | 0.0006 |
| C4BPA.9449.150 | 1.71 | 0.27 | 3.14 | 0.0006 |
| ANXA6.5335.73 | 1.73 | 0.25 | 3.20 | 0.0007 |
| MAPK14.5007.1 | 1.78 | 0.22 | 3.33 | 0.0007 |
| PLXDC2.11342.59 | 1.77 | 0.22 | 3.32 | 0.0007 |
| PLXNC1.4564.2 | -1.73 | -3.23 | -0.23 | 0.0008 |
| CRKL.9877.28 | 1.87 | 0.15 | 3.58 | 0.0008 |
| ST6GALNAC6.7228.2 | -1.76 | -3.31 | -0.21 | 0.0008 |
| AMY1A.7918.114 | -1.68 | -3.08 | -0.27 | 0.0008 |
| LRRC4C.9369.174 | -1.74 | -3.25 | -0.23 | 0.0008 |
| PDCD6IP.18174.79 | 1.83 | 0.17 | 3.49 | 0.0008 |
| CASP10.5340.24 | 1.75 | 0.22 | 3.28 | 0.0008 |
| IL17RA.2992.59 | -1.67 | -3.08 | -0.27 | 0.0008 |
| TFRC.6895.1 | -1.66 | -3.03 | -0.28 | 0.0009 |
| STIP1.5489.18 | 1.73 | 0.23 | 3.23 | 0.0009 |
| COTL1.4905.63 | 1.80 | 0.17 | 3.42 | 0.0009 |
| C1QTNF1.6304.8 | -1.64 | -3.01 | -0.28 | 0.0009 |
| CEACAM7.7184.13 | 1.68 | 0.25 | 3.11 | 0.0009 |
| ADAMTS3.8845.2 | 1.70 | 0.23 | 3.18 | 0.0010 |
| TXNDC5.11212.7 | 1.72 | 0.23 | 3.21 | 0.0010 |
| ASAH1.5748.20 | 1.71 | 0.22 | 3.20 | 0.0010 |
| WFDC1.9316.67 | -1.79 | -3.40 | -0.17 | 0.0010 |
| RAB6B.14271.23 | 1.72 | 0.22 | 3.22 | 0.0010 |
| APOA2.7127.3 | -1.68 | -3.11 | -0.24 | 0.0011 |
| KIAA0040.14603.51 | 1.68 | 0.24 | 3.11 | 0.0011 |
| KLK11.2831.29 | -1.74 | -3.29 | -0.20 | 0.0011 |
| ANGPTL4.3796.79 | -1.70 | -3.19 | -0.22 | 0.0011 |
| DHRS9.17467.1 | 1.71 | 0.21 | 3.20 | 0.0012 |
| F2.5316.54 | 1.69 | 0.21 | 3.17 | 0.0013 |
| GAA.9385.4 | -1.64 | -3.04 | -0.25 | 0.0013 |
| WFDC3.6384.19 | 1.72 | 0.19 | 3.26 | 0.0013 |
| CADM1.3326.58 | -1.71 | -3.24 | -0.19 | 0.0014 |
| FBXL5.12846.3 | 1.73 | 0.17 | 3.29 | 0.0014 |
| CHCHD10.11270.17 | -1.64 | -3.05 | -0.24 | 0.0014 |
| G6PD.19297.4 | 1.70 | 0.20 | 3.20 | 0.0014 |
| CFD.2946.52 | -1.75 | -3.34 | -0.16 | 0.0014 |
| GDF7.16756.30 | 1.62 | 0.25 | 2.99 | 0.0014 |
| HSPB6.19127.1 | -1.78 | -3.42 | -0.13 | 0.0014 |
| YWHAZ.5858.6 | 1.75 | 0.15 | 3.35 | 0.0015 |
| NSF.13992.12 | 1.77 | 0.14 | 3.40 | 0.0015 |
| BCL2L2.13097.11 | 1.70 | 0.19 | 3.21 | 0.0015 |
| CPXM1.6255.74 | 1.67 | 0.21 | 3.12 | 0.0015 |
| RSPO1.16614.27 | -1.80 | -3.48 | -0.12 | 0.0015 |
| PTGDS.10514.5 | -1.76 | -3.39 | -0.13 | 0.0016 |
| ALDH1A3.9835.16 | 1.68 | 0.20 | 3.16 | 0.0016 |
| CSH1.CSH2.13103.125 | 1.75 | 0.15 | 3.34 | 0.0017 |
| SEMA3E.5363.51 | -1.66 | -3.11 | -0.21 | 0.0017 |
| MAPK10.15418.25 | 1.73 | 0.15 | 3.31 | 0.0018 |
| SARS.18178.13 | 1.77 | 0.12 | 3.42 | 0.0018 |
| ARHGDIB.9846.32 | 1.73 | 0.15 | 3.30 | 0.0018 |
| LRP4.19558.10 | -1.63 | -3.05 | -0.22 | 0.0018 |
| NXF1.12453.161 | 1.70 | 0.16 | 3.23 | 0.0019 |
| CDC25B.12427.8 | 1.71 | 0.15 | 3.27 | 0.0019 |
| ZYX.13632.10 | 1.63 | 0.21 | 3.06 | 0.0020 |
| ENTHD2.7947.19 | 1.65 | 0.19 | 3.12 | 0.0020 |
| MRE11A.11319.106 | 1.66 | 0.18 | 3.14 | 0.0022 |
| PCMT1.18160.2 | 1.66 | 0.17 | 3.16 | 0.0024 |
| RDX.18373.13 | 1.68 | 0.15 | 3.20 | 0.0024 |
| PGD.4187.49 | 1.69 | 0.14 | 3.23 | 0.0024 |
| RIPK2.8993.151 | 1.66 | 0.17 | 3.14 | 0.0025 |
| CAPG.4968.50 | -1.86 | -3.69 | -0.03 | 0.0025 |
| MYOM3.13966.30 | -1.60 | -2.98 | -0.21 | 0.0025 |
| B3GNT8.9297.12 | -1.62 | -3.06 | -0.19 | 0.0026 |
| CNTNAP2.6965.19 | -1.62 | -3.05 | -0.19 | 0.0026 |
| EVL.11656.110 | 1.63 | 0.18 | 3.08 | 0.0027 |
| IL5RA.13686.2 | -1.63 | -3.08 | -0.18 | 0.0027 |
| SULF2.8305.18 | 1.87 | 0.03 | 3.71 | 0.0028 |
| LOXL3.15427.35 | 1.64 | 0.16 | 3.12 | 0.0028 |
| CDC42BPB.3629.60 | 1.65 | 0.16 | 3.15 | 0.0028 |
| NFASC.7179.69 | -1.61 | -3.03 | -0.19 | 0.0028 |
| MATN2.3325.2 | -1.65 | -3.15 | -0.15 | 0.0029 |
| TGFBR3.3009.3 | 1.58 | 0.21 | 2.96 | 0.0029 |
| TOM1L1.13652.2 | 1.60 | 0.18 | 3.03 | 0.0030 |
| SOCS3.11440.58 | 1.65 | 0.15 | 3.16 | 0.0031 |
| ALB.18380.78 | -1.62 | -3.07 | -0.17 | 0.0032 |
| INPP5E.11370.20 | 1.71 | 0.10 | 3.31 | 0.0032 |
| GAS6.15391.114 | -1.62 | -3.06 | -0.17 | 0.0032 |
| KIR2DS2.10428.1 | -1.61 | -3.05 | -0.17 | 0.0032 |
| CRH.5614.44 | 1.57 | 0.20 | 2.94 | 0.0033 |
| EGLN1.9901.28 | -1.61 | -3.06 | -0.17 | 0.0033 |
| CSNK1D.11289.31 | 1.67 | 0.12 | 3.21 | 0.0034 |
| NTRK2.4866.59 | -1.65 | -3.17 | -0.13 | 0.0036 |
| SERPINF2.3024.18 | 1.72 | 0.06 | 3.38 | 0.0037 |
| AZGP1.9312.8 | 1.53 | 0.22 | 2.84 | 0.0038 |
| CD58.10938.13 | -1.55 | -2.89 | -0.20 | 0.0038 |
| INHBA.INHBB.8467.9 | -1.63 | -3.12 | -0.14 | 0.0038 |
| SMAD1.9838.4 | 1.63 | 0.14 | 3.12 | 0.0038 |
| GP6.3194.36 | 1.64 | 0.12 | 3.17 | 0.0041 |
| TNFRSF1A.2654.19 | -1.69 | -3.31 | -0.07 | 0.0041 |
| AGR2.4959.2 | 1.70 | 0.06 | 3.34 | 0.0043 |
| HBZ.6919.3 | 1.64 | 0.11 | 3.16 | 0.0043 |
| TLR5.18935.14 | 1.58 | 0.16 | 3.01 | 0.0043 |
| OPCML.15622.13 | -1.62 | -3.10 | -0.13 | 0.0044 |
| HMGN1.9187.2 | 1.60 | 0.14 | 3.05 | 0.0044 |
| RBP5.19241.31 | -1.70 | -3.33 | -0.07 | 0.0044 |
| DNAJA2.11582.63 | 1.56 | 0.18 | 2.94 | 0.0045 |
| MREG.19145.4 | 1.63 | 0.12 | 3.14 | 0.0045 |
| METTL1.12514.16 | 1.61 | 0.13 | 3.10 | 0.0047 |
| SERPINE2.19154.41 | 1.67 | 0.08 | 3.26 | 0.0048 |
| ARRB1.12643.4 | 1.66 | 0.08 | 3.23 | 0.0049 |
| SNTB1.9078.207 | 1.58 | 0.13 | 3.03 | 0.0052 |
| CCL15.18289.16 | -1.60 | -3.07 | -0.12 | 0.0053 |
| PCDHAC1.7193.98 | 1.63 | 0.10 | 3.17 | 0.0053 |
| MAPKAPK2.3820.68 | 1.61 | 0.11 | 3.11 | 0.0054 |
| OOSP2.7106.37 | 1.60 | 0.11 | 3.10 | 0.0057 |
| UBE2D2.18842.24 | 1.69 | 0.03 | 3.34 | 0.0057 |
| RPS6KA1.12329.21 | 1.60 | 0.11 | 3.10 | 0.0058 |
| PCDHGA12.6938.21 | -1.62 | -3.16 | -0.09 | 0.0059 |
| CRIM1.15492.1 | -1.61 | -3.12 | -0.09 | 0.0061 |
| SERPINA10.13119.26 | 1.57 | 0.12 | 3.02 | 0.0062 |
| CRISP3.3187.52 | 1.61 | 0.09 | 3.12 | 0.0062 |
| F11.2190.55 | 1.54 | 0.15 | 2.92 | 0.0063 |
| OSBPL11.12878.60 | -1.60 | -3.10 | -0.10 | 0.0064 |
| LRP8.3323.37 | -1.57 | -3.03 | -0.12 | 0.0064 |
| AGO1.15312.14 | 1.57 | 0.11 | 3.04 | 0.0066 |
| DRG1.17849.6 | 1.55 | 0.13 | 2.98 | 0.0068 |
| RAB2B.19253.82 | 1.58 | 0.10 | 3.07 | 0.0068 |
| S100A4.14116.129 | 1.59 | 0.09 | 3.09 | 0.0068 |
| RCL.17785.11 | 1.57 | 0.11 | 3.02 | 0.0071 |
| CRHBP.6039.24 | 1.53 | 0.13 | 2.93 | 0.0072 |
| MOBKL1A.19177.7 | 1.67 | 0.02 | 3.31 | 0.0072 |
| TMPRSS11D.6547.83 | 1.64 | 0.04 | 3.24 | 0.0074 |
| DNASE1L2.6324.11 | -1.65 | -3.27 | -0.03 | 0.0074 |
| CHMP2A.17728.61 | 1.64 | 0.04 | 3.24 | 0.0074 |
| PKM2.4240.31 | 1.61 | 0.07 | 3.14 | 0.0075 |
| KRT18.5354.11 | 1.56 | 0.10 | 3.03 | 0.0079 |
| EFNA4.2614.28 | -1.62 | -3.21 | -0.04 | 0.0079 |
| TNFRSF17.2665.26 | -1.52 | -2.91 | -0.13 | 0.0079 |
| MFAP3L.8837.8 | 1.62 | 0.03 | 3.21 | 0.0084 |
| KLRB1.10809.14 | -1.56 | -3.00 | -0.11 | 0.0084 |
| CGREF1.6257.56 | 1.59 | 0.06 | 3.11 | 0.0086 |
| COL6A1.16828.8 | -1.54 | -2.97 | -0.10 | 0.0087 |
| DCUN1D1.17366.6 | 1.55 | 0.09 | 3.01 | 0.0087 |
| CEL.9796.4 | 1.58 | 0.07 | 3.09 | 0.0088 |
| FBLN5.15585.304 | -1.53 | -2.95 | -0.11 | 0.0089 |
| HSPA1A.14237.1 | 1.55 | 0.09 | 3.01 | 0.0090 |
| APOF.12370.30 | -1.56 | -3.04 | -0.08 | 0.0090 |
| PAK6.3827.22 | 1.43 | 0.18 | 2.68 | 0.0094 |
| PGM2.18330.7 | 1.58 | 0.05 | 3.12 | 0.0099 |
| LAMC2.9580.5 | -1.52 | -2.94 | -0.10 | 0.0099 |
| LOXL2.6504.65 | -1.52 | -2.95 | -0.10 | 0.0100 |
| PGAM2.15524.30 | 1.58 | 0.05 | 3.12 | 0.0100 |
| TREML1.11147.17 | 1.58 | 0.04 | 3.12 | 0.0101 |
| ANP32A.13073.14 | 1.72 | -0.08 | 3.52 | 0.0104 |
| COL25A1.7006.4 | 1.61 | 0.01 | 3.20 | 0.0104 |
| RNASE1.7211.2 | -1.63 | -3.26 | 0.00 | 0.0105 |
| PEBP1.4276.10 | 1.57 | 0.05 | 3.09 | 0.0108 |
| PPIA.3844.2 | 1.60 | 0.01 | 3.19 | 0.0110 |
| RET.3220.40 | 1.53 | 0.08 | 2.99 | 0.0110 |
| NCF1.17766.5 | 1.55 | 0.06 | 3.05 | 0.0111 |
| ASH1L.12622.96 | 1.50 | 0.10 | 2.90 | 0.0114 |
| PIK3CA.PIK3R1.3390.72 | 1.47 | 0.12 | 2.82 | 0.0114 |
| PNP.15435.4 | 1.61 | -0.01 | 3.24 | 0.0115 |
| COLEC11.4430.44 | -1.51 | -2.93 | -0.09 | 0.0116 |
| FAM3D.13102.1 | -1.56 | -3.09 | -0.04 | 0.0118 |
| TSTD1.19277.4 | 1.54 | 0.05 | 3.04 | 0.0119 |
| NCAM1.4498.62 | -1.53 | -3.01 | -0.06 | 0.0125 |
| CKAP2.5345.51 | 1.55 | 0.04 | 3.06 | 0.0125 |
| TGFBI.3283.21 | -1.52 | -2.97 | -0.06 | 0.0126 |
| SAR1A.17726.3 | 1.59 | 0.00 | 3.18 | 0.0127 |
| PPP1R1A.17706.4 | -1.56 | -3.09 | -0.03 | 0.0127 |
| SLC14A1.13430.50 | 1.52 | 0.07 | 2.96 | 0.0127 |
| REPIN1.13554.78 | 1.47 | 0.10 | 2.84 | 0.0131 |
| TNFAIP6.5036.50 | -1.49 | -2.89 | -0.09 | 0.0135 |
| COL3A1.18880.81 | -1.49 | -2.89 | -0.08 | 0.0135 |
| SLPI.4413.3 | -1.65 | -3.38 | 0.08 | 0.0137 |
| NODAL.15692.300 | -1.42 | -2.71 | -0.13 | 0.0143 |
| CTRC.5626.20 | 1.55 | 0.02 | 3.07 | 0.0143 |
| EPHA2.4834.61 | -1.59 | -3.19 | 0.02 | 0.0143 |
| DEFB1.6629.3 | 1.51 | 0.05 | 2.97 | 0.0147 |
| CNP.6609.22 | 1.50 | 0.06 | 2.95 | 0.0147 |
| TNFSF8.3421.54 | -1.53 | -3.03 | -0.03 | 0.0149 |
| NELL2.6022.57 | -1.50 | -2.94 | -0.06 | 0.0152 |
| NUDT16L1.12497.29 | 1.53 | 0.02 | 3.05 | 0.0155 |
| PARVA.13434.172 | -1.53 | -3.02 | -0.03 | 0.0155 |
| HDAC8.2859.69 | 1.50 | 0.05 | 2.95 | 0.0160 |
| ORC6L.12389.4 | 1.58 | -0.04 | 3.21 | 0.0161 |
| TREM2.16300.4 | -1.63 | -3.33 | 0.08 | 0.0161 |
| KLK14.15544.25 | -1.53 | -3.04 | -0.01 | 0.0164 |
| EWSR1.12988.49 | -1.60 | -3.25 | 0.05 | 0.0165 |
| IFNA10.14128.121 | -1.48 | -2.90 | -0.06 | 0.0165 |
| EHF.13387.55 | 1.62 | -0.08 | 3.33 | 0.0172 |
| SPON1.4297.62 | -1.55 | -3.12 | 0.01 | 0.0172 |
| BACH2.12756.3 | -1.43 | -2.75 | -0.10 | 0.0173 |
| APOC1.15364.101 | 1.48 | 0.05 | 2.91 | 0.0173 |
| MAPKAPK3.3822.54 | 1.49 | 0.05 | 2.93 | 0.0173 |
| COL15A1.8974.172 | -1.55 | -3.12 | 0.02 | 0.0179 |
| SIRT2.5030.52 | 1.64 | -0.10 | 3.38 | 0.0188 |
| RAC1.2870.29 | 1.55 | -0.03 | 3.13 | 0.0191 |
| PTPRJ.8250.2 | 1.53 | -0.02 | 3.08 | 0.0194 |
| PDE5A.5256.86 | 1.45 | 0.07 | 2.83 | 0.0194 |
| KCNMB3.8905.20 | 1.53 | -0.02 | 3.09 | 0.0194 |
| FSTL3.3438.10 | -1.52 | -3.04 | 0.00 | 0.0195 |
| CBLN1.9313.27 | -1.52 | -3.04 | 0.01 | 0.0195 |
| BRSK2.9790.28 | 1.49 | 0.02 | 2.96 | 0.0195 |
| EHD4.11421.10 | 1.50 | 0.01 | 3.00 | 0.0196 |
| MAPRE1.12469.19 | 1.56 | -0.04 | 3.16 | 0.0196 |
| EPHB3.9220.7 | -1.39 | -2.66 | -0.12 | 0.0197 |
| CST5.3803.10 | -1.58 | -3.22 | 0.07 | 0.0198 |
| ENAH.9757.29 | 1.36 | 0.13 | 2.59 | 0.0199 |
| RNASE3.15576.158 | -1.54 | -3.10 | 0.03 | 0.0200 |
| ANGPTL3.10391.1 | -1.51 | -3.03 | 0.01 | 0.0203 |
| MMP12.4496.60 | -1.58 | -3.24 | 0.07 | 0.0206 |
| TRIP10.17676.13 | 1.46 | 0.03 | 2.89 | 0.0212 |
| BMPR1B.10550.37 | -1.45 | -2.87 | -0.04 | 0.0216 |
| NTRK1.3477.63 | -1.48 | -2.94 | -0.01 | 0.0216 |
| GRN.4992.49 | -1.42 | -2.76 | -0.07 | 0.0217 |
| HSP90B1.6393.63 | 1.53 | -0.04 | 3.10 | 0.0218 |
| UBXN2B.18435.40 | 1.52 | -0.03 | 3.07 | 0.0221 |
| GP5.7185.29 | 1.51 | -0.03 | 3.05 | 0.0224 |
| FHIT.9826.135 | 1.52 | -0.03 | 3.06 | 0.0224 |
| DSTN.18883.4 | 1.50 | -0.01 | 3.01 | 0.0227 |
| ZNF334.12763.69 | 1.40 | 0.08 | 2.72 | 0.0229 |
| TKT.4306.4 | 1.52 | -0.04 | 3.08 | 0.0229 |
| FABP12.18888.37 | 1.55 | -0.07 | 3.16 | 0.0231 |
| LRRTM2.6904.14 | -1.49 | -2.99 | 0.01 | 0.0233 |
| SELP.4154.57 | 1.49 | -0.01 | 2.98 | 0.0233 |
| ACBD6.10075.75 | 1.49 | -0.02 | 3.01 | 0.0249 |
| IFNAR1.9183.7 | -1.43 | -2.82 | -0.04 | 0.0257 |
| BRICD5.6612.90 | 1.44 | 0.01 | 2.87 | 0.0269 |
| ANTXR1.10464.6 | -1.49 | -3.01 | 0.03 | 0.0270 |
| NDRG1.19215.7 | -1.37 | -2.67 | -0.08 | 0.0270 |
| AARS.12340.17 | 1.43 | 0.02 | 2.85 | 0.0270 |
| SRL.10940.25 | -1.51 | -3.07 | 0.06 | 0.0279 |
| KCNAB2.10015.119 | 1.48 | -0.04 | 3.01 | 0.0280 |
| CLIC4.15314.49 | 1.49 | -0.05 | 3.04 | 0.0292 |
| GABARAPL2.12494.99 | 1.48 | -0.04 | 2.99 | 0.0293 |
| TFF1.9185.15 | -1.47 | -2.96 | 0.03 | 0.0293 |
| AXIN2.8429.16 | -1.47 | -2.96 | 0.03 | 0.0293 |
| NNMT.19376.74 | -1.43 | -2.86 | -0.01 | 0.0294 |
| LINGO1.6620.82 | -1.48 | -3.01 | 0.04 | 0.0294 |
| YWHAQ.7625.27 | 1.50 | -0.07 | 3.07 | 0.0301 |
| APP.3171.57 | 1.49 | -0.06 | 3.03 | 0.0306 |
| C3.2683.1 | -1.20 | -2.21 | -0.19 | 0.0309 |
| VTA1.4209.60 | 1.45 | -0.03 | 2.94 | 0.0310 |
| DIMT1.12694.28 | 1.54 | -0.11 | 3.19 | 0.0314 |
| ITGA1.ITGB1.3503.4 | -1.40 | -2.79 | -0.02 | 0.0315 |
| BPNT1.17814.8 | 1.43 | -0.01 | 2.88 | 0.0318 |
| SEMA4F.9932.49 | 1.44 | -0.01 | 2.88 | 0.0318 |
| HTRA1.15594.47 | -1.38 | -2.73 | -0.04 | 0.0320 |
| BAMBI.8811.24 | -1.38 | -2.72 | -0.04 | 0.0324 |
| CAB39.17757.86 | 1.50 | -0.08 | 3.08 | 0.0331 |
| DPYSL3.12707.26 | 1.48 | -0.06 | 3.01 | 0.0331 |
| C9.3060.43 | -1.48 | -3.02 | 0.07 | 0.0337 |
| AP1G2.12714.38 | 1.44 | -0.03 | 2.91 | 0.0347 |
| GALE.11457.53 | 1.41 | -0.01 | 2.84 | 0.0350 |
| SMOC2.15635.4 | -1.46 | -2.99 | 0.06 | 0.0350 |
| LY75.16620.26 | -1.46 | -2.96 | 0.05 | 0.0352 |
| TMED10.6506.54 | -1.50 | -3.10 | 0.10 | 0.0353 |
| EPB41.4706.17 | 1.52 | -0.12 | 3.16 | 0.0362 |
| EBI3.10851.77 | -1.44 | -2.92 | 0.04 | 0.0363 |
| NAPA.4292.5 | 1.51 | -0.12 | 3.13 | 0.0367 |
| PGAM1.3896.5 | 1.40 | -0.01 | 2.82 | 0.0370 |
| PCTK1.16867.76 | 1.41 | -0.02 | 2.85 | 0.0380 |
| IL15RA.14054.17 | -1.46 | -3.00 | 0.08 | 0.0386 |
| NEK7.12703.6 | 1.44 | -0.05 | 2.93 | 0.0388 |
| CADM2.16907.3 | -1.48 | -3.06 | 0.10 | 0.0400 |
| ARL1.12392.30 | 1.45 | -0.08 | 2.98 | 0.0405 |
| GSTK1.13474.40 | 1.44 | -0.07 | 2.94 | 0.0419 |
| CD99L2.10539.30 | 1.42 | -0.04 | 2.88 | 0.0419 |
| TWF1.12871.10 | 1.35 | 0.03 | 2.66 | 0.0420 |
| NFU1.7770.25 | 1.43 | -0.06 | 2.92 | 0.0423 |
| MGA.11587.5 | 1.41 | -0.05 | 2.86 | 0.0450 |
| FABP1.11516.7 | -1.54 | -3.29 | 0.20 | 0.0451 |
| NPDC1.10424.31 | -1.46 | -3.03 | 0.11 | 0.0452 |
| MAN1B1.7071.23 | 1.38 | -0.02 | 2.79 | 0.0452 |
| PLAU.4158.54 | -1.25 | -2.39 | -0.10 | 0.0460 |
| MRRF.12355.223 | 1.48 | -0.14 | 3.10 | 0.0461 |
| ICAM2.5486.73 | 1.41 | -0.07 | 2.90 | 0.0475 |
| RALA.14332.3 | 1.44 | -0.09 | 2.96 | 0.0478 |
| GZMK.9545.156 | 1.36 | -0.01 | 2.73 | 0.0478 |

## Table 1.2 GLS

| **Biomarker** | **Estimate** | **CI - Lower** | **CI - Upper** | **P-value** |
| --- | --- | --- | --- | --- |
| LUM.13114.50 | -4.73 | -6.13 | -3.33 | 0.0000 |
| ROBO2.5116.62 | -4.48 | -5.77 | -3.19 | 0.0000 |
| NPPA.5443.62 | -4.32 | -5.62 | -3.02 | 0.0000 |
| LRRC32.7551.33 | -4.16 | -5.52 | -2.79 | 0.0000 |
| CHRDL1.3362.61 | -4.49 | -6.10 | -2.89 | 0.0000 |
| CELA1.6107.3 | -4.05 | -5.40 | -2.71 | 0.0000 |
| SCARF2.8956.96 | -4.30 | -5.82 | -2.79 | 0.0000 |
| PTK7.9525.1 | -3.80 | -5.08 | -2.53 | 0.0000 |
| LAMA2.LAMB1.LAMC1.18347.15 | -3.87 | -5.19 | -2.56 | 0.0000 |
| NCAM2.6507.16 | -4.01 | -5.44 | -2.57 | 0.0000 |
| ROBO1.5740.17 | -3.88 | -5.26 | -2.50 | 0.0000 |
| MMP2.4160.49 | -3.94 | -5.36 | -2.52 | 0.0000 |
| PLXNA1.9005.16 | -3.91 | -5.35 | -2.48 | 0.0000 |
| CDH7.7959.34 | 3.76 | 2.41 | 5.10 | 0.0000 |
| ANGPT2.2602.2 | -3.60 | -4.86 | -2.34 | 0.0000 |
| FCN3.5462.62 | 3.78 | 2.34 | 5.22 | 0.0000 |
| F7.3184.25 | 3.63 | 2.30 | 4.97 | 0.0000 |
| FCN2.13717.15 | 3.68 | 2.26 | 5.10 | 0.0000 |
| ENG.4908.6 | -3.55 | -4.88 | -2.23 | 0.0000 |
| HSPG2.15626.223 | -3.42 | -4.68 | -2.17 | 0.0000 |
| POSTN.3457.57 | -3.66 | -5.07 | -2.25 | 0.0000 |
| ITLN1.18830.1 | -3.61 | -5.03 | -2.20 | 0.0000 |
| LTBP4.13133.73 | -3.59 | -4.98 | -2.19 | 0.0000 |
| TIMP2.2278.61 | -3.57 | -4.97 | -2.17 | 0.0000 |
| BCAM.2816.50 | -3.68 | -5.14 | -2.22 | 0.0000 |
| OMD.5358.3 | -3.40 | -4.70 | -2.10 | 0.0000 |
| NRP2.15387.44 | -3.38 | -4.71 | -2.05 | 0.0000 |
| ENTPD6.8932.1 | 3.57 | 2.11 | 5.03 | 0.0000 |
| FIGF.13098.93 | -3.53 | -4.99 | -2.08 | 0.0000 |
| FGFR1.5532.53 | -3.41 | -4.79 | -2.04 | 0.0000 |
| NPTN.7194.36 | -3.41 | -4.78 | -2.03 | 0.0000 |
| ITIH5.8233.2 | -3.57 | -5.04 | -2.09 | 0.0000 |
| FADD.16593.3 | 3.42 | 2.03 | 4.82 | 0.0000 |
| NLGN1.15620.4 | -3.42 | -4.86 | -1.99 | 0.0000 |
| APCS.2474.54 | 3.33 | 1.97 | 4.68 | 0.0000 |
| TMEM132D.13416.8 | -3.25 | -4.55 | -1.95 | 0.0000 |
| GCK.12960.9 | 3.39 | 1.97 | 4.81 | 0.0000 |
| NOTUM.8252.2 | 3.15 | 1.89 | 4.41 | 0.0000 |
| GAS1.5463.22 | -3.47 | -5.01 | -1.93 | 0.0000 |
| RSPO4.8464.31 | -3.58 | -5.21 | -1.95 | 0.0000 |
| CD93.14136.234 | -3.35 | -4.80 | -1.91 | 0.0000 |
| FMOD.6367.66 | -3.28 | -4.66 | -1.90 | 0.0000 |
| IGF1R.4232.19 | -3.26 | -4.63 | -1.89 | 0.0000 |
| SCARA5.10419.1 | -3.19 | -4.55 | -1.84 | 0.0000 |
| BMPER.15368.3 | -3.23 | -4.63 | -1.83 | 0.0000 |
| PRKG1.13067.5 | 3.37 | 1.85 | 4.88 | 0.0000 |
| NAALADL1.17505.125 | 3.42 | 1.84 | 5.01 | 0.0000 |
| OMG.16908.5 | -3.09 | -4.42 | -1.77 | 0.0000 |
| MRC2.3041.55 | -3.23 | -4.68 | -1.78 | 0.0000 |
| KERA.10758.2 | -3.06 | -4.37 | -1.75 | 0.0000 |
| ADIPOQ.3554.24 | -3.03 | -4.33 | -1.73 | 0.0000 |
| AMH.4923.79 | 3.11 | 1.70 | 4.51 | 0.0000 |
| CSF1R.13682.47 | -3.06 | -4.44 | -1.69 | 0.0000 |
| ADAM23.7049.2 | -3.13 | -4.60 | -1.65 | 0.0000 |
| ESM1.3805.16 | -3.02 | -4.41 | -1.64 | 0.0000 |
| SLC35G2.13501.10 | 3.08 | 1.62 | 4.53 | 0.0000 |
| MTAP.9910.9 | 3.04 | 1.61 | 4.47 | 0.0000 |
| C1QBP.4967.1 | 3.26 | 1.67 | 4.85 | 0.0000 |
| COL28A1.10702.1 | -3.17 | -4.71 | -1.62 | 0.0000 |
| COLEC12.5457.5 | -2.95 | -4.31 | -1.58 | 0.0000 |
| KLK10.6227.1 | -3.02 | -4.45 | -1.59 | 0.0000 |
| NAT1.12632.14 | 3.08 | 1.59 | 4.56 | 0.0000 |
| GPC6.5350.14 | 2.97 | 1.60 | 4.34 | 0.0000 |
| SFTPD.19590.46 | -3.03 | -4.46 | -1.61 | 0.0000 |
| C11orf87.11116.16 | 2.93 | 1.56 | 4.29 | 0.0000 |
| NOTCH3.5108.72 | -2.99 | -4.42 | -1.55 | 0.0000 |
| VWC2.15308.108 | -3.12 | -4.68 | -1.55 | 0.0000 |
| NMB.9321.400 | -3.09 | -4.65 | -1.54 | 0.0000 |
| PSMA5.18925.24 | 2.83 | 1.53 | 4.14 | 0.0000 |
| F13B.5658.64 | 2.79 | 1.52 | 4.05 | 0.0000 |
| ENTPD5.4437.56 | 2.95 | 1.53 | 4.37 | 0.0000 |
| CASQ1.11263.57 | 3.01 | 1.52 | 4.49 | 0.0000 |
| TFPI.3336.50 | 2.96 | 1.48 | 4.44 | 0.0000 |
| STAR.10085.25 | 3.02 | 1.49 | 4.55 | 0.0000 |
| LRP10.16610.13 | -2.94 | -4.44 | -1.45 | 0.0000 |
| CHST15.4469.78 | -2.83 | -4.24 | -1.42 | 0.0000 |
| FLRT2.13122.19 | -2.94 | -4.46 | -1.42 | 0.0000 |
| ATP1B2.7218.87 | -2.87 | -4.32 | -1.42 | 0.0000 |
| SPOCK2.5491.12 | -2.72 | -4.03 | -1.41 | 0.0000 |
| SURF1.8009.121 | 2.78 | 1.40 | 4.16 | 0.0000 |
| TEX29.10557.6 | 2.80 | 1.40 | 4.19 | 0.0000 |
| ZHX3.10036.201 | 2.69 | 1.39 | 4.00 | 0.0000 |
| NPFF.5617.41 | 2.87 | 1.40 | 4.35 | 0.0000 |
| IGFBP7.3320.49 | -2.67 | -3.96 | -1.38 | 0.0000 |
| TMEM132B.8890.9 | -2.77 | -4.16 | -1.39 | 0.0000 |
| HSPA13.17515.6 | -2.73 | -4.08 | -1.38 | 0.0000 |
| HERC5.12934.1 | 2.93 | 1.40 | 4.45 | 0.0000 |
| SUMF1.6941.11 | 2.69 | 1.38 | 4.01 | 0.0000 |
| SVEP1.11109.56 | -2.56 | -3.76 | -1.37 | 0.0000 |
| ENPP2.16892.23 | -2.73 | -4.08 | -1.37 | 0.0000 |
| FGF9.19584.33 | -2.68 | -3.99 | -1.36 | 0.0000 |
| ALDH5A1.17792.158 | 2.82 | 1.37 | 4.27 | 0.0000 |
| CDH3.2643.57 | 2.67 | 1.36 | 3.99 | 0.0000 |
| CAMK1.3592.4 | 2.86 | 1.36 | 4.36 | 0.0000 |
| HNF4A.10041.3 | 2.70 | 1.35 | 4.06 | 0.0000 |
| BMP4.15667.39 | -2.72 | -4.09 | -1.35 | 0.0000 |
| NRCAM.5109.24 | -2.75 | -4.16 | -1.33 | 0.0000 |
| NTF3.4145.58 | -2.74 | -4.14 | -1.33 | 0.0000 |
| MRPL21.8942.2 | 2.81 | 1.33 | 4.30 | 0.0000 |
| FGF23.3807.1 | -2.57 | -3.80 | -1.34 | 0.0000 |
| CLSTN2.18882.7 | -2.60 | -3.88 | -1.32 | 0.0000 |
| MLF1.17820.170 | 2.89 | 1.31 | 4.48 | 0.0000 |
| BOC.4328.2 | -2.70 | -4.09 | -1.30 | 0.0000 |
| GREM2.5598.3 | -2.79 | -4.26 | -1.32 | 0.0000 |
| PLAUR.2652.15 | -2.77 | -4.24 | -1.29 | 0.0000 |
| RBBP9.10064.12 | 2.62 | 1.28 | 3.95 | 0.0000 |
| ITIH1.7955.195 | 2.66 | 1.28 | 4.03 | 0.0000 |
| ISLR2.13124.20 | -2.67 | -4.06 | -1.28 | 0.0000 |
| ARL11.12433.8 | 2.88 | 1.29 | 4.46 | 0.0000 |
| SERPINA11.9002.36 | -2.65 | -4.04 | -1.26 | 0.0000 |
| JAG1.5092.51 | -2.75 | -4.24 | -1.26 | 0.0000 |
| KDR.3651.50 | 2.77 | 1.25 | 4.29 | 0.0000 |
| CNTN4.3298.52 | -2.58 | -3.91 | -1.25 | 0.0000 |
| ATF6B.11387.3 | 2.61 | 1.25 | 3.98 | 0.0000 |
| NPPB.7655.11 | -3.68 | -5.43 | -1.93 | 0.0000 |
| RBP7.14208.3 | -2.73 | -4.19 | -1.26 | 0.0000 |
| EFEMP1.8480.29 | -2.84 | -4.43 | -1.24 | 0.0000 |
| SELM.15336.7 | -2.92 | -4.64 | -1.20 | 0.0000 |
| NAP1L2.13529.39 | 2.69 | 1.20 | 4.17 | 0.0000 |
| SEMA6B.5121.3 | -2.60 | -4.00 | -1.20 | 0.0000 |
| SPON2.8099.42 | -2.81 | -4.44 | -1.19 | 0.0000 |
| WIF1.16070.7 | -2.73 | -4.27 | -1.18 | 0.0000 |
| CPB2.3518.54 | 2.59 | 1.18 | 4.01 | 0.0000 |
| DKK3.3607.71 | -2.65 | -4.14 | -1.17 | 0.0000 |
| CLIC2.17837.5 | -2.74 | -4.26 | -1.22 | 0.0000 |
| SEMA6A.7945.10 | -2.49 | -3.80 | -1.17 | 0.0000 |
| RNF8.14663.44 | 2.80 | 1.18 | 4.42 | 0.0000 |
| NMES1.6406.3 | 2.75 | 1.15 | 4.34 | 0.0000 |
| NRP1.5542.22 | -2.56 | -3.96 | -1.16 | 0.0000 |
| SERPIND1.3316.58 | 2.70 | 1.14 | 4.25 | 0.0000 |
| PTPRU.8337.65 | -2.64 | -4.11 | -1.16 | 0.0000 |
| FCGR3B.3311.27 | -2.53 | -3.92 | -1.15 | 0.0000 |
| TAGLN.15640.54 | -2.87 | -4.62 | -1.12 | 0.0000 |
| SH2D3C.12704.26 | 2.66 | 1.13 | 4.20 | 0.0000 |
| LRRK2.10990.21 | 2.59 | 1.13 | 4.05 | 0.0000 |
| NR1H2.9016.12 | 2.76 | 1.11 | 4.41 | 0.0000 |
| GRB14.13628.58 | 2.77 | 1.12 | 4.42 | 0.0000 |
| COPS2.14029.42 | 2.64 | 1.11 | 4.16 | 0.0000 |
| EGFLAM.12338.27 | -2.56 | -3.99 | -1.12 | 0.0000 |
| ZP4.7766.25 | 2.87 | 1.13 | 4.62 | 0.0000 |
| FREM2.7246.4 | 2.57 | 1.11 | 4.03 | 0.0000 |
| DTX1.11430.49 | 2.50 | 1.12 | 3.88 | 0.0000 |
| RBFOX2.11462.8 | -2.50 | -3.88 | -1.11 | 0.0000 |
| PACAP.16322.10 | -2.53 | -3.95 | -1.11 | 0.0000 |
| S100A16.17836.17 | -2.55 | -4.00 | -1.10 | 0.0000 |
| MFAP4.5636.10 | -2.54 | -3.95 | -1.12 | 0.0000 |
| CETN2.13078.3 | 2.59 | 1.09 | 4.08 | 0.0000 |
| UBE2G2.9199.6 | 2.47 | 1.10 | 3.84 | 0.0000 |
| F9.4876.32 | 2.60 | 1.08 | 4.12 | 0.0000 |
| PLA2R1.10916.44 | -2.58 | -4.08 | -1.07 | 0.0000 |
| FJX1.7921.65 | -2.44 | -3.79 | -1.09 | 0.0000 |
| MINPP1.5586.66 | -2.48 | -3.89 | -1.08 | 0.0000 |
| CHL1.8958.51 | -2.46 | -3.86 | -1.06 | 0.0000 |
| ARFGAP1.11556.19 | 2.61 | 1.05 | 4.17 | 0.0000 |
| NEO1.8900.28 | -2.44 | -3.82 | -1.06 | 0.0000 |
| PIANP.9599.6 | -2.55 | -4.06 | -1.03 | 0.0000 |
| CPLX2.15321.8 | -2.55 | -4.07 | -1.04 | 0.0000 |
| FBLN1.6470.19 | -2.50 | -3.98 | -1.03 | 0.0000 |
| FUT10.7156.2 | -2.54 | -4.07 | -1.02 | 0.0000 |
| WFDC2.11388.75 | -2.61 | -4.21 | -1.00 | 0.0000 |
| KLB.19557.3 | 2.42 | 1.03 | 3.82 | 0.0000 |
| BCL2A1.3413.50 | 2.60 | 1.01 | 4.20 | 0.0000 |
| COX6C.8903.1 | 2.31 | 1.04 | 3.59 | 0.0000 |
| VAV3.9830.109 | 2.52 | 1.02 | 4.03 | 0.0000 |
| MSR1.15533.97 | -2.68 | -4.36 | -1.00 | 0.0000 |
| ROR2.7861.9 | -2.56 | -4.12 | -0.99 | 0.0000 |
| PAM.5620.13 | -2.41 | -3.81 | -1.02 | 0.0000 |
| CXADR.11204.80 | 2.47 | 1.00 | 3.94 | 0.0000 |
| NAB1.13933.276 | 2.49 | 0.99 | 3.99 | 0.0000 |
| ACVRL1.16318.12 | -2.58 | -4.20 | -0.97 | 0.0000 |
| PCSK9.5231.79 | 2.35 | 1.01 | 3.70 | 0.0000 |
| CRLF1.14747.9 | -2.42 | -3.86 | -0.99 | 0.0000 |
| KLKB1.4152.58 | 2.45 | 0.98 | 3.92 | 0.0000 |
| MGAT2.6909.40 | 2.43 | 0.98 | 3.87 | 0.0000 |
| SSR1.8106.15 | 2.47 | 0.97 | 3.97 | 0.0000 |
| GHR.2948.58 | 2.49 | 0.98 | 4.00 | 0.0000 |
| IL19.3035.80 | -2.34 | -3.69 | -0.99 | 0.0000 |
| TLR4.LY96.3647.49 | -2.46 | -3.95 | -0.97 | 0.0000 |
| TREM1.9266.1 | -2.55 | -4.15 | -0.94 | 0.0000 |
| TREML2.5736.1 | 2.41 | 0.97 | 3.86 | 0.0000 |
| UNC5B.15394.79 | -2.54 | -4.15 | -0.93 | 0.0000 |
| STC1.4930.21 | -2.36 | -3.77 | -0.96 | 0.0000 |
| COL6A2.16753.46 | -2.34 | -3.71 | -0.96 | 0.0000 |
| TRDMT1.12635.9 | 2.36 | 0.94 | 3.79 | 0.0000 |
| KREMEN1.17331.138 | -2.34 | -3.74 | -0.94 | 0.0000 |
| SORCS1.15636.49 | -2.34 | -3.74 | -0.93 | 0.0000 |
| TRABD.11262.39 | 2.56 | 0.89 | 4.23 | 0.0000 |
| SPARCL1.4467.49 | -2.30 | -3.68 | -0.92 | 0.0000 |
| PPP1R2.19152.4 | 2.40 | 0.90 | 3.91 | 0.0000 |
| PTK2B.8918.64 | 2.41 | 0.89 | 3.93 | 0.0000 |
| SRSF7.12987.12 | -2.36 | -3.82 | -0.90 | 0.0000 |
| SCGB2A1.5001.6 | 2.40 | 0.88 | 3.92 | 0.0000 |
| NTRK3.2658.27 | -2.33 | -3.77 | -0.90 | 0.0000 |
| ALCAM.5451.1 | -2.30 | -3.69 | -0.90 | 0.0000 |
| DIXDC1.13441.30 | 2.32 | 0.90 | 3.74 | 0.0000 |
| CCDC126.6388.21 | 2.27 | 0.90 | 3.63 | 0.0000 |
| CST3.2609.59 | -2.42 | -3.98 | -0.85 | 0.0000 |
| PLTP.15475.4 | -2.32 | -3.75 | -0.88 | 0.0000 |
| PXDN.13463.1 | -2.34 | -3.80 | -0.87 | 0.0000 |
| ASCC1.10647.18 | 2.46 | 0.85 | 4.06 | 0.0000 |
| RUFY1.11425.31 | 2.33 | 0.88 | 3.78 | 0.0000 |
| IBSP.3415.61 | -2.20 | -3.51 | -0.89 | 0.0000 |
| TCEAL8.19109.32 | 2.30 | 0.87 | 3.73 | 0.0000 |
| HFE2.3332.57 | 2.23 | 0.88 | 3.58 | 0.0000 |
| AGER.4125.52 | -2.32 | -3.77 | -0.88 | 0.0000 |
| GDF15.4374.45 | -2.34 | -3.84 | -0.85 | 0.0000 |
| JTB.9038.12 | -2.31 | -3.77 | -0.85 | 0.0000 |
| PLA2G12B.9380.2 | 2.24 | 0.86 | 3.62 | 0.0000 |
| RHOG.12540.25 | 2.34 | 0.84 | 3.85 | 0.0000 |
| PDYN.19638.9 | 2.28 | 0.85 | 3.71 | 0.0000 |
| NDC80.12730.3 | 2.38 | 0.83 | 3.93 | 0.0000 |
| DEAF1.6369.82 | 2.47 | 0.82 | 4.11 | 0.0000 |
| F10.3077.66 | 2.31 | 0.83 | 3.79 | 0.0000 |
| FABP2.15385.116 | -2.28 | -3.73 | -0.83 | 0.0000 |
| OLFML3.8660.5 | -2.29 | -3.75 | -0.82 | 0.0000 |
| ATP1B1.13392.13 | 2.18 | 0.84 | 3.51 | 0.0000 |
| EFNA5.2615.60 | -2.37 | -3.95 | -0.78 | 0.0000 |
| CXCL13.3487.32 | -2.11 | -3.38 | -0.84 | 0.0000 |
| KL.15384.15 | -2.25 | -3.71 | -0.80 | 0.0000 |
| MYL7.19296.51 | 2.30 | 0.78 | 3.82 | 0.0000 |
| APLN.6622.90 | 2.30 | 0.79 | 3.81 | 0.0000 |
| RNF122.11160.56 | 2.33 | 0.77 | 3.88 | 0.0000 |
| ITIH2.9326.33 | 2.25 | 0.78 | 3.72 | 0.0000 |
| CDH5.2819.23 | -2.21 | -3.64 | -0.78 | 0.0000 |
| PKN1.12562.1 | 2.25 | 0.77 | 3.74 | 0.0000 |
| THBS3.8982.65 | -2.24 | -3.72 | -0.77 | 0.0000 |
| GDF2.4880.21 | -2.20 | -3.61 | -0.78 | 0.0000 |
| FGB.18890.227 | 2.29 | 0.74 | 3.84 | 0.0000 |
| RNASE6.5646.20 | -2.21 | -3.67 | -0.76 | 0.0000 |
| ADAMTS4.2809.25 | 2.35 | 0.73 | 3.96 | 0.0000 |
| SH3BP2.7769.29 | 2.17 | 0.77 | 3.57 | 0.0000 |
| CCDC80.3234.23 | -2.36 | -4.01 | -0.71 | 0.0000 |
| SLITRK5.4568.17 | -2.23 | -3.73 | -0.74 | 0.0000 |
| PRSS22.4534.10 | -2.15 | -3.53 | -0.77 | 0.0000 |
| GPNMB.8289.8 | -2.19 | -3.62 | -0.75 | 0.0000 |
| CYR61.6264.9 | -2.18 | -3.62 | -0.74 | 0.0000 |
| TPST2.8024.64 | 2.23 | 0.72 | 3.74 | 0.0000 |
| HS3ST5.10731.10 | 2.28 | 0.74 | 3.83 | 0.0000 |
| UMOD.9451.20 | -2.14 | -3.55 | -0.74 | 0.0000 |
| CHFR.11320.29 | 2.23 | 0.71 | 3.76 | 0.0000 |
| PENK.9076.25 | -2.24 | -3.77 | -0.70 | 0.0000 |
| ASB9.19601.15 | -2.38 | -4.13 | -0.63 | 0.0000 |
| WARS.9870.17 | -2.01 | -3.28 | -0.75 | 0.0000 |
| C1QTNF5.7810.20 | -2.09 | -3.46 | -0.73 | 0.0000 |
| CA9.3798.71 | -2.15 | -3.60 | -0.71 | 0.0000 |
| KIF3A.13961.18 | 2.21 | 0.69 | 3.73 | 0.0000 |
| PCDHGA10.6321.65 | -2.11 | -3.49 | -0.73 | 0.0000 |
| COL1A1.11140.56 | -2.15 | -3.60 | -0.69 | 0.0000 |
| GALNT16.8923.94 | -2.18 | -3.69 | -0.68 | 0.0000 |
| FGA.FGB.FGG.2796.62 | -2.16 | -3.64 | -0.68 | 0.0000 |
| SPARC.3043.49 | 2.24 | 0.65 | 3.83 | 0.0000 |
| CTSS.3181.50 | -2.23 | -3.82 | -0.64 | 0.0000 |
| TRA2B.12373.73 | -2.20 | -3.75 | -0.66 | 0.0000 |
| EFNA2.14124.6 | -2.23 | -3.83 | -0.64 | 0.0000 |
| IGFBP1.2771.35 | -2.10 | -3.51 | -0.68 | 0.0000 |
| WNT3A.13236.25 | 2.30 | 0.68 | 3.93 | 0.0000 |
| CHST11.7779.86 | -2.13 | -3.59 | -0.67 | 0.0000 |
| CES1.15487.164 | 2.17 | 0.66 | 3.68 | 0.0000 |
| CCL5.5480.49 | 2.22 | 0.63 | 3.80 | 0.0000 |
| STOML1.17344.23 | 2.09 | 0.68 | 3.51 | 0.0000 |
| IGSF3.9715.15 | -2.08 | -3.49 | -0.68 | 0.0000 |
| NPTXR.15511.37 | -2.10 | -3.54 | -0.66 | 0.0000 |
| HLA.DRB3.6962.5 | 2.14 | 0.65 | 3.63 | 0.0000 |
| IL26.16760.2 | 2.05 | 0.68 | 3.41 | 0.0000 |
| HSPA1B.18901.26 | -2.15 | -3.65 | -0.65 | 0.0000 |
| CTRB2.5648.28 | 2.08 | 0.66 | 3.50 | 0.0000 |
| EFS.12572.236 | 2.15 | 0.64 | 3.66 | 0.0000 |
| TPMT.11218.84 | 2.15 | 0.64 | 3.66 | 0.0000 |
| RGMA.5483.1 | 2.09 | 0.65 | 3.53 | 0.0000 |
| ARHGAP36.6289.78 | 2.07 | 0.66 | 3.48 | 0.0000 |
| MMP7.2789.26 | -2.17 | -3.72 | -0.62 | 0.0000 |
| CLCA2.8950.4 | 2.19 | 0.61 | 3.78 | 0.0000 |
| PRKCQ.3817.18 | 2.07 | 0.65 | 3.49 | 0.0000 |
| CLEC2D.7054.87 | 2.19 | 0.60 | 3.79 | 0.0000 |
| PPIB.4718.5 | -2.00 | -3.32 | -0.67 | 0.0000 |
| PLXND1.19561.216 | -2.18 | -3.75 | -0.60 | 0.0000 |
| FAM3B.9177.6 | -2.18 | -3.77 | -0.60 | 0.0000 |
| CD248.15491.20 | -2.16 | -3.72 | -0.61 | 0.0000 |
| OPTC.15430.165 | 2.14 | 0.61 | 3.66 | 0.0000 |
| PCSK2.6117.4 | -2.06 | -3.47 | -0.65 | 0.0000 |
| IL6ST.2620.4 | -2.08 | -3.54 | -0.63 | 0.0000 |
| TPPP2.12800.5 | -2.18 | -3.78 | -0.59 | 0.0000 |
| ECM1.3366.51 | -2.10 | -3.59 | -0.62 | 0.0000 |
| NBL1.2944.66 | -2.23 | -3.88 | -0.57 | 0.0000 |
| PCDHGC3.7859.21 | 2.15 | 0.60 | 3.70 | 0.0000 |
| CD72.7009.8 | -2.06 | -3.49 | -0.63 | 0.0000 |
| IL11RA.18216.22 | -2.15 | -3.71 | -0.60 | 0.0000 |
| ADAMTSL2.6379.62 | -1.93 | -3.19 | -0.67 | 0.0000 |
| PROS1.2700.56 | 2.10 | 0.61 | 3.60 | 0.0000 |
| PLEK.7875.86 | 2.09 | 0.61 | 3.56 | 0.0000 |
| PRCP.5722.78 | 2.00 | 0.64 | 3.36 | 0.0000 |
| FSTL1.13112.179 | -2.05 | -3.48 | -0.62 | 0.0000 |
| SERPINF1.9211.19 | 2.04 | 0.62 | 3.45 | 0.0000 |
| CREBBP.13614.6 | 2.07 | 0.61 | 3.54 | 0.0000 |
| TDO2.9880.33 | 2.07 | 0.61 | 3.53 | 0.0000 |
| IL1RL1.4234.8 | -1.95 | -3.25 | -0.65 | 0.0000 |
| HSPA8.5903.91 | -1.91 | -3.18 | -0.64 | 0.0000 |
| RTN4R.5105.2 | 2.02 | 0.60 | 3.44 | 0.0000 |
| MAP3K3.12990.39 | -2.04 | -3.49 | -0.60 | 0.0000 |
| COL11A2.11278.4 | -1.99 | -3.38 | -0.60 | 0.0000 |
| REG4.11102.22 | -2.23 | -3.96 | -0.50 | 0.0000 |
| C8G.14708.59 | 1.98 | 0.60 | 3.37 | 0.0000 |
| CD59.11514.196 | -2.10 | -3.67 | -0.53 | 0.0000 |
| TRIL.6527.1 | -1.99 | -3.40 | -0.57 | 0.0000 |
| FLRT3.13123.3 | -2.04 | -3.55 | -0.54 | 0.0000 |
| FURIN.6276.16 | -1.97 | -3.38 | -0.57 | 0.0000 |
| IL1B.3037.62 | -1.94 | -3.31 | -0.58 | 0.0000 |
| IMPDH2.5250.53 | 2.05 | 0.52 | 3.58 | 0.0000 |
| NPTX1.9256.78 | -1.97 | -3.39 | -0.55 | 0.0000 |
| CXCL10.4141.79 | -2.01 | -3.48 | -0.54 | 0.0000 |
| OSCAR.7116.31 | 2.08 | 0.49 | 3.68 | 0.0000 |
| HERC4.7860.9 | 2.05 | 0.50 | 3.60 | 0.0000 |
| TNFSF15.2968.61 | -2.11 | -3.74 | -0.47 | 0.0000 |
| PANK3.12658.72 | 2.16 | 0.46 | 3.85 | 0.0000 |
| TNFRSF11B.8304.50 | -2.09 | -3.70 | -0.48 | 0.0000 |
| CCL14.2900.53 | -1.94 | -3.35 | -0.54 | 0.0000 |
| SPIN1.18210.12 | -1.92 | -3.29 | -0.54 | 0.0000 |
| AIMP1.2714.78 | 1.89 | 0.55 | 3.23 | 0.0000 |
| IGFLR1.7244.16 | -2.09 | -3.72 | -0.46 | 0.0000 |
| DOK1.16831.7 | 1.91 | 0.54 | 3.28 | 0.0000 |
| PCDH9.10558.26 | -1.98 | -3.46 | -0.50 | 0.0000 |
| PSAP.6207.10 | -1.93 | -3.34 | -0.52 | 0.0000 |
| ARL6.18415.16 | 2.06 | 0.48 | 3.64 | 0.0000 |
| PRDX6.5018.68 | 2.05 | 0.46 | 3.64 | 0.0000 |
| APOA1BP.16621.77 | 2.00 | 0.48 | 3.52 | 0.0000 |
| ARL15.18411.83 | 1.88 | 0.53 | 3.23 | 0.0000 |
| SCGB1D2.6508.68 | 2.09 | 0.45 | 3.73 | 0.0000 |
| SRC.15433.4 | 2.03 | 0.46 | 3.61 | 0.0000 |
| DCTPP1.4314.12 | -1.99 | -3.50 | -0.48 | 0.0000 |
| PSME2.17694.32 | 1.95 | 0.49 | 3.41 | 0.0000 |
| CBR1.12381.26 | -1.99 | -3.52 | -0.47 | 0.0000 |
| CPZ.6493.9 | -1.87 | -3.22 | -0.52 | 0.0000 |
| KAAG1.19492.5 | 1.87 | 0.51 | 3.23 | 0.0000 |
| DNAJB12.8006.12 | -2.04 | -3.66 | -0.42 | 0.0000 |
| KRT5.11177.16 | 1.82 | 0.53 | 3.10 | 0.0000 |
| QPCTL.8866.53 | -1.94 | -3.43 | -0.46 | 0.0000 |
| EMC4.13516.46 | 1.93 | 0.47 | 3.40 | 0.0000 |
| GLTPD2.7948.129 | 1.92 | 0.47 | 3.37 | 0.0000 |
| SEPSECS.17357.33 | 1.87 | 0.50 | 3.25 | 0.0000 |
| NAPG.17773.26 | 1.96 | 0.45 | 3.47 | 0.0000 |
| HSP90AB1.5467.15 | 1.98 | 0.43 | 3.54 | 0.0000 |
| DCLK1.17156.72 | -1.93 | -3.41 | -0.45 | 0.0000 |
| CXCL6.3495.15 | 1.86 | 0.49 | 3.24 | 0.0000 |
| HRAS.18900.37 | 1.96 | 0.43 | 3.50 | 0.0000 |
| F13A1.F13B.16927.9 | 1.81 | 0.50 | 3.12 | 0.0000 |
| SULT1B1.12671.35 | 1.89 | 0.47 | 3.31 | 0.0000 |
| CHST9.11646.4 | -1.83 | -3.17 | -0.49 | 0.0000 |
| BGN.3284.75 | -1.95 | -3.48 | -0.43 | 0.0000 |
| SUMF2.6069.71 | -1.91 | -3.38 | -0.45 | 0.0000 |
| INHBB.13676.46 | -1.88 | -3.30 | -0.46 | 0.0000 |
| LY86.3623.84 | 2.08 | 0.37 | 3.78 | 0.0000 |
| C14orf93.6439.59 | 2.01 | 0.40 | 3.62 | 0.0000 |
| SPSB1.13942.140 | 1.95 | 0.41 | 3.49 | 0.0000 |
| BCHE.15514.26 | 1.85 | 0.46 | 3.23 | 0.0000 |
| RAD1.12670.15 | 1.87 | 0.45 | 3.28 | 0.0000 |
| TNNT2.5315.22 | -1.82 | -3.16 | -0.48 | 0.0000 |
| CD33.3166.92 | -1.91 | -3.41 | -0.41 | 0.0000 |
| KLK13.11152.46 | -1.91 | -3.40 | -0.42 | 0.0000 |
| ELF5.13457.33 | 2.02 | 0.35 | 3.68 | 0.0000 |
| IL3RA.13744.37 | -1.94 | -3.48 | -0.40 | 0.0000 |
| PPA2.18307.71 | 1.93 | 0.40 | 3.46 | 0.0000 |
| SKP1.3902.21 | 2.02 | 0.34 | 3.69 | 0.0000 |
| WISP2.6392.7 | -2.13 | -4.00 | -0.26 | 0.0000 |
| PTPN4.14254.27 | 1.87 | 0.42 | 3.32 | 0.0000 |
| BMP6.8459.10 | -1.81 | -3.18 | -0.45 | 0.0000 |
| TNXB.5698.60 | -1.88 | -3.37 | -0.39 | 0.0000 |
| FLT4.16035.8 | -1.84 | -3.28 | -0.41 | 0.0000 |
| IGFBP5.19581.15 | -1.78 | -3.12 | -0.44 | 0.0000 |
| BDNF.14047.78 | 1.88 | 0.38 | 3.39 | 0.0000 |
| NUDT9.9482.110 | 1.91 | 0.36 | 3.45 | 0.0000 |
| HAAO.5861.78 | 1.87 | 0.38 | 3.37 | 0.0000 |
| SNTA1.19274.80 | 1.87 | 0.38 | 3.36 | 0.0000 |
| CDCP1.16818.200 | -1.96 | -3.59 | -0.32 | 0.0001 |
| RGS19.12713.365 | 1.75 | 0.43 | 3.07 | 0.0001 |
| FAM171A2.13479.8 | 1.85 | 0.38 | 3.32 | 0.0001 |
| IMPDH1.5229.90 | 1.86 | 0.37 | 3.36 | 0.0001 |
| TEK.3773.15 | -1.89 | -3.42 | -0.35 | 0.0001 |
| PRDM4.12779.30 | 1.88 | 0.36 | 3.40 | 0.0001 |
| ADH6.18206.18 | 1.90 | 0.34 | 3.47 | 0.0001 |
| CXCL12.3516.60 | -1.85 | -3.33 | -0.37 | 0.0001 |
| PSME1.5918.5 | 1.87 | 0.36 | 3.38 | 0.0001 |
| LRRC15.6557.50 | -1.79 | -3.18 | -0.40 | 0.0001 |
| LILRA5.7787.25 | 1.85 | 0.37 | 3.33 | 0.0001 |
| CLIC5.12475.48 | -1.92 | -3.51 | -0.32 | 0.0001 |
| SYT4.17355.56 | 1.87 | 0.35 | 3.39 | 0.0001 |
| SELL.4831.4 | -1.83 | -3.28 | -0.37 | 0.0001 |
| CRIP2.9053.16 | -1.88 | -3.41 | -0.34 | 0.0001 |
| IL1R1.2991.9 | -1.81 | -3.24 | -0.38 | 0.0001 |
| ARPP19.4963.19 | 1.85 | 0.34 | 3.36 | 0.0001 |
| ADAMTSL1.16890.37 | -2.00 | -3.77 | -0.23 | 0.0001 |
| MLL2.13623.4 | 1.86 | 0.33 | 3.40 | 0.0001 |
| MOBKL3.19332.1 | 1.90 | 0.31 | 3.50 | 0.0001 |
| CAND1.13937.75 | 1.88 | 0.32 | 3.43 | 0.0001 |
| SPP1.13113.7 | -1.73 | -3.07 | -0.40 | 0.0001 |
| TNFAIP8.12563.2 | 1.83 | 0.34 | 3.33 | 0.0001 |
| PAPSS1.14007.22 | 1.81 | 0.35 | 3.27 | 0.0001 |
| TMEM132A.7871.16 | -1.75 | -3.11 | -0.38 | 0.0001 |
| SPOCK1.5490.53 | 1.86 | 0.31 | 3.40 | 0.0001 |
| F8.13499.30 | -1.87 | -3.43 | -0.30 | 0.0001 |
| EDAR.2977.7 | 1.77 | 0.37 | 3.17 | 0.0001 |
| AGT.3484.60 | 1.72 | 0.39 | 3.05 | 0.0001 |
| SLC6A14.13053.6 | 1.79 | 0.34 | 3.25 | 0.0001 |
| DSC2.13126.52 | -1.79 | -3.24 | -0.34 | 0.0001 |
| ACTN1.9843.5 | 1.90 | 0.28 | 3.51 | 0.0001 |
| TPRKB.12417.46 | 1.74 | 0.37 | 3.10 | 0.0001 |
| APOL1.9506.10 | 1.73 | 0.38 | 3.08 | 0.0002 |
| KIR3DL1.18907.97 | 1.84 | 0.31 | 3.38 | 0.0002 |
| ADGRF1.11243.90 | 1.84 | 0.30 | 3.38 | 0.0002 |
| NDUFB11.7747.47 | 1.79 | 0.34 | 3.24 | 0.0002 |
| MATN3.19361.78 | -1.75 | -3.15 | -0.36 | 0.0002 |
| SIGLEC8.7864.3 | 1.72 | 0.37 | 3.06 | 0.0002 |
| YWHAB.14156.33 | 1.84 | 0.30 | 3.37 | 0.0002 |
| AFM.4763.31 | 1.76 | 0.35 | 3.17 | 0.0002 |
| TIMP1.2211.9 | -1.83 | -3.36 | -0.30 | 0.0002 |
| PDE4A.18918.86 | 1.73 | 0.36 | 3.10 | 0.0002 |
| AKR1A1.4192.10 | 1.84 | 0.29 | 3.38 | 0.0002 |
| IGHG4.13231.90 | 1.69 | 0.38 | 2.99 | 0.0002 |
| GEM.12817.1 | 1.78 | 0.32 | 3.23 | 0.0002 |
| NAMPT.5011.11 | -1.54 | -2.65 | -0.44 | 0.0002 |
| EGFR.2677.1 | 1.82 | 0.29 | 3.35 | 0.0002 |
| SPINK7.10974.20 | -1.82 | -3.36 | -0.28 | 0.0002 |
| NRXN1.8971.9 | 1.72 | 0.34 | 3.11 | 0.0002 |
| ACP5.3232.28 | 1.72 | 0.34 | 3.09 | 0.0002 |
| ASPN.6451.64 | -1.78 | -3.26 | -0.30 | 0.0003 |
| ZNF329.12803.9 | 1.81 | 0.27 | 3.34 | 0.0003 |
| S100A6.13090.17 | 1.88 | 0.22 | 3.54 | 0.0003 |
| IGLON5.6478.2 | -1.72 | -3.13 | -0.32 | 0.0003 |
| NQO1.9837.60 | -1.81 | -3.34 | -0.27 | 0.0003 |
| CENPW.8864.59 | 1.69 | 0.34 | 3.05 | 0.0003 |
| CHAD.13460.4 | -1.69 | -3.05 | -0.33 | 0.0003 |
| INA.11436.6 | -1.79 | -3.31 | -0.27 | 0.0003 |
| DLK1.6373.54 | 1.77 | 0.28 | 3.26 | 0.0003 |
| RPS6KB1.15608.5 | 1.78 | 0.27 | 3.29 | 0.0003 |
| PRSS35.9983.97 | 1.85 | 0.22 | 3.48 | 0.0004 |
| LEG1.7154.92 | 1.78 | 0.27 | 3.30 | 0.0004 |
| NTN4.3327.27 | -1.67 | -3.00 | -0.34 | 0.0004 |
| PPA1.5021.13 | 1.77 | 0.27 | 3.27 | 0.0004 |
| MENT.5744.12 | 1.76 | 0.28 | 3.23 | 0.0004 |
| APOA5.15363.32 | 1.72 | 0.30 | 3.14 | 0.0004 |
| RNGTT.12847.27 | 1.82 | 0.24 | 3.39 | 0.0004 |
| COL18A1.2201.17 | -1.79 | -3.32 | -0.26 | 0.0004 |
| ERP29.13728.19 | 1.88 | 0.21 | 3.54 | 0.0004 |
| NAGK.3894.15 | 1.80 | 0.25 | 3.34 | 0.0004 |
| COLGALT1.5638.23 | 1.80 | 0.25 | 3.35 | 0.0004 |
| HSP90AA1.2625.53 | 1.79 | 0.25 | 3.33 | 0.0004 |
| CALCOCO2.12534.10 | -1.72 | -3.15 | -0.30 | 0.0004 |
| CHMP2B.17350.13 | 1.70 | 0.30 | 3.10 | 0.0004 |
| FKBP6.12529.32 | 1.78 | 0.24 | 3.32 | 0.0005 |
| VIT.6234.74 | -1.84 | -3.48 | -0.20 | 0.0005 |
| SULT1A3.13944.3 | 1.75 | 0.26 | 3.24 | 0.0005 |
| NPTX2.6521.35 | 1.77 | 0.24 | 3.29 | 0.0006 |
| CNPY4.15465.79 | 1.76 | 0.24 | 3.28 | 0.0006 |
| QDPR.11257.1 | 1.79 | 0.23 | 3.34 | 0.0006 |
| C4BPA.9449.150 | 1.71 | 0.27 | 3.14 | 0.0006 |
| ANXA6.5335.73 | 1.73 | 0.25 | 3.20 | 0.0007 |
| MAPK14.5007.1 | 1.78 | 0.22 | 3.33 | 0.0007 |
| PLXDC2.11342.59 | 1.77 | 0.22 | 3.32 | 0.0007 |
| PLXNC1.4564.2 | -1.73 | -3.23 | -0.23 | 0.0008 |
| CRKL.9877.28 | 1.87 | 0.15 | 3.58 | 0.0008 |
| ST6GALNAC6.7228.2 | -1.76 | -3.31 | -0.21 | 0.0008 |
| AMY1A.7918.114 | -1.68 | -3.08 | -0.27 | 0.0008 |
| LRRC4C.9369.174 | -1.74 | -3.25 | -0.23 | 0.0008 |
| PDCD6IP.18174.79 | 1.83 | 0.17 | 3.49 | 0.0008 |
| CASP10.5340.24 | 1.75 | 0.22 | 3.28 | 0.0008 |
| IL17RA.2992.59 | -1.67 | -3.08 | -0.27 | 0.0008 |
| TFRC.6895.1 | -1.66 | -3.03 | -0.28 | 0.0009 |
| STIP1.5489.18 | 1.73 | 0.23 | 3.23 | 0.0009 |
| COTL1.4905.63 | 1.80 | 0.17 | 3.42 | 0.0009 |
| C1QTNF1.6304.8 | -1.64 | -3.01 | -0.28 | 0.0009 |
| CEACAM7.7184.13 | 1.68 | 0.25 | 3.11 | 0.0009 |
| ADAMTS3.8845.2 | 1.70 | 0.23 | 3.18 | 0.0010 |
| TXNDC5.11212.7 | 1.72 | 0.23 | 3.21 | 0.0010 |
| ASAH1.5748.20 | 1.71 | 0.22 | 3.20 | 0.0010 |
| WFDC1.9316.67 | -1.79 | -3.40 | -0.17 | 0.0010 |
| RAB6B.14271.23 | 1.72 | 0.22 | 3.22 | 0.0010 |
| APOA2.7127.3 | -1.68 | -3.11 | -0.24 | 0.0011 |
| KIAA0040.14603.51 | 1.68 | 0.24 | 3.11 | 0.0011 |
| KLK11.2831.29 | -1.74 | -3.29 | -0.20 | 0.0011 |
| ANGPTL4.3796.79 | -1.70 | -3.19 | -0.22 | 0.0011 |
| DHRS9.17467.1 | 1.71 | 0.21 | 3.20 | 0.0012 |
| F2.5316.54 | 1.69 | 0.21 | 3.17 | 0.0013 |
| GAA.9385.4 | -1.64 | -3.04 | -0.25 | 0.0013 |
| WFDC3.6384.19 | 1.72 | 0.19 | 3.26 | 0.0013 |
| CADM1.3326.58 | -1.71 | -3.24 | -0.19 | 0.0014 |
| FBXL5.12846.3 | 1.73 | 0.17 | 3.29 | 0.0014 |
| CHCHD10.11270.17 | -1.64 | -3.05 | -0.24 | 0.0014 |
| G6PD.19297.4 | 1.70 | 0.20 | 3.20 | 0.0014 |
| CFD.2946.52 | -1.75 | -3.34 | -0.16 | 0.0014 |
| GDF7.16756.30 | 1.62 | 0.25 | 2.99 | 0.0014 |
| HSPB6.19127.1 | -1.78 | -3.42 | -0.13 | 0.0014 |
| YWHAZ.5858.6 | 1.75 | 0.15 | 3.35 | 0.0015 |
| NSF.13992.12 | 1.77 | 0.14 | 3.40 | 0.0015 |
| BCL2L2.13097.11 | 1.70 | 0.19 | 3.21 | 0.0015 |
| CPXM1.6255.74 | 1.67 | 0.21 | 3.12 | 0.0015 |
| RSPO1.16614.27 | -1.80 | -3.48 | -0.12 | 0.0015 |
| PTGDS.10514.5 | -1.76 | -3.39 | -0.13 | 0.0016 |
| ALDH1A3.9835.16 | 1.68 | 0.20 | 3.16 | 0.0016 |
| CSH1.CSH2.13103.125 | 1.75 | 0.15 | 3.34 | 0.0017 |
| SEMA3E.5363.51 | -1.66 | -3.11 | -0.21 | 0.0017 |
| MAPK10.15418.25 | 1.73 | 0.15 | 3.31 | 0.0018 |
| SARS.18178.13 | 1.77 | 0.12 | 3.42 | 0.0018 |
| ARHGDIB.9846.32 | 1.73 | 0.15 | 3.30 | 0.0018 |
| LRP4.19558.10 | -1.63 | -3.05 | -0.22 | 0.0018 |
| NXF1.12453.161 | 1.70 | 0.16 | 3.23 | 0.0019 |
| CDC25B.12427.8 | 1.71 | 0.15 | 3.27 | 0.0019 |
| ZYX.13632.10 | 1.63 | 0.21 | 3.06 | 0.0020 |
| ENTHD2.7947.19 | 1.65 | 0.19 | 3.12 | 0.0020 |
| MRE11A.11319.106 | 1.66 | 0.18 | 3.14 | 0.0022 |
| PCMT1.18160.2 | 1.66 | 0.17 | 3.16 | 0.0024 |
| RDX.18373.13 | 1.68 | 0.15 | 3.20 | 0.0024 |
| PGD.4187.49 | 1.69 | 0.14 | 3.23 | 0.0024 |
| RIPK2.8993.151 | 1.66 | 0.17 | 3.14 | 0.0025 |
| CAPG.4968.50 | -1.86 | -3.69 | -0.03 | 0.0025 |
| MYOM3.13966.30 | -1.60 | -2.98 | -0.21 | 0.0025 |
| B3GNT8.9297.12 | -1.62 | -3.06 | -0.19 | 0.0026 |
| CNTNAP2.6965.19 | -1.62 | -3.05 | -0.19 | 0.0026 |
| EVL.11656.110 | 1.63 | 0.18 | 3.08 | 0.0027 |
| IL5RA.13686.2 | -1.63 | -3.08 | -0.18 | 0.0027 |
| SULF2.8305.18 | 1.87 | 0.03 | 3.71 | 0.0028 |
| LOXL3.15427.35 | 1.64 | 0.16 | 3.12 | 0.0028 |
| CDC42BPB.3629.60 | 1.65 | 0.16 | 3.15 | 0.0028 |
| NFASC.7179.69 | -1.61 | -3.03 | -0.19 | 0.0028 |
| MATN2.3325.2 | -1.65 | -3.15 | -0.15 | 0.0029 |
| TGFBR3.3009.3 | 1.58 | 0.21 | 2.96 | 0.0029 |
| TOM1L1.13652.2 | 1.60 | 0.18 | 3.03 | 0.0030 |
| SOCS3.11440.58 | 1.65 | 0.15 | 3.16 | 0.0031 |
| ALB.18380.78 | -1.62 | -3.07 | -0.17 | 0.0032 |
| INPP5E.11370.20 | 1.71 | 0.10 | 3.31 | 0.0032 |
| GAS6.15391.114 | -1.62 | -3.06 | -0.17 | 0.0032 |
| KIR2DS2.10428.1 | -1.61 | -3.05 | -0.17 | 0.0032 |
| CRH.5614.44 | 1.57 | 0.20 | 2.94 | 0.0033 |
| EGLN1.9901.28 | -1.61 | -3.06 | -0.17 | 0.0033 |
| CSNK1D.11289.31 | 1.67 | 0.12 | 3.21 | 0.0034 |
| NTRK2.4866.59 | -1.65 | -3.17 | -0.13 | 0.0036 |
| SERPINF2.3024.18 | 1.72 | 0.06 | 3.38 | 0.0037 |
| AZGP1.9312.8 | 1.53 | 0.22 | 2.84 | 0.0038 |
| CD58.10938.13 | -1.55 | -2.89 | -0.20 | 0.0038 |
| INHBA.INHBB.8467.9 | -1.63 | -3.12 | -0.14 | 0.0038 |
| SMAD1.9838.4 | 1.63 | 0.14 | 3.12 | 0.0038 |
| GP6.3194.36 | 1.64 | 0.12 | 3.17 | 0.0041 |
| TNFRSF1A.2654.19 | -1.69 | -3.31 | -0.07 | 0.0041 |
| AGR2.4959.2 | 1.70 | 0.06 | 3.34 | 0.0043 |
| HBZ.6919.3 | 1.64 | 0.11 | 3.16 | 0.0043 |
| TLR5.18935.14 | 1.58 | 0.16 | 3.01 | 0.0043 |
| OPCML.15622.13 | -1.62 | -3.10 | -0.13 | 0.0044 |
| HMGN1.9187.2 | 1.60 | 0.14 | 3.05 | 0.0044 |
| RBP5.19241.31 | -1.70 | -3.33 | -0.07 | 0.0044 |
| DNAJA2.11582.63 | 1.56 | 0.18 | 2.94 | 0.0045 |
| MREG.19145.4 | 1.63 | 0.12 | 3.14 | 0.0045 |
| METTL1.12514.16 | 1.61 | 0.13 | 3.10 | 0.0047 |
| SERPINE2.19154.41 | 1.67 | 0.08 | 3.26 | 0.0048 |
| ARRB1.12643.4 | 1.66 | 0.08 | 3.23 | 0.0049 |
| SNTB1.9078.207 | 1.58 | 0.13 | 3.03 | 0.0052 |
| CCL15.18289.16 | -1.60 | -3.07 | -0.12 | 0.0053 |
| PCDHAC1.7193.98 | 1.63 | 0.10 | 3.17 | 0.0053 |
| MAPKAPK2.3820.68 | 1.61 | 0.11 | 3.11 | 0.0054 |
| OOSP2.7106.37 | 1.60 | 0.11 | 3.10 | 0.0057 |
| UBE2D2.18842.24 | 1.69 | 0.03 | 3.34 | 0.0057 |
| RPS6KA1.12329.21 | 1.60 | 0.11 | 3.10 | 0.0058 |
| PCDHGA12.6938.21 | -1.62 | -3.16 | -0.09 | 0.0059 |
| CRIM1.15492.1 | -1.61 | -3.12 | -0.09 | 0.0061 |
| SERPINA10.13119.26 | 1.57 | 0.12 | 3.02 | 0.0062 |
| CRISP3.3187.52 | 1.61 | 0.09 | 3.12 | 0.0062 |
| F11.2190.55 | 1.54 | 0.15 | 2.92 | 0.0063 |
| OSBPL11.12878.60 | -1.60 | -3.10 | -0.10 | 0.0064 |
| LRP8.3323.37 | -1.57 | -3.03 | -0.12 | 0.0064 |
| AGO1.15312.14 | 1.57 | 0.11 | 3.04 | 0.0066 |
| DRG1.17849.6 | 1.55 | 0.13 | 2.98 | 0.0068 |
| RAB2B.19253.82 | 1.58 | 0.10 | 3.07 | 0.0068 |
| S100A4.14116.129 | 1.59 | 0.09 | 3.09 | 0.0068 |
| RCL.17785.11 | 1.57 | 0.11 | 3.02 | 0.0071 |
| CRHBP.6039.24 | 1.53 | 0.13 | 2.93 | 0.0072 |
| MOBKL1A.19177.7 | 1.67 | 0.02 | 3.31 | 0.0072 |
| TMPRSS11D.6547.83 | 1.64 | 0.04 | 3.24 | 0.0074 |
| DNASE1L2.6324.11 | -1.65 | -3.27 | -0.03 | 0.0074 |
| CHMP2A.17728.61 | 1.64 | 0.04 | 3.24 | 0.0074 |
| PKM2.4240.31 | 1.61 | 0.07 | 3.14 | 0.0075 |
| KRT18.5354.11 | 1.56 | 0.10 | 3.03 | 0.0079 |
| EFNA4.2614.28 | -1.62 | -3.21 | -0.04 | 0.0079 |
| TNFRSF17.2665.26 | -1.52 | -2.91 | -0.13 | 0.0079 |
| MFAP3L.8837.8 | 1.62 | 0.03 | 3.21 | 0.0084 |
| KLRB1.10809.14 | -1.56 | -3.00 | -0.11 | 0.0084 |
| CGREF1.6257.56 | 1.59 | 0.06 | 3.11 | 0.0086 |
| COL6A1.16828.8 | -1.54 | -2.97 | -0.10 | 0.0087 |
| DCUN1D1.17366.6 | 1.55 | 0.09 | 3.01 | 0.0087 |
| CEL.9796.4 | 1.58 | 0.07 | 3.09 | 0.0088 |
| FBLN5.15585.304 | -1.53 | -2.95 | -0.11 | 0.0089 |
| HSPA1A.14237.1 | 1.55 | 0.09 | 3.01 | 0.0090 |
| APOF.12370.30 | -1.56 | -3.04 | -0.08 | 0.0090 |
| PAK6.3827.22 | 1.43 | 0.18 | 2.68 | 0.0094 |
| PGM2.18330.7 | 1.58 | 0.05 | 3.12 | 0.0099 |
| LAMC2.9580.5 | -1.52 | -2.94 | -0.10 | 0.0099 |
| LOXL2.6504.65 | -1.52 | -2.95 | -0.10 | 0.0100 |
| PGAM2.15524.30 | 1.58 | 0.05 | 3.12 | 0.0100 |
| TREML1.11147.17 | 1.58 | 0.04 | 3.12 | 0.0101 |
| ANP32A.13073.14 | 1.72 | -0.08 | 3.52 | 0.0104 |
| COL25A1.7006.4 | 1.61 | 0.01 | 3.20 | 0.0104 |
| RNASE1.7211.2 | -1.63 | -3.26 | 0.00 | 0.0105 |
| PEBP1.4276.10 | 1.57 | 0.05 | 3.09 | 0.0108 |
| PPIA.3844.2 | 1.60 | 0.01 | 3.19 | 0.0110 |
| RET.3220.40 | 1.53 | 0.08 | 2.99 | 0.0110 |
| NCF1.17766.5 | 1.55 | 0.06 | 3.05 | 0.0111 |
| ASH1L.12622.96 | 1.50 | 0.10 | 2.90 | 0.0114 |
| PIK3CA.PIK3R1.3390.72 | 1.47 | 0.12 | 2.82 | 0.0114 |
| PNP.15435.4 | 1.61 | -0.01 | 3.24 | 0.0115 |
| COLEC11.4430.44 | -1.51 | -2.93 | -0.09 | 0.0116 |
| FAM3D.13102.1 | -1.56 | -3.09 | -0.04 | 0.0118 |
| TSTD1.19277.4 | 1.54 | 0.05 | 3.04 | 0.0119 |
| NCAM1.4498.62 | -1.53 | -3.01 | -0.06 | 0.0125 |
| CKAP2.5345.51 | 1.55 | 0.04 | 3.06 | 0.0125 |
| TGFBI.3283.21 | -1.52 | -2.97 | -0.06 | 0.0126 |
| SAR1A.17726.3 | 1.59 | 0.00 | 3.18 | 0.0127 |
| PPP1R1A.17706.4 | -1.56 | -3.09 | -0.03 | 0.0127 |
| SLC14A1.13430.50 | 1.52 | 0.07 | 2.96 | 0.0127 |
| REPIN1.13554.78 | 1.47 | 0.10 | 2.84 | 0.0131 |
| TNFAIP6.5036.50 | -1.49 | -2.89 | -0.09 | 0.0135 |
| COL3A1.18880.81 | -1.49 | -2.89 | -0.08 | 0.0135 |
| SLPI.4413.3 | -1.65 | -3.38 | 0.08 | 0.0137 |
| NODAL.15692.300 | -1.42 | -2.71 | -0.13 | 0.0143 |
| CTRC.5626.20 | 1.55 | 0.02 | 3.07 | 0.0143 |
| EPHA2.4834.61 | -1.59 | -3.19 | 0.02 | 0.0143 |
| DEFB1.6629.3 | 1.51 | 0.05 | 2.97 | 0.0147 |
| CNP.6609.22 | 1.50 | 0.06 | 2.95 | 0.0147 |
| TNFSF8.3421.54 | -1.53 | -3.03 | -0.03 | 0.0149 |
| NELL2.6022.57 | -1.50 | -2.94 | -0.06 | 0.0152 |
| NUDT16L1.12497.29 | 1.53 | 0.02 | 3.05 | 0.0155 |
| PARVA.13434.172 | -1.53 | -3.02 | -0.03 | 0.0155 |
| HDAC8.2859.69 | 1.50 | 0.05 | 2.95 | 0.0160 |
| ORC6L.12389.4 | 1.58 | -0.04 | 3.21 | 0.0161 |
| TREM2.16300.4 | -1.63 | -3.33 | 0.08 | 0.0161 |
| KLK14.15544.25 | -1.53 | -3.04 | -0.01 | 0.0164 |
| EWSR1.12988.49 | -1.60 | -3.25 | 0.05 | 0.0165 |
| IFNA10.14128.121 | -1.48 | -2.90 | -0.06 | 0.0165 |
| EHF.13387.55 | 1.62 | -0.08 | 3.33 | 0.0172 |
| SPON1.4297.62 | -1.55 | -3.12 | 0.01 | 0.0172 |
| BACH2.12756.3 | -1.43 | -2.75 | -0.10 | 0.0173 |
| APOC1.15364.101 | 1.48 | 0.05 | 2.91 | 0.0173 |
| MAPKAPK3.3822.54 | 1.49 | 0.05 | 2.93 | 0.0173 |
| COL15A1.8974.172 | -1.55 | -3.12 | 0.02 | 0.0179 |
| SIRT2.5030.52 | 1.64 | -0.10 | 3.38 | 0.0188 |
| RAC1.2870.29 | 1.55 | -0.03 | 3.13 | 0.0191 |
| PTPRJ.8250.2 | 1.53 | -0.02 | 3.08 | 0.0194 |
| PDE5A.5256.86 | 1.45 | 0.07 | 2.83 | 0.0194 |
| KCNMB3.8905.20 | 1.53 | -0.02 | 3.09 | 0.0194 |
| FSTL3.3438.10 | -1.52 | -3.04 | 0.00 | 0.0195 |
| CBLN1.9313.27 | -1.52 | -3.04 | 0.01 | 0.0195 |
| BRSK2.9790.28 | 1.49 | 0.02 | 2.96 | 0.0195 |
| EHD4.11421.10 | 1.50 | 0.01 | 3.00 | 0.0196 |
| MAPRE1.12469.19 | 1.56 | -0.04 | 3.16 | 0.0196 |
| EPHB3.9220.7 | -1.39 | -2.66 | -0.12 | 0.0197 |
| CST5.3803.10 | -1.58 | -3.22 | 0.07 | 0.0198 |
| ENAH.9757.29 | 1.36 | 0.13 | 2.59 | 0.0199 |
| RNASE3.15576.158 | -1.54 | -3.10 | 0.03 | 0.0200 |
| ANGPTL3.10391.1 | -1.51 | -3.03 | 0.01 | 0.0203 |
| MMP12.4496.60 | -1.58 | -3.24 | 0.07 | 0.0206 |
| TRIP10.17676.13 | 1.46 | 0.03 | 2.89 | 0.0212 |
| BMPR1B.10550.37 | -1.45 | -2.87 | -0.04 | 0.0216 |
| NTRK1.3477.63 | -1.48 | -2.94 | -0.01 | 0.0216 |
| GRN.4992.49 | -1.42 | -2.76 | -0.07 | 0.0217 |
| HSP90B1.6393.63 | 1.53 | -0.04 | 3.10 | 0.0218 |
| UBXN2B.18435.40 | 1.52 | -0.03 | 3.07 | 0.0221 |
| GP5.7185.29 | 1.51 | -0.03 | 3.05 | 0.0224 |
| FHIT.9826.135 | 1.52 | -0.03 | 3.06 | 0.0224 |
| DSTN.18883.4 | 1.50 | -0.01 | 3.01 | 0.0227 |
| ZNF334.12763.69 | 1.40 | 0.08 | 2.72 | 0.0229 |
| TKT.4306.4 | 1.52 | -0.04 | 3.08 | 0.0229 |
| FABP12.18888.37 | 1.55 | -0.07 | 3.16 | 0.0231 |
| LRRTM2.6904.14 | -1.49 | -2.99 | 0.01 | 0.0233 |
| SELP.4154.57 | 1.49 | -0.01 | 2.98 | 0.0233 |
| ACBD6.10075.75 | 1.49 | -0.02 | 3.01 | 0.0249 |
| IFNAR1.9183.7 | -1.43 | -2.82 | -0.04 | 0.0257 |
| BRICD5.6612.90 | 1.44 | 0.01 | 2.87 | 0.0269 |
| ANTXR1.10464.6 | -1.49 | -3.01 | 0.03 | 0.0270 |
| NDRG1.19215.7 | -1.37 | -2.67 | -0.08 | 0.0270 |
| AARS.12340.17 | 1.43 | 0.02 | 2.85 | 0.0270 |
| SRL.10940.25 | -1.51 | -3.07 | 0.06 | 0.0279 |
| KCNAB2.10015.119 | 1.48 | -0.04 | 3.01 | 0.0280 |
| CLIC4.15314.49 | 1.49 | -0.05 | 3.04 | 0.0292 |
| GABARAPL2.12494.99 | 1.48 | -0.04 | 2.99 | 0.0293 |
| TFF1.9185.15 | -1.47 | -2.96 | 0.03 | 0.0293 |
| AXIN2.8429.16 | -1.47 | -2.96 | 0.03 | 0.0293 |
| NNMT.19376.74 | -1.43 | -2.86 | -0.01 | 0.0294 |
| LINGO1.6620.82 | -1.48 | -3.01 | 0.04 | 0.0294 |
| YWHAQ.7625.27 | 1.50 | -0.07 | 3.07 | 0.0301 |
| APP.3171.57 | 1.49 | -0.06 | 3.03 | 0.0306 |
| C3.2683.1 | -1.20 | -2.21 | -0.19 | 0.0309 |
| VTA1.4209.60 | 1.45 | -0.03 | 2.94 | 0.0310 |
| DIMT1.12694.28 | 1.54 | -0.11 | 3.19 | 0.0314 |
| ITGA1.ITGB1.3503.4 | -1.40 | -2.79 | -0.02 | 0.0315 |
| BPNT1.17814.8 | 1.43 | -0.01 | 2.88 | 0.0318 |
| SEMA4F.9932.49 | 1.44 | -0.01 | 2.88 | 0.0318 |
| HTRA1.15594.47 | -1.38 | -2.73 | -0.04 | 0.0320 |
| BAMBI.8811.24 | -1.38 | -2.72 | -0.04 | 0.0324 |
| CAB39.17757.86 | 1.50 | -0.08 | 3.08 | 0.0331 |
| DPYSL3.12707.26 | 1.48 | -0.06 | 3.01 | 0.0331 |
| C9.3060.43 | -1.48 | -3.02 | 0.07 | 0.0337 |
| AP1G2.12714.38 | 1.44 | -0.03 | 2.91 | 0.0347 |
| GALE.11457.53 | 1.41 | -0.01 | 2.84 | 0.0350 |
| SMOC2.15635.4 | -1.46 | -2.99 | 0.06 | 0.0350 |
| LY75.16620.26 | -1.46 | -2.96 | 0.05 | 0.0352 |
| TMED10.6506.54 | -1.50 | -3.10 | 0.10 | 0.0353 |
| EPB41.4706.17 | 1.52 | -0.12 | 3.16 | 0.0362 |
| EBI3.10851.77 | -1.44 | -2.92 | 0.04 | 0.0363 |
| NAPA.4292.5 | 1.51 | -0.12 | 3.13 | 0.0367 |
| PGAM1.3896.5 | 1.40 | -0.01 | 2.82 | 0.0370 |
| PCTK1.16867.76 | 1.41 | -0.02 | 2.85 | 0.0380 |
| IL15RA.14054.17 | -1.46 | -3.00 | 0.08 | 0.0386 |
| NEK7.12703.6 | 1.44 | -0.05 | 2.93 | 0.0388 |
| CADM2.16907.3 | -1.48 | -3.06 | 0.10 | 0.0400 |
| ARL1.12392.30 | 1.45 | -0.08 | 2.98 | 0.0405 |
| GSTK1.13474.40 | 1.44 | -0.07 | 2.94 | 0.0419 |
| CD99L2.10539.30 | 1.42 | -0.04 | 2.88 | 0.0419 |
| TWF1.12871.10 | 1.35 | 0.03 | 2.66 | 0.0420 |
| NFU1.7770.25 | 1.43 | -0.06 | 2.92 | 0.0423 |
| MGA.11587.5 | 1.41 | -0.05 | 2.86 | 0.0450 |
| FABP1.11516.7 | -1.54 | -3.29 | 0.20 | 0.0451 |
| NPDC1.10424.31 | -1.46 | -3.03 | 0.11 | 0.0452 |
| MAN1B1.7071.23 | 1.38 | -0.02 | 2.79 | 0.0452 |
| PLAU.4158.54 | -1.25 | -2.39 | -0.10 | 0.0460 |
| MRRF.12355.223 | 1.48 | -0.14 | 3.10 | 0.0461 |
| ICAM2.5486.73 | 1.41 | -0.07 | 2.90 | 0.0475 |
| RALA.14332.3 | 1.44 | -0.09 | 2.96 | 0.0478 |
| GZMK.9545.156 | 1.36 | -0.01 | 2.73 | 0.0478 |

## Table 1.3 LASr

| **Biomarker** | **Estimate** | **CI - Lower** | **CI - Upper** | **P-value** |
| --- | --- | --- | --- | --- |
| NPPA.5443.62 | 1.84 | 1.44 | 2.24 | 0.0000 |
| CHRDL1.3362.61 | 2.01 | 1.47 | 2.54 | 0.0000 |
| NOTUM.8252.2 | -1.67 | -2.07 | -1.27 | 0.0000 |
| ANGPT2.2602.2 | 1.60 | 1.19 | 2.02 | 0.0000 |
| SCARF2.8956.96 | 1.70 | 1.21 | 2.18 | 0.0000 |
| FIGF.13098.93 | 1.57 | 1.10 | 2.04 | 0.0000 |
| RSPO4.8464.31 | 1.66 | 1.13 | 2.19 | 0.0000 |
| NPPB.7655.11 | 1.65 | 1.11 | 2.18 | 0.0000 |
| KERA.10758.2 | 1.46 | 1.04 | 1.88 | 0.0000 |
| PLA2G12B.9380.2 | -1.46 | -1.89 | -1.02 | 0.0000 |
| ENTPD5.4437.56 | -1.46 | -1.90 | -1.02 | 0.0000 |
| MMP2.4160.49 | 1.47 | 1.01 | 1.92 | 0.0000 |
| EFEMP1.8480.29 | 1.50 | 1.01 | 1.99 | 0.0000 |
| TIMP2.2278.61 | 1.45 | 0.99 | 1.91 | 0.0000 |
| KLKB1.4152.58 | -1.45 | -1.93 | -0.98 | 0.0000 |
| NRP1.5542.22 | 1.42 | 0.97 | 1.88 | 0.0000 |
| ITLN1.18830.1 | 1.47 | 0.98 | 1.97 | 0.0000 |
| PLAUR.2652.15 | 1.47 | 0.97 | 1.98 | 0.0000 |
| LUM.13114.50 | 1.43 | 0.95 | 1.90 | 0.0000 |
| IGFBP7.3320.49 | 1.36 | 0.93 | 1.79 | 0.0000 |
| HFE2.3332.57 | -1.38 | -1.82 | -0.93 | 0.0000 |
| TMEM132D.13416.8 | 1.36 | 0.92 | 1.80 | 0.0000 |
| MFAP4.5636.10 | 1.38 | 0.92 | 1.85 | 0.0000 |
| SURF1.8009.121 | -1.35 | -1.80 | -0.91 | 0.0000 |
| TAGLN.15640.54 | 1.53 | 0.95 | 2.11 | 0.0000 |
| LTBP4.13133.73 | 1.39 | 0.91 | 1.87 | 0.0000 |
| CELA1.6107.3 | 1.36 | 0.90 | 1.83 | 0.0000 |
| CDH7.7959.34 | -1.33 | -1.78 | -0.88 | 0.0000 |
| WFDC2.11388.75 | 1.42 | 0.91 | 1.94 | 0.0000 |
| FCN2.13717.15 | -1.37 | -1.85 | -0.89 | 0.0000 |
| LAMA2.LAMB1.LAMC1.18347.15 | 1.31 | 0.87 | 1.74 | 0.0000 |
| TREM1.9266.1 | 1.42 | 0.90 | 1.95 | 0.0000 |
| F7.3184.25 | -1.31 | -1.77 | -0.86 | 0.0000 |
| EGFR.2677.1 | -1.40 | -1.91 | -0.89 | 0.0000 |
| GAS1.5463.22 | 1.38 | 0.87 | 1.89 | 0.0000 |
| SFTPD.19590.46 | 1.30 | 0.85 | 1.75 | 0.0000 |
| BMPER.15368.3 | 1.32 | 0.85 | 1.80 | 0.0000 |
| PTK7.9525.1 | 1.29 | 0.84 | 1.74 | 0.0000 |
| MYL7.19296.51 | -1.34 | -1.83 | -0.85 | 0.0000 |
| F13B.5658.64 | -1.24 | -1.66 | -0.81 | 0.0000 |
| CST3.2609.59 | 1.35 | 0.84 | 1.87 | 0.0000 |
| ESM1.3805.16 | 1.31 | 0.82 | 1.79 | 0.0000 |
| CLSTN2.18882.7 | 1.21 | 0.80 | 1.63 | 0.0000 |
| DTX1.11430.49 | -1.24 | -1.67 | -0.80 | 0.0000 |
| MSR1.15533.97 | 1.36 | 0.83 | 1.89 | 0.0000 |
| NRP2.15387.44 | 1.24 | 0.80 | 1.68 | 0.0000 |
| SLC35G2.13501.10 | -1.26 | -1.73 | -0.79 | 0.0000 |
| CHST15.4469.78 | 1.21 | 0.78 | 1.65 | 0.0000 |
| SVEP1.11109.56 | 1.21 | 0.77 | 1.65 | 0.0000 |
| ROBO2.5116.62 | 1.21 | 0.76 | 1.65 | 0.0000 |
| RBP7.14208.3 | 1.21 | 0.76 | 1.67 | 0.0000 |
| LRP10.16610.13 | 1.26 | 0.77 | 1.76 | 0.0000 |
| FAP.5029.3 | -1.23 | -1.71 | -0.76 | 0.0000 |
| DNAJB12.8006.12 | 1.30 | 0.78 | 1.83 | 0.0000 |
| AGER.4125.52 | 1.21 | 0.75 | 1.67 | 0.0000 |
| MINPP1.5586.66 | 1.23 | 0.76 | 1.71 | 0.0000 |
| CDH3.2643.57 | -1.16 | -1.59 | -0.73 | 0.0000 |
| GDF15.4374.45 | 1.26 | 0.75 | 1.76 | 0.0000 |
| FCN3.5462.62 | -1.25 | -1.74 | -0.75 | 0.0000 |
| ACP5.3232.28 | -1.20 | -1.66 | -0.74 | 0.0000 |
| CASQ1.11263.57 | -1.27 | -1.78 | -0.75 | 0.0000 |
| NAT1.12632.14 | -1.27 | -1.79 | -0.75 | 0.0000 |
| PXDN.13463.1 | 1.24 | 0.74 | 1.74 | 0.0000 |
| LRRC32.7551.33 | 1.24 | 0.74 | 1.74 | 0.0000 |
| FCGR3B.3311.27 | 1.19 | 0.73 | 1.66 | 0.0000 |
| ADAMTSL2.6379.62 | 1.13 | 0.71 | 1.56 | 0.0000 |
| FBLN5.15585.304 | 1.18 | 0.72 | 1.64 | 0.0000 |
| IGF1R.4232.19 | 1.19 | 0.72 | 1.65 | 0.0000 |
| NMB.9321.400 | 1.31 | 0.74 | 1.87 | 0.0000 |
| CCL14.2900.53 | 1.18 | 0.72 | 1.65 | 0.0000 |
| STOML1.17344.23 | -1.18 | -1.65 | -0.72 | 0.0000 |
| WNT3A.13236.25 | -1.23 | -1.73 | -0.72 | 0.0000 |
| BMP6.8459.10 | 1.16 | 0.70 | 1.61 | 0.0000 |
| EGLN1.9901.28 | 1.12 | 0.69 | 1.56 | 0.0000 |
| COL28A1.10702.1 | 1.22 | 0.70 | 1.73 | 0.0000 |
| BMP1.3348.49 | -1.13 | -1.58 | -0.68 | 0.0000 |
| CD27.5412.53 | -1.20 | -1.70 | -0.69 | 0.0000 |
| COL6A2.16753.46 | 1.19 | 0.69 | 1.69 | 0.0000 |
| FGFR1.5532.53 | 1.16 | 0.69 | 1.63 | 0.0000 |
| HSPG2.15626.223 | 1.10 | 0.67 | 1.53 | 0.0000 |
| WARS.9870.17 | 1.10 | 0.67 | 1.52 | 0.0000 |
| HERC5.12934.1 | -1.19 | -1.69 | -0.68 | 0.0000 |
| RBBP9.10064.12 | -1.12 | -1.58 | -0.67 | 0.0000 |
| RNASE1.7211.2 | 1.17 | 0.67 | 1.67 | 0.0000 |
| GHR.2948.58 | -1.15 | -1.64 | -0.66 | 0.0000 |
| ADIPOQ.3554.24 | 1.09 | 0.65 | 1.53 | 0.0000 |
| CD93.14136.234 | 1.11 | 0.65 | 1.58 | 0.0000 |
| MTAP.9910.9 | -1.14 | -1.63 | -0.65 | 0.0000 |
| KDR.3651.50 | -1.16 | -1.68 | -0.65 | 0.0000 |
| FSTL3.3438.10 | 1.17 | 0.65 | 1.70 | 0.0000 |
| TNNT2.5315.22 | 1.09 | 0.64 | 1.55 | 0.0000 |
| PLTP.15475.4 | 1.12 | 0.64 | 1.60 | 0.0000 |
| BMP4.15667.39 | 1.09 | 0.63 | 1.55 | 0.0000 |
| BCHE.15514.26 | -1.07 | -1.52 | -0.62 | 0.0000 |
| JAG1.5092.51 | 1.14 | 0.63 | 1.64 | 0.0000 |
| RBFOX2.11462.8 | 1.12 | 0.63 | 1.61 | 0.0000 |
| IBSP.3415.61 | 1.07 | 0.62 | 1.52 | 0.0000 |
| AMH.4923.79 | -1.08 | -1.55 | -0.61 | 0.0000 |
| FADD.16593.3 | -1.09 | -1.57 | -0.61 | 0.0000 |
| TPMT.11218.84 | -1.13 | -1.64 | -0.61 | 0.0000 |
| LRRK2.10990.21 | -1.08 | -1.56 | -0.60 | 0.0001 |
| EPO.5813.58 | 1.00 | 0.59 | 1.41 | 0.0001 |
| INHBB.13676.46 | 1.08 | 0.60 | 1.55 | 0.0001 |
| TFRC.6895.1 | 1.04 | 0.59 | 1.48 | 0.0001 |
| IGFBP2.8469.41 | 1.05 | 0.59 | 1.50 | 0.0001 |
| ENG.4908.6 | 1.05 | 0.59 | 1.50 | 0.0001 |
| PRCP.5722.78 | -1.06 | -1.53 | -0.59 | 0.0001 |
| C9.3060.43 | 1.09 | 0.59 | 1.60 | 0.0001 |
| DKK3.3607.71 | 1.08 | 0.58 | 1.59 | 0.0002 |
| ROR2.7861.9 | 1.14 | 0.59 | 1.69 | 0.0002 |
| OLFML3.8660.5 | 1.05 | 0.58 | 1.52 | 0.0002 |
| TRA2B.12373.73 | 1.07 | 0.58 | 1.56 | 0.0002 |
| CXCL10.4141.79 | 1.06 | 0.58 | 1.54 | 0.0002 |
| WFDC1.9316.67 | 1.11 | 0.58 | 1.63 | 0.0002 |
| PSMA5.18925.24 | -1.00 | -1.44 | -0.57 | 0.0002 |
| NPDC1.10424.31 | 1.10 | 0.58 | 1.62 | 0.0002 |
| ITIH5.8233.2 | 1.07 | 0.57 | 1.56 | 0.0002 |
| EWSR1.12988.49 | 1.09 | 0.57 | 1.61 | 0.0002 |
| CAMK1.3592.4 | -1.06 | -1.55 | -0.57 | 0.0002 |
| SERPINA3.2879.9 | 0.95 | 0.55 | 1.35 | 0.0002 |
| SPON1.4297.62 | 1.09 | 0.57 | 1.62 | 0.0003 |
| CYR61.6264.9 | 1.04 | 0.56 | 1.51 | 0.0003 |
| SELM.15336.7 | 1.15 | 0.56 | 1.74 | 0.0004 |
| KLB.19557.3 | -1.01 | -1.47 | -0.55 | 0.0004 |
| TNFRSF1A.2654.19 | 1.08 | 0.56 | 1.61 | 0.0004 |
| BOC.4328.2 | 1.02 | 0.55 | 1.49 | 0.0004 |
| LCN2.2836.68 | 1.04 | 0.55 | 1.52 | 0.0004 |
| HSPA13.17515.6 | 1.02 | 0.55 | 1.48 | 0.0004 |
| EMC4.13516.46 | -1.04 | -1.53 | -0.55 | 0.0005 |
| TMEM132B.8890.9 | 1.03 | 0.55 | 1.51 | 0.0005 |
| ACVRL1.16318.12 | 1.10 | 0.54 | 1.65 | 0.0006 |
| F11.2190.55 | -1.01 | -1.47 | -0.54 | 0.0006 |
| SSR1.8106.15 | -1.03 | -1.52 | -0.54 | 0.0006 |
| WIF1.16070.7 | 1.05 | 0.54 | 1.56 | 0.0006 |
| EDN1.6495.14 | 0.98 | 0.54 | 1.42 | 0.0006 |
| KCNMB3.8905.20 | -1.06 | -1.58 | -0.54 | 0.0007 |
| DCN.2666.53 | 0.96 | 0.53 | 1.39 | 0.0008 |
| STC1.4930.21 | 1.02 | 0.53 | 1.50 | 0.0008 |
| PCSK9.5231.79 | -0.98 | -1.44 | -0.53 | 0.0008 |
| APOF.12370.30 | 1.01 | 0.53 | 1.48 | 0.0009 |
| RNASE6.5646.20 | 1.03 | 0.53 | 1.53 | 0.0009 |
| IL1RL1.4234.8 | 0.99 | 0.52 | 1.46 | 0.0010 |
| CSF1R.13682.47 | 0.99 | 0.52 | 1.47 | 0.0011 |
| HIST2H2BE.14143.8 | 0.96 | 0.52 | 1.40 | 0.0011 |
| NCAM2.6507.16 | 1.04 | 0.52 | 1.56 | 0.0011 |
| KLK10.6227.1 | 1.03 | 0.52 | 1.53 | 0.0011 |
| FSTL1.13112.179 | 0.98 | 0.52 | 1.44 | 0.0011 |
| MGAT2.6909.40 | -1.00 | -1.48 | -0.52 | 0.0011 |
| LBP.3074.6 | 0.98 | 0.51 | 1.44 | 0.0014 |
| CDCP1.16818.200 | 1.04 | 0.52 | 1.56 | 0.0014 |
| NLGN1.15620.4 | 1.01 | 0.51 | 1.51 | 0.0014 |
| AIF1L.18871.24 | 1.06 | 0.51 | 1.62 | 0.0015 |
| FGA.FGB.FGG.2796.62 | 0.99 | 0.51 | 1.46 | 0.0017 |
| CSF1.3738.54 | 1.03 | 0.51 | 1.55 | 0.0017 |
| NPS.6390.18 | -0.99 | -1.47 | -0.51 | 0.0018 |
| GAS6.15391.114 | 0.97 | 0.51 | 1.43 | 0.0018 |
| SRSF7.12987.12 | 1.01 | 0.50 | 1.53 | 0.0020 |
| ENTPD6.8932.1 | -0.99 | -1.49 | -0.50 | 0.0021 |
| NOTCH3.5108.72 | 1.00 | 0.50 | 1.50 | 0.0021 |
| POSTN.3457.57 | 0.96 | 0.50 | 1.43 | 0.0022 |
| ARFGAP1.11556.19 | -1.02 | -1.54 | -0.50 | 0.0022 |
| PRKG1.13067.5 | -1.05 | -1.61 | -0.50 | 0.0022 |
| GRB14.13628.58 | -1.04 | -1.58 | -0.50 | 0.0023 |
| SPOCK2.5491.12 | 0.96 | 0.50 | 1.42 | 0.0023 |
| CPN2.6415.90 | -0.98 | -1.46 | -0.50 | 0.0023 |
| FJX1.7921.65 | 1.01 | 0.50 | 1.52 | 0.0024 |
| OIT3.6296.36 | 0.96 | 0.49 | 1.42 | 0.0025 |
| EPHA2.4834.61 | 1.00 | 0.49 | 1.52 | 0.0027 |
| APOA5.15363.32 | -0.95 | -1.41 | -0.49 | 0.0027 |
| NPFF.5617.41 | -0.99 | -1.49 | -0.49 | 0.0027 |
| NPTX2.6521.35 | -0.97 | -1.46 | -0.49 | 0.0027 |
| CCDC126.6388.21 | -0.94 | -1.39 | -0.49 | 0.0028 |
| CLSTN3.6291.55 | 1.01 | 0.49 | 1.52 | 0.0028 |
| SORCS1.15636.49 | 0.96 | 0.49 | 1.43 | 0.0028 |
| IL19.3035.80 | 0.94 | 0.49 | 1.39 | 0.0028 |
| ATP1B1.13392.13 | -0.94 | -1.39 | -0.49 | 0.0028 |
| ADGRF5.6409.57 | -0.97 | -1.46 | -0.49 | 0.0031 |
| UMOD.9451.20 | 0.97 | 0.48 | 1.45 | 0.0032 |
| SEMA6B.5121.3 | 0.96 | 0.48 | 1.44 | 0.0033 |
| CRIM1.15492.1 | 0.99 | 0.48 | 1.50 | 0.0034 |
| ENPP2.16892.23 | 0.94 | 0.48 | 1.40 | 0.0035 |
| MGAT4A.9392.43 | -0.96 | -1.44 | -0.48 | 0.0035 |
| PIANP.9599.6 | 0.98 | 0.48 | 1.49 | 0.0037 |
| PTPRU.8337.65 | 0.94 | 0.47 | 1.40 | 0.0043 |
| CHFR.11320.29 | -0.95 | -1.43 | -0.47 | 0.0045 |
| NRAS.10531.18 | 0.93 | 0.47 | 1.39 | 0.0052 |
| EFNA2.14124.6 | 0.99 | 0.46 | 1.53 | 0.0054 |
| CLEC7A.3603.60 | 0.91 | 0.46 | 1.37 | 0.0064 |
| CTSB.8007.19 | 0.90 | 0.46 | 1.35 | 0.0066 |
| FAM171A2.13479.8 | -0.97 | -1.47 | -0.46 | 0.0068 |
| SRL.10940.25 | 0.97 | 0.45 | 1.49 | 0.0071 |
| CTSS.3181.50 | 0.99 | 0.45 | 1.53 | 0.0077 |
| ATP1B2.7218.87 | 0.93 | 0.45 | 1.41 | 0.0077 |
| VWC2.15308.108 | 0.98 | 0.45 | 1.50 | 0.0080 |
| CHST11.7779.86 | 0.96 | 0.45 | 1.46 | 0.0080 |
| NR1H2.9016.12 | -1.00 | -1.55 | -0.45 | 0.0081 |
| MATN3.19361.78 | 0.94 | 0.45 | 1.42 | 0.0087 |
| LRP4.19558.10 | 0.97 | 0.44 | 1.50 | 0.0090 |
| CXCL12.3516.60 | 0.95 | 0.44 | 1.45 | 0.0093 |
| KREMEN1.17331.138 | 0.92 | 0.45 | 1.40 | 0.0093 |
| CD59.11514.196 | 0.96 | 0.44 | 1.47 | 0.0098 |
| TRABD.11262.39 | -0.99 | -1.55 | -0.44 | 0.0099 |
| COMT.18382.109 | -0.89 | -1.33 | -0.44 | 0.0099 |
| CXCL13.3487.32 | 0.91 | 0.44 | 1.38 | 0.0102 |
| CILP2.8841.65 | -0.93 | -1.43 | -0.44 | 0.0102 |
| ALDH5A1.17792.158 | -0.92 | -1.40 | -0.44 | 0.0112 |
| ARL15.18411.83 | -0.93 | -1.43 | -0.44 | 0.0116 |
| SPARCL1.4467.49 | 0.92 | 0.44 | 1.40 | 0.0117 |
| DHRS9.17467.1 | -0.95 | -1.48 | -0.43 | 0.0124 |
| SERPINA11.9002.36 | 0.90 | 0.43 | 1.37 | 0.0128 |
| DCTPP1.4314.12 | 0.92 | 0.43 | 1.41 | 0.0130 |
| BCAM.2816.50 | 0.96 | 0.43 | 1.49 | 0.0136 |
| ALB.18380.78 | 0.92 | 0.43 | 1.40 | 0.0144 |
| ITIH3.7145.1 | 0.90 | 0.43 | 1.37 | 0.0144 |
| HIST3H2BB.18823.52 | 0.83 | 0.43 | 1.23 | 0.0156 |
| ASB9.19601.15 | 1.06 | 0.40 | 1.72 | 0.0157 |
| HSPB6.19127.1 | 0.96 | 0.42 | 1.50 | 0.0161 |
| C1QBP.4967.1 | -0.93 | -1.45 | -0.41 | 0.0184 |
| COX6C.8903.1 | -0.84 | -1.27 | -0.42 | 0.0191 |
| GALE.11457.53 | -0.89 | -1.36 | -0.42 | 0.0193 |
| ATF6B.11387.3 | -0.87 | -1.34 | -0.41 | 0.0217 |
| IL26.16760.2 | -0.88 | -1.36 | -0.41 | 0.0222 |
| FMOD.6367.66 | 0.87 | 0.41 | 1.32 | 0.0224 |
| CTSF.9212.22 | -0.89 | -1.37 | -0.40 | 0.0251 |
| SOCS3.11440.58 | -0.87 | -1.34 | -0.40 | 0.0257 |
| SPON2.8099.42 | 0.92 | 0.40 | 1.45 | 0.0272 |
| PTPRJ.8250.2 | -0.91 | -1.42 | -0.39 | 0.0294 |
| VAV3.9830.109 | -0.92 | -1.45 | -0.39 | 0.0294 |
| TNFSF15.2968.61 | 0.95 | 0.38 | 1.52 | 0.0306 |
| IL18BP.3073.51 | 0.89 | 0.39 | 1.39 | 0.0306 |
| F13A1.F13B.16927.9 | -0.84 | -1.28 | -0.40 | 0.0314 |
| S100A4.14116.129 | -0.87 | -1.35 | -0.39 | 0.0318 |
| RNF122.11160.56 | -0.94 | -1.49 | -0.38 | 0.0322 |
| IL1B.3037.62 | 0.85 | 0.39 | 1.30 | 0.0327 |
| PCDHGA12.6938.21 | 0.85 | 0.39 | 1.32 | 0.0338 |
| FLT4.16035.8 | 0.89 | 0.39 | 1.39 | 0.0359 |
| INA.11436.6 | 0.88 | 0.38 | 1.38 | 0.0381 |
| PLEKHA1.12459.13 | -0.88 | -1.37 | -0.38 | 0.0381 |
| CRLF1.14747.9 | 0.87 | 0.38 | 1.35 | 0.0391 |
| SERPINF2.3024.18 | -0.93 | -1.48 | -0.37 | 0.0391 |
| SET.5364.7 | -0.90 | -1.42 | -0.38 | 0.0404 |
| CKAP2.5345.51 | -0.88 | -1.39 | -0.38 | 0.0415 |
| TFF1.9185.15 | 0.87 | 0.38 | 1.36 | 0.0415 |
| PCDHGC3.7859.21 | -0.91 | -1.45 | -0.37 | 0.0416 |
| FGL1.5581.28 | 0.82 | 0.38 | 1.25 | 0.0420 |
| CLIC2.17837.5 | 0.85 | 0.38 | 1.33 | 0.0431 |
| AGRP.2813.11 | 0.86 | 0.38 | 1.34 | 0.0440 |
| KRTAP24.14615.46 | 0.84 | 0.38 | 1.31 | 0.0467 |
| SERPINA1.3580.25 | 0.84 | 0.37 | 1.31 | 0.0489 |

## Table 1.4 E/e’ ratio

| **Biomarker** | **Estimate** | **CI - Lower** | **CI - Upper** | **P-value** |
| --- | --- | --- | --- | --- |
| NPPA.5443.62 | 4.31 | 3.14 | 5.48 | 0.0000 |
| CHRDL1.3362.61 | 4.41 | 2.83 | 5.98 | 0.0000 |
| ANGPT2.2602.2 | 3.61 | 2.40 | 4.82 | 0.0000 |
| ENTPD5.4437.56 | -3.70 | -4.99 | -2.42 | 0.0000 |
| COL28A1.10702.1 | 3.82 | 2.43 | 5.20 | 0.0000 |
| LUM.13114.50 | 3.78 | 2.41 | 5.15 | 0.0000 |
| FCN3.5462.62 | -3.83 | -5.23 | -2.42 | 0.0000 |
| RSPO4.8464.31 | 4.05 | 2.52 | 5.59 | 0.0000 |
| MTAP.9910.9 | -3.79 | -5.18 | -2.40 | 0.0000 |
| CD93.14136.234 | 3.66 | 2.34 | 4.98 | 0.0000 |
| ROBO2.5116.62 | 3.60 | 2.33 | 4.87 | 0.0000 |
| PTK7.9525.1 | 3.44 | 2.20 | 4.67 | 0.0000 |
| ATP1B1.13392.13 | -3.48 | -4.75 | -2.21 | 0.0000 |
| WFDC1.9316.67 | 3.49 | 2.13 | 4.85 | 0.0000 |
| NOTUM.8252.2 | -3.29 | -4.52 | -2.05 | 0.0000 |
| TIMP2.2278.61 | 3.44 | 2.08 | 4.80 | 0.0000 |
| ADAMTS13.3175.51 | -3.43 | -4.79 | -2.08 | 0.0000 |
| TAGLN.15640.54 | 3.68 | 2.12 | 5.24 | 0.0000 |
| SCARF2.8956.96 | 3.60 | 2.11 | 5.10 | 0.0000 |
| MFAP4.5636.10 | 3.33 | 2.04 | 4.63 | 0.0000 |
| FBLN5.15585.304 | 3.31 | 2.01 | 4.61 | 0.0000 |
| CST5.3803.10 | 3.43 | 2.03 | 4.83 | 0.0000 |
| MMP2.4160.49 | 3.36 | 1.98 | 4.74 | 0.0000 |
| PLA2G12B.9380.2 | -3.16 | -4.44 | -1.88 | 0.0000 |
| GAS1.5463.22 | 3.33 | 1.88 | 4.78 | 0.0000 |
| CYR61.6264.9 | 3.17 | 1.85 | 4.48 | 0.0000 |
| HSPG2.15626.223 | 3.01 | 1.81 | 4.22 | 0.0000 |
| MAP3K3.12990.39 | 3.08 | 1.81 | 4.35 | 0.0000 |
| PXDN.13463.1 | 3.23 | 1.85 | 4.62 | 0.0000 |
| KERA.10758.2 | 3.05 | 1.79 | 4.31 | 0.0000 |
| NPPB.7655.11 | 3.40 | 2.02 | 4.78 | 0.0000 |
| CCL14.2900.53 | 3.12 | 1.76 | 4.48 | 0.0000 |
| DNAJB12.8006.12 | 3.19 | 1.75 | 4.62 | 0.0000 |
| NPDC1.10424.31 | 3.10 | 1.72 | 4.49 | 0.0000 |
| NRP2.15387.44 | 2.97 | 1.67 | 4.27 | 0.0000 |
| CST3.2609.59 | 3.10 | 1.68 | 4.52 | 0.0000 |
| LAMA2.LAMB1.LAMC1.18347.15 | 2.99 | 1.67 | 4.30 | 0.0000 |
| CDH7.7959.34 | -2.98 | -4.31 | -1.66 | 0.0000 |
| EFEMP1.8480.29 | 3.16 | 1.68 | 4.64 | 0.0000 |
| LRP10.16610.13 | 3.08 | 1.66 | 4.51 | 0.0000 |
| FSTL3.3438.10 | 3.02 | 1.63 | 4.41 | 0.0000 |
| ESM1.3805.16 | 2.94 | 1.61 | 4.28 | 0.0000 |
| RBP7.14208.3 | 2.87 | 1.60 | 4.15 | 0.0000 |
| NBL1.2944.66 | 3.14 | 1.63 | 4.64 | 0.0000 |
| WFDC2.11388.75 | 3.05 | 1.60 | 4.50 | 0.0000 |
| CELA1.6107.3 | 2.91 | 1.58 | 4.25 | 0.0000 |
| LTBP4.13133.73 | 2.98 | 1.58 | 4.38 | 0.0000 |
| EMC4.13516.46 | -2.95 | -4.33 | -1.58 | 0.0000 |
| BMPER.15368.3 | 2.98 | 1.58 | 4.38 | 0.0000 |
| MYL7.19296.51 | -3.03 | -4.48 | -1.57 | 0.0000 |
| TMEM132D.13416.8 | 2.88 | 1.55 | 4.21 | 0.0000 |
| ROR2.7861.9 | 2.98 | 1.54 | 4.42 | 0.0000 |
| IBSP.3415.61 | 2.72 | 1.49 | 3.96 | 0.0000 |
| INHBB.13676.46 | 2.82 | 1.49 | 4.16 | 0.0000 |
| ITLN1.18830.1 | 2.96 | 1.50 | 4.41 | 0.0000 |
| MINPP1.5586.66 | 2.77 | 1.48 | 4.06 | 0.0000 |
| COL18A1.2201.17 | 2.88 | 1.47 | 4.29 | 0.0000 |
| IGFBP4.2950.57 | 2.90 | 1.46 | 4.34 | 0.0000 |
| SFTPD.19590.46 | 2.76 | 1.43 | 4.10 | 0.0000 |
| STOML1.17344.23 | -2.71 | -4.02 | -1.39 | 0.0000 |
| ACP5.3232.28 | -2.70 | -4.02 | -1.38 | 0.0000 |
| SELM.15336.7 | 2.97 | 1.38 | 4.57 | 0.0000 |
| FIGF.13098.93 | 2.90 | 1.40 | 4.40 | 0.0000 |
| EDN1.6495.14 | 2.64 | 1.37 | 3.91 | 0.0000 |
| EPHA2.4834.61 | 2.78 | 1.36 | 4.20 | 0.0000 |
| ANG.4874.3 | 2.70 | 1.35 | 4.06 | 0.0000 |
| SERPINA7.2706.69 | -2.80 | -4.25 | -1.35 | 0.0000 |
| FCN2.13717.15 | -2.78 | -4.21 | -1.34 | 0.0000 |
| AMH.4923.79 | -2.73 | -4.12 | -1.34 | 0.0000 |
| PLAUR.2652.15 | 2.80 | 1.34 | 4.27 | 0.0000 |
| RBFOX2.11462.8 | 2.64 | 1.32 | 3.95 | 0.0000 |
| MSR1.15533.97 | 2.82 | 1.31 | 4.33 | 0.0000 |
| SPINK7.10974.20 | 2.72 | 1.30 | 4.14 | 0.0000 |
| TNFRSF1A.2654.19 | 2.76 | 1.30 | 4.22 | 0.0000 |
| PPIC.18819.21 | 2.71 | 1.29 | 4.13 | 0.0000 |
| KLK10.6227.1 | 2.70 | 1.29 | 4.10 | 0.0000 |
| HERC5.12934.1 | -2.73 | -4.17 | -1.29 | 0.0000 |
| PCDHGA12.6938.21 | 2.62 | 1.29 | 3.95 | 0.0000 |
| MGP.6520.87 | 2.73 | 1.27 | 4.18 | 0.0000 |
| EGFR.2677.1 | -2.73 | -4.20 | -1.26 | 0.0000 |
| ACVRL1.16318.12 | 2.80 | 1.26 | 4.34 | 0.0000 |
| STC1.4930.21 | 2.60 | 1.26 | 3.93 | 0.0000 |
| DSTN.18883.4 | -2.65 | -4.03 | -1.26 | 0.0000 |
| CFD.2946.52 | 2.67 | 1.25 | 4.10 | 0.0000 |
| ADAMTSL2.6379.62 | 2.45 | 1.25 | 3.65 | 0.0000 |
| FGF23.3807.1 | 2.47 | 1.27 | 3.66 | 0.0000 |
| ASB9.19601.15 | 2.94 | 1.25 | 4.63 | 0.0000 |
| SRL.10940.25 | 2.62 | 1.25 | 3.99 | 0.0000 |
| PRCP.5722.78 | -2.58 | -3.90 | -1.25 | 0.0000 |
| BMP6.8459.10 | 2.54 | 1.24 | 3.85 | 0.0000 |
| CCDC126.6388.21 | -2.53 | -3.83 | -1.24 | 0.0000 |
| HSPA1A.14237.1 | -2.59 | -3.94 | -1.24 | 0.0000 |
| C9.3060.43 | 2.66 | 1.23 | 4.08 | 0.0000 |
| SULT2B1.17209.27 | 2.50 | 1.23 | 3.77 | 0.0000 |
| BCAM.2816.50 | 2.69 | 1.23 | 4.15 | 0.0000 |
| GDF15.4374.45 | 2.64 | 1.23 | 4.04 | 0.0000 |
| CPLX2.15321.8 | 2.62 | 1.22 | 4.01 | 0.0000 |
| RNASE1.7211.2 | 2.64 | 1.23 | 4.05 | 0.0000 |
| UNC5B.15394.79 | 2.68 | 1.20 | 4.15 | 0.0000 |
| VWC2.15308.108 | 2.76 | 1.20 | 4.32 | 0.0000 |
| SPON1.4297.62 | 2.65 | 1.20 | 4.11 | 0.0000 |
| WARS.9870.17 | 2.38 | 1.20 | 3.57 | 0.0000 |
| HYAL1.8309.12 | -2.51 | -3.83 | -1.18 | 0.0000 |
| PCSK1.13388.57 | 2.62 | 1.17 | 4.07 | 0.0000 |
| PRSS2.5034.79 | 2.49 | 1.19 | 3.79 | 0.0000 |
| BCHE.15514.26 | -2.52 | -3.87 | -1.17 | 0.0000 |
| PIANP.9599.6 | 2.55 | 1.16 | 3.95 | 0.0000 |
| BHMT2.14226.120 | 2.22 | 1.16 | 3.27 | 0.0000 |
| GREM2.5598.3 | 2.61 | 1.16 | 4.06 | 0.0000 |
| CD58.10938.13 | 2.41 | 1.15 | 3.67 | 0.0000 |
| NRP1.5542.22 | 2.52 | 1.14 | 3.90 | 0.0000 |
| GM2A.15441.6 | 2.62 | 1.12 | 4.13 | 0.0000 |
| IGFBP7.3320.49 | 2.46 | 1.13 | 3.79 | 0.0000 |
| SVEP1.11109.56 | 2.28 | 1.14 | 3.41 | 0.0000 |
| CCDC80.3234.23 | 2.66 | 1.10 | 4.22 | 0.0000 |
| PAM.5620.13 | 2.46 | 1.12 | 3.79 | 0.0000 |
| TFRC.6895.1 | 2.45 | 1.13 | 3.77 | 0.0000 |
| ATF6B.11387.3 | -2.47 | -3.83 | -1.11 | 0.0000 |
| SRSF7.12987.12 | 2.53 | 1.10 | 3.96 | 0.0000 |
| NMB.9321.400 | 2.70 | 1.09 | 4.32 | 0.0000 |
| TREM1.9266.1 | 2.63 | 1.07 | 4.18 | 0.0000 |
| WNT3A.13236.25 | -2.50 | -3.91 | -1.08 | 0.0000 |
| KREMEN1.17331.138 | 2.41 | 1.07 | 3.76 | 0.0000 |
| SEMA6B.5121.3 | 2.38 | 1.06 | 3.70 | 0.0000 |
| LRRC32.7551.33 | 2.48 | 1.05 | 3.92 | 0.0000 |
| DTX1.11430.49 | -2.38 | -3.70 | -1.05 | 0.0000 |
| CASQ1.11263.57 | -2.51 | -3.99 | -1.02 | 0.0000 |
| COLEC12.5457.5 | 2.43 | 1.03 | 3.82 | 0.0000 |
| OLFML3.8660.5 | 2.40 | 1.02 | 3.78 | 0.0000 |
| NAT1.12632.14 | -2.53 | -4.07 | -1.00 | 0.0000 |
| FLRT2.13122.19 | 2.48 | 1.00 | 3.96 | 0.0000 |
| GUCA2B.6223.5 | 2.59 | 0.98 | 4.21 | 0.0000 |
| AIF1L.18871.24 | 2.54 | 0.97 | 4.11 | 0.0000 |
| COL6A2.16753.46 | 2.45 | 0.99 | 3.91 | 0.0000 |
| PLTP.15475.4 | 2.38 | 0.99 | 3.77 | 0.0000 |
| PSMA5.18925.24 | -2.32 | -3.65 | -1.00 | 0.0000 |
| EFNA4.2614.28 | 2.42 | 0.97 | 3.86 | 0.0000 |
| ENG.4908.6 | 2.27 | 0.98 | 3.56 | 0.0000 |
| EFNA2.14124.6 | 2.42 | 0.96 | 3.88 | 0.0000 |
| HPGDS.12549.33 | -2.31 | -3.65 | -0.97 | 0.0000 |
| KDR.3651.50 | -2.42 | -3.91 | -0.93 | 0.0000 |
| TRA2B.12373.73 | 2.38 | 0.94 | 3.81 | 0.0000 |
| CHST11.7779.86 | 2.33 | 0.95 | 3.72 | 0.0000 |
| PYY.3727.35 | 2.43 | 0.93 | 3.92 | 0.0000 |
| CRIM1.15492.1 | 2.33 | 0.93 | 3.73 | 0.0000 |
| AGER.4125.52 | 2.25 | 0.94 | 3.55 | 0.0000 |
| BMP4.15667.39 | 2.27 | 0.93 | 3.60 | 0.0000 |
| EPO.5813.58 | 2.17 | 0.95 | 3.39 | 0.0000 |
| VIT.6234.74 | 2.44 | 0.90 | 3.98 | 0.0000 |
| ITIH5.8233.2 | 2.33 | 0.92 | 3.75 | 0.0000 |
| IGFLR1.7244.16 | 2.45 | 0.89 | 4.00 | 0.0000 |
| PTGDS.10514.5 | 2.42 | 0.89 | 3.95 | 0.0000 |
| CD209.3029.52 | -2.29 | -3.67 | -0.92 | 0.0000 |
| WIF1.16070.7 | 2.39 | 0.90 | 3.87 | 0.0000 |
| PACAP.16322.10 | 2.38 | 0.90 | 3.86 | 0.0000 |
| EWSR1.12988.49 | 2.38 | 0.89 | 3.88 | 0.0000 |
| APOM.10445.20 | -2.26 | -3.62 | -0.91 | 0.0000 |
| ITIH2.9326.33 | -2.32 | -3.74 | -0.90 | 0.0000 |
| ANTXR2.15559.5 | -2.25 | -3.59 | -0.90 | 0.0000 |
| FSTL1.13112.179 | 2.25 | 0.90 | 3.61 | 0.0000 |
| TNFSF15.2968.61 | 2.42 | 0.86 | 3.97 | 0.0000 |
| PTPRU.8337.65 | 2.24 | 0.90 | 3.58 | 0.0000 |
| CHFR.11320.29 | -2.30 | -3.72 | -0.88 | 0.0000 |
| HSPA13.17515.6 | 2.22 | 0.90 | 3.55 | 0.0000 |
| LBP.3074.6 | 2.18 | 0.88 | 3.48 | 0.0000 |
| MMP7.2789.26 | 2.29 | 0.86 | 3.71 | 0.0000 |
| CHST15.4469.78 | 2.24 | 0.86 | 3.61 | 0.0000 |
| HSPB6.19127.1 | 2.35 | 0.84 | 3.86 | 0.0000 |
| MATN3.19361.78 | 2.19 | 0.87 | 3.51 | 0.0000 |
| CLSTN2.18882.7 | 2.13 | 0.88 | 3.39 | 0.0000 |
| CLIC2.17837.5 | 2.27 | 0.85 | 3.69 | 0.0000 |
| REG3A.15304.1 | 2.31 | 0.84 | 3.78 | 0.0000 |
| EDAR.2977.7 | -2.21 | -3.57 | -0.85 | 0.0000 |
| COMT.18382.109 | -2.15 | -3.45 | -0.86 | 0.0000 |
| SPON2.8099.42 | 2.36 | 0.81 | 3.91 | 0.0000 |
| EFNA5.2615.60 | 2.33 | 0.81 | 3.84 | 0.0000 |
| PRKG1.13067.5 | -2.35 | -3.90 | -0.79 | 0.0000 |
| C11orf87.11116.16 | -2.15 | -3.47 | -0.83 | 0.0000 |
| CLSTN3.6291.55 | 2.37 | 0.81 | 3.93 | 0.0000 |
| CD27.5412.53 | -2.25 | -3.70 | -0.79 | 0.0000 |
| NTNG1.5637.81 | 2.19 | 0.80 | 3.58 | 0.0000 |
| F7.3184.25 | -2.18 | -3.56 | -0.81 | 0.0000 |
| UROS.11248.43 | 2.10 | 0.82 | 3.38 | 0.0000 |
| SURF1.8009.121 | -2.27 | -3.77 | -0.78 | 0.0000 |
| OMD.5358.3 | 2.12 | 0.81 | 3.43 | 0.0000 |
| NTF3.4145.58 | 2.19 | 0.79 | 3.58 | 0.0000 |
| TMED10.6506.54 | 2.25 | 0.77 | 3.72 | 0.0000 |
| CAPG.4968.50 | 2.34 | 0.74 | 3.94 | 0.0000 |
| RNASE6.5646.20 | 2.22 | 0.77 | 3.67 | 0.0000 |
| SLC35G2.13501.10 | -2.18 | -3.59 | -0.76 | 0.0000 |
| DCLK1.17156.72 | 2.16 | 0.76 | 3.57 | 0.0000 |
| ANGPTL4.3796.79 | 2.15 | 0.76 | 3.54 | 0.0000 |
| CTSF.9212.22 | -2.16 | -3.57 | -0.75 | 0.0000 |
| FAM3B.9177.6 | 2.23 | 0.72 | 3.75 | 0.0000 |
| ADGRF5.6409.57 | -2.17 | -3.61 | -0.73 | 0.0000 |
| TST.12663.1 | 2.09 | 0.74 | 3.43 | 0.0000 |
| NPTX2.6521.35 | -2.18 | -3.65 | -0.71 | 0.0000 |
| INA.11436.6 | 2.13 | 0.71 | 3.56 | 0.0000 |
| CTSS.3181.50 | 2.22 | 0.68 | 3.76 | 0.0000 |
| LOXL2.6504.65 | 2.10 | 0.71 | 3.49 | 0.0000 |
| ARFGAP1.11556.19 | -2.17 | -3.65 | -0.69 | 0.0000 |
| YBX1.9751.72 | 2.12 | 0.70 | 3.53 | 0.0000 |
| MRC2.3041.55 | 2.14 | 0.68 | 3.60 | 0.0000 |
| C1QBP.4967.1 | -2.21 | -3.76 | -0.66 | 0.0000 |
| CRP.4337.49 | 2.04 | 0.70 | 3.38 | 0.0000 |
| POSTN.3457.57 | 2.08 | 0.68 | 3.48 | 0.0000 |
| PSMB1.12612.37 | 2.18 | 0.64 | 3.73 | 0.0000 |
| APOA5.15363.32 | -2.04 | -3.39 | -0.69 | 0.0000 |
| GHR.2948.58 | -2.13 | -3.60 | -0.66 | 0.0000 |
| NLGN1.15620.4 | 2.12 | 0.66 | 3.58 | 0.0000 |
| PRSS3.18864.7 | 2.11 | 0.66 | 3.56 | 0.0000 |
| GUCA1A.10008.43 | -2.07 | -3.47 | -0.67 | 0.0000 |
| MET.2837.3 | -2.05 | -3.43 | -0.68 | 0.0000 |
| KLKB1.4152.58 | -2.12 | -3.59 | -0.65 | 0.0000 |
| NPFF.5617.41 | -2.10 | -3.56 | -0.65 | 0.0000 |
| TPMT.11218.84 | -2.14 | -3.65 | -0.63 | 0.0000 |
| HAMP.3504.58 | -2.03 | -3.40 | -0.67 | 0.0000 |
| SIRT2.5030.52 | -2.23 | -3.84 | -0.63 | 0.0000 |
| ADM.7922.5 | 1.87 | 0.70 | 3.04 | 0.0000 |
| UNC5C.5139.32 | 2.14 | 0.61 | 3.67 | 0.0000 |
| PCSK2.6117.4 | 2.00 | 0.65 | 3.34 | 0.0000 |
| ARL11.12433.8 | -2.13 | -3.65 | -0.60 | 0.0000 |
| ENTPD6.8932.1 | -2.10 | -3.60 | -0.59 | 0.0000 |
| CRIP2.9053.16 | 2.08 | 0.60 | 3.57 | 0.0000 |
| C5orf38.6378.2 | -2.02 | -3.44 | -0.61 | 0.0000 |
| PLA2G2A.2692.74 | 1.97 | 0.63 | 3.30 | 0.0000 |
| CD59.11514.196 | 2.04 | 0.60 | 3.47 | 0.0000 |
| FGFR1.5532.53 | 2.00 | 0.61 | 3.39 | 0.0000 |
| TREML2.5736.1 | -1.99 | -3.37 | -0.60 | 0.0000 |
| NOTCH3.5108.72 | 2.02 | 0.58 | 3.45 | 0.0000 |
| REG4.11102.22 | 2.13 | 0.52 | 3.74 | 0.0000 |
| PTPRJ.8250.2 | -2.02 | -3.48 | -0.56 | 0.0000 |
| FLT1.16315.105 | 1.72 | 0.66 | 2.78 | 0.0000 |
| HAVCR2.5134.52 | 2.00 | 0.57 | 3.44 | 0.0000 |
| IGF1R.4232.19 | 1.99 | 0.57 | 3.41 | 0.0000 |
| NFASC.7179.69 | 1.97 | 0.58 | 3.36 | 0.0000 |
| B3GNT2.7980.72 | -1.97 | -3.37 | -0.58 | 0.0000 |
| TRABD.11262.39 | -2.13 | -3.76 | -0.50 | 0.0000 |
| FABP3.5437.63 | 1.99 | 0.56 | 3.42 | 0.0000 |
| CLIC5.12475.48 | 1.98 | 0.57 | 3.40 | 0.0000 |
| FGA.FGB.FGG.2796.62 | 2.05 | 0.51 | 3.58 | 0.0000 |
| AQP4.11363.58 | -1.96 | -3.36 | -0.55 | 0.0000 |
| MENT.5744.12 | -1.99 | -3.43 | -0.54 | 0.0000 |
| GRB14.13628.58 | -2.07 | -3.64 | -0.49 | 0.0000 |
| C6.4127.75 | 1.90 | 0.56 | 3.24 | 0.0000 |
| PCSK9.5231.79 | -1.90 | -3.24 | -0.56 | 0.0000 |
| KCNMB3.8905.20 | -2.03 | -3.55 | -0.50 | 0.0000 |
| JAG1.5092.51 | 1.96 | 0.53 | 3.39 | 0.0000 |
| HSPA1B.18901.26 | 1.91 | 0.54 | 3.28 | 0.0000 |
| TIMD4.15449.33 | 1.87 | 0.56 | 3.18 | 0.0000 |
| BOC.4328.2 | 1.92 | 0.53 | 3.30 | 0.0000 |
| SCN2B.8353.15 | -1.91 | -3.29 | -0.53 | 0.0000 |
| BAG4.12844.10 | 1.84 | 0.56 | 3.13 | 0.0000 |
| OIT3.6296.36 | 1.87 | 0.54 | 3.19 | 0.0000 |
| SIGLEC8.7864.3 | -1.97 | -3.45 | -0.49 | 0.0000 |
| B4GALT2.9595.11 | -1.95 | -3.39 | -0.50 | 0.0000 |
| CSF1R.13682.47 | 1.89 | 0.52 | 3.26 | 0.0000 |
| NPTN.7194.36 | 1.89 | 0.52 | 3.26 | 0.0000 |
| CALCOCO2.12534.10 | 1.87 | 0.52 | 3.22 | 0.0000 |
| S100A4.14116.129 | -1.92 | -3.35 | -0.50 | 0.0000 |
| MAN1A2.9077.10 | -1.91 | -3.31 | -0.51 | 0.0000 |
| ADIPOQ.3554.24 | 1.83 | 0.54 | 3.12 | 0.0000 |
| TNNT2.5315.22 | 1.79 | 0.55 | 3.04 | 0.0000 |
| SH2D3C.12704.26 | -1.96 | -3.44 | -0.47 | 0.0000 |
| HBZ.6919.3 | -1.91 | -3.32 | -0.50 | 0.0000 |
| SOD2.5008.51 | -1.87 | -3.23 | -0.51 | 0.0000 |
| HSPA8.5903.91 | 1.80 | 0.54 | 3.06 | 0.0000 |
| MGAT2.6909.40 | -1.89 | -3.28 | -0.50 | 0.0000 |
| FLRT3.13123.3 | 1.94 | 0.47 | 3.40 | 0.0000 |
| MATN2.3325.2 | 1.95 | 0.47 | 3.44 | 0.0000 |
| CLEC7A.3603.60 | 1.86 | 0.51 | 3.20 | 0.0000 |
| IL19.3035.80 | 1.80 | 0.53 | 3.08 | 0.0000 |
| NELL2.6022.57 | 1.92 | 0.48 | 3.36 | 0.0000 |
| NPS.6390.18 | -1.93 | -3.40 | -0.47 | 0.0000 |
| GALE.11457.53 | -1.86 | -3.22 | -0.50 | 0.0000 |
| CILP2.8841.65 | -1.92 | -3.37 | -0.47 | 0.0000 |
| DSC2.13126.52 | 1.89 | 0.48 | 3.30 | 0.0000 |
| PRSS1.3049.61 | 1.88 | 0.48 | 3.27 | 0.0000 |
| RBBP9.10064.12 | -1.88 | -3.30 | -0.47 | 0.0000 |
| PRSS35.9983.97 | -1.94 | -3.45 | -0.44 | 0.0000 |
| PRSS22.4534.10 | 1.81 | 0.50 | 3.12 | 0.0000 |
| LRRK2.10990.21 | -1.91 | -3.38 | -0.44 | 0.0000 |
| KLK11.2831.29 | 1.82 | 0.48 | 3.17 | 0.0000 |
| LAMC2.9580.5 | 1.84 | 0.47 | 3.21 | 0.0000 |
| AFM.4763.31 | -1.79 | -3.10 | -0.49 | 0.0000 |
| SORCS1.15636.49 | 1.84 | 0.46 | 3.21 | 0.0000 |
| CHST3.7189.55 | 1.86 | 0.45 | 3.26 | 0.0000 |
| KAAG1.19492.5 | -1.77 | -3.04 | -0.49 | 0.0000 |
| UBE2D3.19280.29 | -1.96 | -3.54 | -0.39 | 0.0000 |
| IL1RL1.4234.8 | 1.78 | 0.48 | 3.07 | 0.0000 |
| BAGE3.6442.6 | -1.87 | -3.32 | -0.41 | 0.0000 |
| ENPP2.16892.23 | 1.80 | 0.45 | 3.15 | 0.0000 |
| ATP1B2.7218.87 | 1.84 | 0.43 | 3.26 | 0.0000 |
| SPARCL1.4467.49 | 1.79 | 0.45 | 3.13 | 0.0000 |
| JTB.9038.12 | 1.80 | 0.45 | 3.16 | 0.0000 |
| TPST1.7928.183 | 1.95 | 0.37 | 3.53 | 0.0000 |
| SEMA4A.16915.153 | -1.78 | -3.11 | -0.45 | 0.0000 |
| FAP.5029.3 | -1.84 | -3.28 | -0.41 | 0.0000 |
| CSF1.3738.54 | 1.83 | 0.40 | 3.26 | 0.0001 |
| IL1B.3037.62 | 1.76 | 0.44 | 3.09 | 0.0001 |
| DKK3.3607.71 | 1.85 | 0.38 | 3.33 | 0.0001 |
| FCGR3B.3311.27 | 1.79 | 0.42 | 3.17 | 0.0001 |
| FKBP6.12529.32 | -1.90 | -3.43 | -0.36 | 0.0001 |
| APOF.12370.30 | 1.77 | 0.43 | 3.11 | 0.0001 |
| NAP1L2.13529.39 | -1.89 | -3.44 | -0.35 | 0.0001 |
| DCTPP1.4314.12 | 1.79 | 0.40 | 3.19 | 0.0001 |
| CTRB1.5671.1 | 1.77 | 0.41 | 3.12 | 0.0001 |
| FLT4.16035.8 | 1.78 | 0.40 | 3.16 | 0.0001 |
| HS6ST3.18896.23 | -1.78 | -3.17 | -0.39 | 0.0001 |
| RNASE4.5644.60 | 1.88 | 0.33 | 3.43 | 0.0001 |
| F13B.5658.64 | -1.71 | -2.99 | -0.43 | 0.0001 |
| NR1H2.9016.12 | -1.88 | -3.42 | -0.33 | 0.0001 |
| SH3BP2.7769.29 | -2.03 | -3.81 | -0.25 | 0.0001 |
| PDE1A.5253.1 | 1.78 | 0.37 | 3.19 | 0.0001 |
| SERPINF2.3024.18 | -1.90 | -3.51 | -0.29 | 0.0001 |
| SPP1.13113.7 | 1.71 | 0.41 | 3.01 | 0.0001 |
| TGFB3.3520.58 | 1.84 | 0.32 | 3.35 | 0.0002 |
| METTL1.12514.16 | -1.77 | -3.17 | -0.36 | 0.0002 |
| CHGA.8476.11 | 1.82 | 0.33 | 3.30 | 0.0002 |
| PCSK7.4459.68 | -1.80 | -3.27 | -0.33 | 0.0002 |
| FBLN1.6470.19 | 1.83 | 0.32 | 3.34 | 0.0002 |
| ITIH1.7955.195 | -1.71 | -3.03 | -0.39 | 0.0002 |
| BPIFA1.6473.55 | 1.72 | 0.37 | 3.08 | 0.0002 |
| TCEA1.18204.1 | 1.84 | 0.30 | 3.37 | 0.0002 |
| SOCS3.11440.58 | -1.72 | -3.08 | -0.36 | 0.0002 |
| ST8SIA2.7920.30 | 1.81 | 0.30 | 3.33 | 0.0003 |
| CCK.6918.183 | 1.80 | 0.30 | 3.31 | 0.0003 |
| PRG3.9015.1 | -1.75 | -3.17 | -0.33 | 0.0003 |
| FURIN.6276.16 | 1.71 | 0.35 | 3.08 | 0.0003 |
| FGG.4989.7 | 1.76 | 0.32 | 3.20 | 0.0003 |
| DEPP.7178.59 | -1.80 | -3.30 | -0.29 | 0.0003 |
| QPRT.17789.1 | 1.80 | 0.29 | 3.30 | 0.0003 |
| REPIN1.13554.78 | -1.69 | -3.03 | -0.35 | 0.0004 |
| MXRA7.8005.1 | 1.76 | 0.31 | 3.21 | 0.0004 |
| FCRL6.6617.12 | -1.68 | -2.99 | -0.36 | 0.0004 |
| DPT.4979.34 | 1.88 | 0.21 | 3.56 | 0.0004 |
| FJX1.7921.65 | 1.76 | 0.30 | 3.23 | 0.0004 |
| SERPINA11.9002.36 | 1.70 | 0.33 | 3.08 | 0.0004 |
| ERLEC1.8957.72 | 1.73 | 0.31 | 3.14 | 0.0004 |
| PCDHGA10.6321.65 | 1.59 | 0.38 | 2.81 | 0.0005 |
| ADPGK.6221.1 | 1.69 | 0.31 | 3.08 | 0.0006 |
| CXADR.11204.80 | -1.74 | -3.20 | -0.28 | 0.0006 |
| IL1R1.2991.9 | 1.69 | 0.31 | 3.07 | 0.0006 |
| PCYOX1.6431.68 | -1.69 | -3.09 | -0.30 | 0.0006 |
| CLCA2.8950.4 | -1.79 | -3.34 | -0.24 | 0.0007 |
| F8.13499.30 | 1.74 | 0.26 | 3.22 | 0.0007 |
| TIMP1.2211.9 | 1.72 | 0.28 | 3.16 | 0.0007 |
| C1QTNF1.6304.8 | 1.66 | 0.31 | 3.01 | 0.0007 |
| NRAS.10531.18 | 1.70 | 0.29 | 3.11 | 0.0007 |
| SSR1.8106.15 | -1.71 | -3.15 | -0.27 | 0.0008 |
| BDNF.14047.78 | -1.73 | -3.20 | -0.26 | 0.0008 |
| GEM.12817.1 | -1.68 | -3.08 | -0.28 | 0.0008 |
| IGSF3.9715.15 | 1.64 | 0.31 | 2.98 | 0.0009 |
| LRP4.19558.10 | 1.77 | 0.22 | 3.33 | 0.0009 |
| C1GALT1C1.5735.54 | -1.78 | -3.36 | -0.21 | 0.0009 |
| KLB.19557.3 | -1.65 | -3.01 | -0.30 | 0.0009 |
| SPARC.3043.49 | -1.73 | -3.23 | -0.24 | 0.0010 |
| GLTPD2.7948.129 | -1.68 | -3.09 | -0.26 | 0.0011 |
| BSG.3585.54 | 1.62 | 0.30 | 2.94 | 0.0012 |
| SLPI.4413.3 | 1.78 | 0.18 | 3.38 | 0.0012 |
| SIGLEC15.10847.1 | 1.68 | 0.26 | 3.10 | 0.0012 |
| HDGF.16758.96 | 1.72 | 0.23 | 3.21 | 0.0012 |
| PPP1R1A.17706.4 | 1.68 | 0.25 | 3.10 | 0.0012 |
| CXCL10.4141.79 | 1.64 | 0.27 | 3.01 | 0.0013 |
| ATOX1.19233.75 | 1.86 | 0.13 | 3.59 | 0.0013 |
| NETO1.15298.199 | 1.60 | 0.29 | 2.91 | 0.0013 |
| CAMK1.3592.4 | -1.73 | -3.25 | -0.21 | 0.0013 |
| DLK2.9359.9 | 1.70 | 0.23 | 3.17 | 0.0013 |
| AMY1A.7918.114 | 1.63 | 0.27 | 2.99 | 0.0014 |
| ADAM23.7049.2 | 1.69 | 0.23 | 3.16 | 0.0014 |
| DCN.2666.53 | 1.57 | 0.30 | 2.83 | 0.0015 |
| GXYLT1.8229.1 | -1.60 | -2.92 | -0.28 | 0.0015 |
| FTL.15324.58 | -1.63 | -2.99 | -0.26 | 0.0016 |
| RBP5.19241.31 | 1.69 | 0.22 | 3.16 | 0.0016 |
| PHPT1.16882.27 | 1.68 | 0.22 | 3.14 | 0.0016 |
| IFI16.13940.19 | 1.68 | 0.22 | 3.15 | 0.0016 |
| RPS6KA1.12329.21 | -1.70 | -3.21 | -0.19 | 0.0018 |
| PPIB.4718.5 | 1.55 | 0.27 | 2.83 | 0.0024 |
| TMEM132B.8890.9 | 1.60 | 0.24 | 2.95 | 0.0024 |
| FTH1.FTL.5934.1 | -1.94 | -3.81 | -0.07 | 0.0025 |
| SPINK4.19213.1 | 1.61 | 0.22 | 2.99 | 0.0027 |
| DDR2.15381.45 | 1.54 | 0.27 | 2.80 | 0.0028 |
| CRYZL1.9207.60 | -1.65 | -3.12 | -0.19 | 0.0028 |
| KRTAP24.14615.46 | 1.56 | 0.25 | 2.86 | 0.0029 |
| LEFTY2.15503.15 | 1.72 | 0.12 | 3.32 | 0.0030 |
| CBLN4.5688.65 | 1.61 | 0.21 | 3.01 | 0.0030 |
| RNF122.11160.56 | -1.72 | -3.30 | -0.13 | 0.0031 |
| LCN2.2836.68 | 1.62 | 0.20 | 3.05 | 0.0031 |
| FMOD.6367.66 | 1.58 | 0.22 | 2.94 | 0.0032 |
| DNAJB9.11214.40 | 1.59 | 0.21 | 2.97 | 0.0033 |
| KCNG4.13525.17 | -1.58 | -2.96 | -0.21 | 0.0036 |
| PET117.19303.64 | 1.73 | 0.12 | 3.34 | 0.0036 |
| CCL19.4922.13 | 1.53 | 0.24 | 2.82 | 0.0037 |
| C1orf210.8088.56 | 1.52 | 0.25 | 2.79 | 0.0038 |
| SLAMF1.7953.20 | 1.63 | 0.17 | 3.09 | 0.0038 |
| EGFLAM.12338.27 | 1.63 | 0.16 | 3.11 | 0.0041 |
| SERPINA1.3580.25 | 1.58 | 0.20 | 2.97 | 0.0041 |
| DHRS9.17467.1 | -1.64 | -3.14 | -0.15 | 0.0041 |
| CLEC3B.5701.81 | -1.58 | -2.98 | -0.19 | 0.0046 |
| PDCL2.14192.31 | 1.73 | 0.07 | 3.39 | 0.0046 |
| DDAH1.19392.6 | -1.59 | -3.01 | -0.17 | 0.0048 |
| COL15A1.8974.172 | 1.60 | 0.17 | 3.02 | 0.0049 |
| TNFRSF19.5131.15 | 1.58 | 0.18 | 2.98 | 0.0051 |
| S100A11.14011.17 | 1.64 | 0.12 | 3.16 | 0.0053 |
| APOC1.15364.101 | -1.53 | -2.86 | -0.20 | 0.0054 |
| CCL18.3044.3 | 1.61 | 0.14 | 3.08 | 0.0055 |
| GZMK.9545.156 | -1.55 | -2.90 | -0.19 | 0.0055 |
| FADD.16593.3 | -1.63 | -3.14 | -0.12 | 0.0057 |
| TFF2.9191.8 | 1.59 | 0.15 | 3.03 | 0.0057 |
| SLITRK5.4568.17 | 1.62 | 0.13 | 3.11 | 0.0058 |
| RAB27B.13596.3 | 1.62 | 0.14 | 3.11 | 0.0059 |
| SUMO2.19555.1 | 1.60 | 0.14 | 3.06 | 0.0061 |
| PPCDC.13996.16 | 1.48 | 0.23 | 2.73 | 0.0062 |
| COTL1.4905.63 | -1.64 | -3.19 | -0.09 | 0.0063 |
| CREB3L4.11308.8 | 1.52 | 0.20 | 2.84 | 0.0064 |
| HIST2H2BE.14143.8 | 1.48 | 0.22 | 2.73 | 0.0064 |
| EGLN1.9901.28 | 1.51 | 0.20 | 2.83 | 0.0065 |
| ICAM2.5486.73 | -1.54 | -2.92 | -0.17 | 0.0066 |
| C5.2851.63 | -1.56 | -2.97 | -0.15 | 0.0068 |
| C4orf32.9490.3 | 1.58 | 0.13 | 3.02 | 0.0073 |
| INHBA.INHBB.8467.9 | 1.59 | 0.12 | 3.05 | 0.0074 |
| GNS.3616.3 | 1.55 | 0.15 | 2.94 | 0.0075 |
| IL12B.13733.5 | 1.58 | 0.11 | 3.05 | 0.0081 |
| VAV3.9830.109 | -1.59 | -3.07 | -0.11 | 0.0082 |
| LRP11.15472.16 | 1.60 | 0.09 | 3.11 | 0.0083 |
| BPTF.12482.5 | -1.59 | -3.07 | -0.10 | 0.0088 |
| IL26.16760.2 | -1.54 | -2.95 | -0.14 | 0.0088 |
| PLOD2.6923.1 | 1.49 | 0.18 | 2.80 | 0.0088 |
| REG3G.15476.6 | 1.50 | 0.17 | 2.82 | 0.0089 |
| REG1A.13095.51 | 1.55 | 0.13 | 2.98 | 0.0089 |
| TNFRSF1B.8368.102 | 1.55 | 0.12 | 2.98 | 0.0092 |
| PRSS27.15535.3 | 1.52 | 0.14 | 2.90 | 0.0094 |
| MED4.14021.81 | -1.51 | -2.88 | -0.15 | 0.0094 |
| ZNF41.10003.15 | -1.47 | -2.76 | -0.18 | 0.0095 |
| AGO3.15323.112 | -1.55 | -2.98 | -0.12 | 0.0095 |
| GABARAP.17735.130 | 1.58 | 0.09 | 3.07 | 0.0098 |
| IGHG1.IGHG2.IGHG3.IGHG4.IGK.IGL.2744.57 | 1.58 | 0.09 | 3.07 | 0.0100 |
| CCL28.2890.59 | 1.53 | 0.13 | 2.93 | 0.0100 |
| HFE2.3332.57 | -1.51 | -2.87 | -0.15 | 0.0101 |
| PUF60.10575.31 | 1.53 | 0.13 | 2.93 | 0.0101 |
| TREML1.11147.17 | -1.58 | -3.09 | -0.08 | 0.0103 |
| SDCBP.17806.6 | 1.55 | 0.10 | 2.99 | 0.0107 |
| CRTAC1.5632.6 | -1.59 | -3.10 | -0.07 | 0.0108 |
| CDHR5.9962.1 | -1.50 | -2.85 | -0.14 | 0.0110 |
| RFESD.13603.7 | 1.50 | 0.14 | 2.87 | 0.0111 |
| RCN3.16858.384 | 1.56 | 0.10 | 3.01 | 0.0111 |
| HRAS.18900.37 | -1.56 | -3.05 | -0.08 | 0.0115 |
| FGB.18890.227 | -1.58 | -3.09 | -0.06 | 0.0117 |
| FASLG.3052.8 | -1.54 | -3.00 | -0.09 | 0.0125 |
| CADM1.3326.58 | 1.57 | 0.06 | 3.08 | 0.0126 |
| CHCHD10.11270.17 | 1.53 | 0.09 | 2.97 | 0.0128 |
| TP53I11.13022.20 | -1.51 | -2.92 | -0.11 | 0.0129 |
| CALCB.17170.15 | 1.37 | 0.21 | 2.53 | 0.0129 |
| LRIG3.3322.52 | -1.49 | -2.86 | -0.12 | 0.0129 |
| TMED2.10761.5 | 1.52 | 0.09 | 2.96 | 0.0132 |
| DEFA1.19579.5 | -1.51 | -2.91 | -0.10 | 0.0136 |
| PDCD5.12517.52 | 1.56 | 0.05 | 3.08 | 0.0136 |
| NAALADL1.17505.125 | -1.59 | -3.16 | -0.02 | 0.0137 |
| ROBO1.5740.17 | 1.56 | 0.06 | 3.05 | 0.0141 |
| C1QTNF5.7810.20 | 1.46 | 0.14 | 2.79 | 0.0142 |
| BMP1.3348.49 | -1.49 | -2.88 | -0.10 | 0.0150 |
| C7.2888.49 | 1.54 | 0.06 | 3.01 | 0.0153 |
| NAGPA.11208.15 | -1.50 | -2.91 | -0.09 | 0.0155 |
| DNAJB4.18884.22 | 1.51 | 0.08 | 2.93 | 0.0157 |
| TPST2.8024.64 | -1.57 | -3.11 | -0.02 | 0.0161 |
| CDON.4541.49 | -1.50 | -2.91 | -0.09 | 0.0161 |
| PTK2B.8918.64 | -1.59 | -3.17 | -0.01 | 0.0161 |
| FGFBP3.11219.95 | 1.45 | 0.13 | 2.76 | 0.0162 |
| FABP1.11516.7 | 1.52 | 0.06 | 2.99 | 0.0162 |
| APOA1.2750.3 | -1.47 | -2.84 | -0.11 | 0.0162 |
| HLA.DQA2.7757.5 | 1.45 | 0.13 | 2.76 | 0.0163 |
| KRT20.12975.11 | 1.53 | 0.05 | 3.00 | 0.0170 |
| GLUL.19238.12 | 1.48 | 0.10 | 2.86 | 0.0171 |
| CA9.3798.71 | 1.49 | 0.08 | 2.90 | 0.0176 |
| LOXL3.15427.35 | -1.53 | -3.03 | -0.04 | 0.0185 |
| MYL6B.14227.21 | 1.35 | 0.19 | 2.51 | 0.0186 |
| WNT5A.13268.45 | -1.52 | -2.99 | -0.04 | 0.0190 |
| WISP2.6392.7 | 1.68 | -0.13 | 3.48 | 0.0193 |
| AIMP1.2714.78 | -1.44 | -2.78 | -0.10 | 0.0199 |
| NTN4.3327.27 | 1.43 | 0.12 | 2.73 | 0.0199 |
| DVL2.13575.40 | -1.44 | -2.78 | -0.10 | 0.0200 |
| ARHGAP30.12807.89 | 1.42 | 0.12 | 2.72 | 0.0207 |
| MMP13.4925.54 | 1.44 | 0.10 | 2.77 | 0.0207 |
| ADH4.8325.37 | -1.47 | -2.87 | -0.07 | 0.0207 |
| TDO2.9880.33 | -1.48 | -2.91 | -0.05 | 0.0219 |
| DNAI1.11396.39 | -1.42 | -2.73 | -0.11 | 0.0221 |
| PTPRS.6049.64 | -1.49 | -2.94 | -0.04 | 0.0224 |
| HSF1.11616.9 | 1.49 | 0.04 | 2.94 | 0.0227 |
| PSIP1.17176.13 | 1.49 | 0.04 | 2.93 | 0.0229 |
| ARHGEF2.12848.9 | -1.37 | -2.61 | -0.13 | 0.0247 |
| HS6ST1.5465.32 | -1.49 | -2.94 | -0.03 | 0.0252 |
| FAM177A1.8039.41 | -1.51 | -3.00 | -0.01 | 0.0252 |
| PCSK1N.9391.60 | 1.48 | 0.04 | 2.92 | 0.0252 |
| SIRPA.5430.66 | 1.42 | 0.09 | 2.75 | 0.0255 |
| AGT.3484.60 | -1.38 | -2.65 | -0.12 | 0.0255 |
| BTD.15644.1 | -1.47 | -2.89 | -0.04 | 0.0256 |
| LGR5.16296.43 | -1.49 | -2.97 | -0.02 | 0.0256 |
| TWSG1.9234.8 | 1.45 | 0.05 | 2.85 | 0.0257 |
| GCK.12960.9 | -1.49 | -2.98 | -0.01 | 0.0266 |
| REG1B.16770.3 | 1.53 | -0.02 | 3.07 | 0.0268 |
| IMPAD1.9231.23 | -1.47 | -2.92 | -0.02 | 0.0271 |
| COL11A2.11278.4 | 1.44 | 0.05 | 2.83 | 0.0273 |
| CPN2.6415.90 | -1.47 | -2.91 | -0.03 | 0.0275 |
| CHAD.13460.4 | 1.41 | 0.08 | 2.73 | 0.0279 |
| RARRES2.3079.62 | 1.50 | -0.01 | 3.01 | 0.0286 |
| TMPRSS11D.6547.83 | -1.46 | -2.90 | -0.03 | 0.0287 |
| DIXDC1.13441.30 | -1.44 | -2.84 | -0.05 | 0.0289 |
| PTGES3.17154.2 | 1.40 | 0.08 | 2.71 | 0.0290 |
| DEFA5.16785.45 | 1.45 | 0.04 | 2.86 | 0.0291 |
| IGFBP2.8469.41 | 1.41 | 0.07 | 2.76 | 0.0298 |
| COPS7B.12384.92 | -1.42 | -2.79 | -0.05 | 0.0301 |
| NRSN1.11654.77 | -1.56 | -3.20 | 0.08 | 0.0304 |
| CST2.4324.33 | 1.52 | -0.05 | 3.09 | 0.0310 |
| GALNT3.6593.5 | 1.48 | -0.01 | 2.97 | 0.0316 |
| ARL6.18415.16 | -1.50 | -3.04 | 0.04 | 0.0330 |
| CCL5.5480.49 | -1.50 | -3.03 | 0.03 | 0.0331 |
| MTX2.8839.4 | -1.55 | -3.20 | 0.10 | 0.0334 |
| CREG1.9357.4 | -1.48 | -3.00 | 0.03 | 0.0355 |
| CCL15.18289.16 | 1.43 | 0.02 | 2.84 | 0.0358 |
| SPATA20.11117.2 | -1.41 | -2.79 | -0.03 | 0.0366 |
| IL11.4493.92 | 1.33 | 0.10 | 2.57 | 0.0366 |
| HS3ST5.10731.10 | -1.43 | -2.85 | -0.01 | 0.0384 |
| ALDH1A3.9835.16 | -1.47 | -2.97 | 0.04 | 0.0393 |
| PLEK.7875.86 | -1.49 | -3.03 | 0.05 | 0.0395 |
| PITPNA.9934.29 | 1.45 | -0.01 | 2.90 | 0.0395 |
| LOC652493.6561.77 | -1.47 | -2.97 | 0.04 | 0.0398 |
| KIR2DS2.10428.1 | 1.40 | 0.02 | 2.78 | 0.0401 |
| SWAP70.13552.7 | 1.39 | 0.04 | 2.74 | 0.0407 |
| CDC25B.12427.8 | -1.45 | -2.94 | 0.03 | 0.0410 |
| INSL5.10462.14 | 1.40 | 0.02 | 2.79 | 0.0413 |
| BCL2A1.3413.50 | -1.50 | -3.08 | 0.08 | 0.0414 |
| RAB2B.19253.82 | -1.47 | -3.00 | 0.05 | 0.0417 |
| TMPO.8265.225 | 1.42 | 0.00 | 2.83 | 0.0417 |
| SLITRK4.7139.14 | 1.56 | -0.15 | 3.26 | 0.0421 |
| PPP1R2.19152.4 | -1.45 | -2.93 | 0.03 | 0.0421 |
| RNF8.14663.44 | -1.49 | -3.06 | 0.08 | 0.0433 |
| SMOC2.15635.4 | 1.41 | 0.00 | 2.82 | 0.0433 |
| TNFRSF11B.8304.50 | 1.49 | -0.08 | 3.05 | 0.0433 |
| TIMP3.2480.58 | -1.44 | -2.92 | 0.03 | 0.0446 |
| S100A12.5852.6 | 1.39 | 0.02 | 2.77 | 0.0446 |
| PRSS37.5653.23 | 1.42 | -0.02 | 2.86 | 0.0453 |
| PCDHAC1.7193.98 | -1.45 | -2.96 | 0.05 | 0.0468 |
| SUMF2.6069.71 | 1.37 | 0.02 | 2.73 | 0.0472 |
| CDH3.2643.57 | -1.34 | -2.64 | -0.05 | 0.0482 |
| GLRX.18386.36 | 1.44 | -0.05 | 2.94 | 0.0493 |
| MAP2K4.5242.37 | -1.47 | -3.04 | 0.09 | 0.0499 |

# Supplementary table 2: Circulation proteins in relation to elevated LAP

| **Biomarker** | **OR** | **95% CI** | **P-value** |
| --- | --- | --- | --- |
| Cystatin-D | 3.31 | 2.01-5.85 | 0.008 |
| EGF-containing fibulin-like extracellular matrix protein 1 | 3.04 | 1.84-5.36 | 0.010 |
| Fibulin-5 | 2.96 | 1.87-4.97 | 0.008 |
| DnaJ homolog subfamily B member 12 | 2.93 | 1.88-4.87 | 0.008 |
| Inactive tyrosine-protein kinase 7 | 2.93 | 1.86-4.91 | 0.008 |
| N-terminal pro-BNP | 2.82 | 1.84-4.54 | 0.008 |
| Microfibril-associated glycoprotein 4 | 2.74 | 1.78-4.44 | 0.008 |
| Cystatin-C | 2.73 | 1.79-4.40 | 0.008 |
| Chordin-like protein 1 | 2.72 | 1.72-4.52 | 0.010 |
| Ribonuclease pancreatic | 2.71 | 1.72-4.51 | 0.010 |
| Growth/differentiation factor 15 | 2.68 | 1.74-4.37 | 0.010 |
| Peroxidasin homolog | 2.67 | 1.74-4.28 | 0.008 |
| Fibroblast growth factor 23 | 2.66 | 1.63-4.84 | 0.040 |
| Follistatin-related protein 3 | 2.63 | 1.70-4.27 | 0.010 |
| Atrial natriuretic factor | 2.62 | 1.75-4.16 | 0.008 |
| Spondin-1 | 2.62 | 1.69-4.30 | 0.010 |
| Sushi. von Willebrand factor type A. EGF and pentraxin domain-containing protein 1 | 2.61 | 1.61-4.56 | 0.028 |
| Collagen alpha-1(XXVIII) chain | 2.56 | 1.68-4.10 | 0.010 |
| Roundabout homolog 2 | 2.49 | 1.64-4.00 | 0.012 |
| Calsyntenin-3 | 2.48 | 1.56-4.15 | 0.028 |
| WAP four-disulfide core domain protein 2 | 2.45 | 1.60-3.91 | 0.013 |
| WAP four-disulfide core domain protein 1 | 2.43 | 1.60-3.87 | 0.013 |
| Scavenger receptor class F member 2 | 2.38 | 1.58-3.74 | 0.013 |
| Endostatin | 2.35 | 1.58-3.65 | 0.012 |
| Trypsin-2 | 2.35 | 1.59-3.62 | 0.010 |
| Tumor necrosis factor ligand superfamily member 15 | 2.35 | 1.52-3.82 | 0.027 |
| Lumican | 2.33 | 1.52-3.74 | 0.026 |
| R-spondin-4 | 2.33 | 1.53-3.71 | 0.023 |
| Ganglioside GM2 activator | 2.26 | 1.49-3.58 | 0.026 |
| Lysozyme C | 2.25 | 1.54-3.42 | 0.012 |
| Angiogenin | 2.23 | 1.53-3.39 | 0.012 |
| Insulin-like growth factor-binding protein 4 | 2.23 | 1.50-3.46 | 0.021 |
| Peptide YY | 2.22 | 1.48-3.49 | 0.027 |
| Tumor necrosis factor receptor superfamily member 1A | 2.19 | 1.43-3.48 | 0.046 |
| ADAMTS-like protein 2 | 2.17 | 1.50-3.28 | 0.014 |
| Dermatopontin | 2.17 | 1.42-3.45 | 0.048 |
| 72 kDa type IV collagenase | 2.16 | 1.46-3.34 | 0.027 |
| Antileukoproteinase | 2.14 | 1.45-3.27 | 0.026 |
| Serine/arginine-rich splicing factor 7 | 2.12 | 1.44-3.24 | 0.027 |
| Transferrin receptor protein 1 | 2.10 | 1.45-3.18 | 0.025 |
| Angiopoietin-2 | 2.04 | 1.41-3.06 | 0.028 |
| Dual specificity mitogen-activated protein kinase kinase 4 | 0.51 | 0.34-0.74 | 0.047 |
| Protein DEPP | 0.50 | 0.34-0.72 | 0.028 |
| Palmitoleoyl-protein carboxylesterase NOTUM | 0.50 | 0.33-0.72 | 0.031 |
| Group XIIB secretory phospholipase A2-like protein | 0.46 | 0.30-0.68 | 0.023 |
| Anthrax toxin receptor 2 | 0.46 | 0.30-0.67 | 0.017 |
| Ectonucleoside triphosphate diphosphohydrolase 5 | 0.45 | 0.29-0.66 | 0.017 |
| Coiled-coil domain-containing protein 126 | 0.45 | 0.29-0.65 | 0.013 |
| Epidermal growth factor receptor | 0.38 | 0.23-0.58 | 0.010 |
